# Variations in the Appearance and Interpretation of Interpersonal Eye Contact in Social Categorizations and Psychiatric Populations Worldwide: A Scoping Review with a Critical Appraisal of the Literature

**DOI:** 10.3390/ijerph21081092

**Published:** 2024-08-18

**Authors:** Jos Boer, Nynke Boonstra, Linda Kronenberg, Ruben Stekelenburg, Bram Sizoo

**Affiliations:** 1 Department of Neuroscience, UMC Utrecht, Universiteitsweg 100, 3584 CG Utrecht, The Netherlands; tboonst2@umcutrecht.nl; 2 Dimence Groep, Nico Bolkesteinlaan 1, 7416 SB Deventer, The Netherlands; l.kronenberg@dimencegroep.nl; 3 Lectoraat Innovatie van Beweegzorg, University of Applied Sciences Utrecht, Padualaan 101, 3584 CH Utrecht, The Netherlands; ruben.stekelenburg@hu.nl; 4 Department of Clinical Psychology, University of Amsterdam, Nieuwe Achtergracht 129-B, 1018 WS Amsterdam, The Netherlands; b.b.sizoo@uva.nl

**Keywords:** eye contact, social categorization, psychiatric disorder, presentation, interpretation

## Abstract

Background: Eye contact is one of the most fundamental forms of interhuman communication. However, to date, there has been no comprehensive research comparing how eye contact is made and interpreted in all possible populations worldwide. This study presents a summary of the existing literature on these modalities stratified to social categorizations and psychiatric disorders. Method: A scoping review with critical appraisal of the literature according to the Joanna Briggs Institute (JBI) methodology. Databases AnthroSource, Medline, CINAHL, the Psychology and Behavioral Sciences Collection (EBSCO) and PsychInfo were searched. Results: 7068 articles were screened for both the grey literature and reference lists, of which 385 were included, 282 for social categorizations and 103 for psychiatric disorders. In total, 603 thematic clustered outcomes of variations were included. Methodological quality was generally moderate to good. Conclusions: There is a great degree of variation in the presentation and interpretation of eye contact between and within populations. It remains unclear why specific variations occur in populations. Additionally, no gold standard for how eye contact should be used or interpreted emerged from the studies. Further research into the reason for differences in eye contact between and within populations is recommended.

## 1. Introduction

Research on interhuman eye contact has been conducted for millennia in populations with and without psychiatric disorders. So-called “atypical” eye contact is considered a core symptom of some psychiatric disorders. However, the question arises of what can be understood as “normal” eye contact. Eye contact is commonly defined as “a direct look exchanged between two people” [1], yet this explanation leaves much room for interpretation. For example, a direct look is described as a gaze that is a maximum of 37 degrees to the left or right of the other person’s eyes [2], while other research has shown that the experience of a direct look depends on the head direction [3]. It is not clear whether the direction of a gaze is determined only by the position of the pupil relative to the sclera or by the entire eye region, including the eyebrows. Studies have suggested that eye contact only exists when there is also “you-awareness” of the other during the exchange of a direct look [4], that eye contact is a form of mutual transaction [5] or that eye contact implies connectedness [6]. Because of these different approaches to eye contact, the concept may be applied differently, which could impede transferability between studies. To ensure transparency, the current review uses eye contact in the broadest sense: as a mutual gaze between two people.

This mutual gaze is considered fundamental to human communication and is possibly its most important aspect [7,8,9,10]. Human eyes convey a variety of complex social and emotional information [11], and it has been found that the receiver’s perception of mental states is based on expressive eye behavior. It is not only the receiver’s perception that seems to play a role in this process, but also, for example, the viewing direction of the eyes [12]. A contribution to this gaze discrimination was the evolutionary adaptation of depigmentation of the human sclera, in which the dark iris and gaze direction became clearer, allowing further optimization of eye contact in humans [13]. Other influences on how eye contact is interpreted include pupil dilation and constriction [14]; gaze cuing [15]; the amount of blinking [16]; the frequency and duration of a gaze [17,18,19]; and the activity of the brain areas [20], specifically the amygdala, which is involved, among other things, in the interpretation of facial expressions and emotions [21].

Eye contact not only functions to provide information but also influences other domains. Research has shown that meeting a direct gaze affects cognition, for example, mental control, because some cognitive functions, such as mental control, are seen as irrelevant during eye contact [22]. Additionally, a direct gaze has been shown to improve several social cognitive functions, such as face memory [23], joint attention [24] and empathy [25]. Furthermore, eye contact results in affective reactions on the part of the receiver, such as feelings of intimacy, threat and arousal [20,26,27,28]. It also causes judgments of others depending on eye behavior, for instance, lower or higher ratings of potency, intelligence, attractiveness and credibility [29,30,31,32]. Due to the effects that eye contact has on the interaction partners, it also partly determines the content of their relation [33]. As Laing formulates, “[w]hen we make eye contact, we experience a form of interpersonal connection that plays a central role in human social life, communication, and interpersonal understanding [6].

The above shows that the way eye contact is established and how it is interpreted affects both the interaction partners and their mutual relationship. Decades of research have shown that a distinction can be made between normal and atypical eye contact. Atypical eye contact is mainly described in people with psychiatric disorders, such as autism spectrum disorder [34], social anxiety disorder [35,36], schizophrenia [37,38] and bipolar disorder [39]. Atypical features are, for example, staring too long [40], gaze avoidance [41], impairments in recognizing emotional expressions [42], impairments in gaze discrimination [43] and negative feelings that are induced in the receiver because of eye contact [44].

The question may arise of the basis on which these features can be seen as atypical since they also occur in people without psychiatric disorders. For example, major differences have been found between nationalities in how eye contact is presented by the sender and interpreted by the receiver. Studies using eye-tracking methods have shown that East Asians look toward the center of a face, while Westerners alternate their focus between the points of a triangle, formed by both eyes and the mouth, when learning and recognizing faces [45,46]. Australians have a longer duration of eye contact than Japanese [47]. This difference may correspond to the observation that maintaining eye contact is appraised positively by Western Europeans but not by people with an East Asian cultural background [48]. Consistent with this, previous studies have shown that Japanese show less eye contact than Canadians during face-to-face interactions [49,50]. Compared to people from the Caucasus, Asian people have also been found to stare less at each other during social interactions, which has been attributed to the sociocultural norm of “gaze avoidance”, where an averted gaze functions as a sign of respect in Asian cultures [48,51]. In addition to culture, different features of eye contact have also been found in other social categorizations, such as in gender [52], age [53] and personality [54,55].

The above shows that a large amount of research has been conducted into how eye contact is presented and interpreted within various social categorizations and psychiatric disorders. For this scoping review, all these presentations can be understood as either the eye behavior of the sender during interactions or interpretations of eye behavior by the receiver. To gain insight into the overarching variations of presentations and interpretations of eye contact in all human populations worldwide, it is necessary to collect and combine all available research articles that focus on both social categorizations and psychiatric disorders. A preliminary search is performed for existing systematic reviews and scoping reviews on mapping the total forms of presentations of eye contact in adults per social categorization and psychiatric disorder as well as the effects of eye contact on the interaction partner. Searches were performed in the following databases: JBI Evidence Synthesis, Cochrane Database of Systematic Reviews, PubMed and AnthroSource. This process showed that there is currently no review available on this topic. For this study, the scoping review methodology developed by Peters et al. [56] was chosen because of its suitability to locate, review and synthesize the existing literature on the topic through systematic searches [57]. Using inclusion criteria based on the PCC elements (population, concept and context), the following question was used: “What is known from the existing research literature about variations in appearances and interpretations of interpersonal eye contact in social categorizations and psychiatric disorders worldwide?” This question was subsequently divided into two sub-questions: (1) “What is known from the existing research literature about variations in appearances and interpretations of interpersonal eye contact in social categorizations worldwide?” and (2) “What is known from the existing research literature about variations in appearances and interpretations of interpersonal eye contact in persons with psychiatric disorders worldwide?” The aim of this review is to synthesize the relevant literature, which can lead to a better understanding of the variations in the usage and meaning of eye contact worldwide and provide information for future research.

## 2. Method

This research aims to provide a broad overview of how eye contact is presented and interpreted in adults worldwide, both in social categorizations and psychiatric disorders, and to assess the quality of the included articles. The study was conducted according to the Joanna Briggs Institute (JBI) methodology for scoping reviews [56,58] and the provided checklists [59]. For the article, the Preferred Reporting Items for Systematic Reviews and the Meta-Analyses Extension for Scoping Reviews (PRISMA-Scr) guidelines [60] were used.

Prior to the study, the inclusion criteria, search strategy, handling of screening, extraction and synthesis were recorded in an a priori research protocol, which was created according to the PRESS 2015 Guideline Statement [61] and published prior to the review in the Open Science Framework (ID: osf.io/7htsx).

Concerning eligibility criteria, the selected studies for research sub-question 1 included participants in adulthood (18+) from every social categorization worldwide and one of the following three conditions: (1) variations in appearances of interpersonal eye contact in social categorizations, (2) interpretation of variations in appearances of interpersonal eye contact by others or (3) correlations of appearances of interpersonal eye contact and its interpretation by others. The selected studies for research sub-question 2 included participants in adulthood (18+) diagnosed with psychiatric disorders, according to an internationally recognized classification system, and any one of the following three conditions: (1) variations in appearances of interpersonal eye contact in psychiatric disorders, (2) interpretation of variations in appearances of interpersonal eye contact by others or (3) correlations of appearances of interpersonal eye contact and its interpretation by others. For both research sub-questions, an open context, English and Dutch language, and no date limits were applied.

As described in the research protocol, relevant keywords, medical subject headings and search strings were identified for research sub-questions 1 and 2 during search trials in the databases MEDLINE and CINAHL and a peer review assessment by a medical-information officer (TI). Considering that the scoping review was not a comparative study, the search strategy was based on the building-block approach (BBA). See Supplementary Materials, Tables S1 and S2 for an example of the full search strings.

Databases were searched from inception until 25 May 2023. For research sub-question 1, the included databases were AnthroSource, Medline, CINAHL and the Psychology and Behavioral Sciences Collection (EBSCO). For research sub-question 2, the included databases were Medline, CINAHL, the Psychology and Behavioral Sciences Collection and PsycInfo. For searches in the grey literature, the first 10 pages on Google Scholar were used for research sub-questions 1 and 2.

Included characteristics of the sources of evidence for both research sub-questions 1 and 2 were randomized controlled trials (RCTs), non-randomized controlled trials (non-RCTs), quasi-experimental before and after studies, prospective and retrospective cohort studies, case-control studies, analytical cross-sectional studies, qualitative studies and reviews and research articles in the grey literature. In each article, only sub-studies that met the inclusion criteria were included.

For handling and summarizing data variables, a data-extraction form was used after being tested by the research group on 25 articles for usability and as a measurement of assumptions. After approval, two researchers (J.B. and R.S.) independently screened titles and abstracts then full texts.

Data for research sub-questions 1 and 2 were extracted on the following article characteristics: author; year of publication; title; affiliation of the first author; location of study; journal; medium; full reference; type of evidence; study design; sub-study (of each included study; only sub-studies that met the inclusion criteria were included, and if there were multiple outcomes or populations within that sub-study, these outcomes were divided); intervention; measurement instruments; aim of article; number of participants; size of the intervention group; size of the control group; presentation or interpretation; population; age and gender. When data were available, mean age was primarily noted, then range, and when no details were available, the field was rated with “adults.” When characteristics were not available, fields were rated with “unspecified.” If the source was not suitable for specific data, it was rated “not applicable.” See Supplementary Materials, Tables S3 and S4 for templates of the data-extraction form for research sub-questions 1 and 2.

The systematic assessment of evidence by means of a critical appraisal is not mandatory within scoping reviews [62]. However, because of the added value of assessing the trustworthiness, reliability and bias of evidence, a critical appraisal was conducted (by J.B.), according to the JBI criteria [59]. Articles were included if they met more than half of the JBI criteria.

The data on article characteristics were extracted and thematically analyzed. From this process, variations of presentations and interpretations of eye contact in social categorizations were distinguished and clustered for research sub-question 1. Simultaneously, variations of presentations and interpretations of eye contact in psychiatric disorders were identified and clustered for research sub-question 2 (psychiatric traits were assigned to research sub-question 1 due to the lack of an inclusion criterion for official diagnoses). The software programs Endnote version 21.2 and Microsoft Excel version 3208 were used during this process. The article was written using Microsoft Word version 2308 and Zotero 6.

## 3. Results

### 3.1. Selection of Sources of Evidence

#### 3.1.1. Research Sub-Question 1: What Is Known from the Existing Research Literature about Variations in Appearances and Interpretations of Interpersonal Eye Contact in Social Categorizations Worldwide?

**Figure 1:**
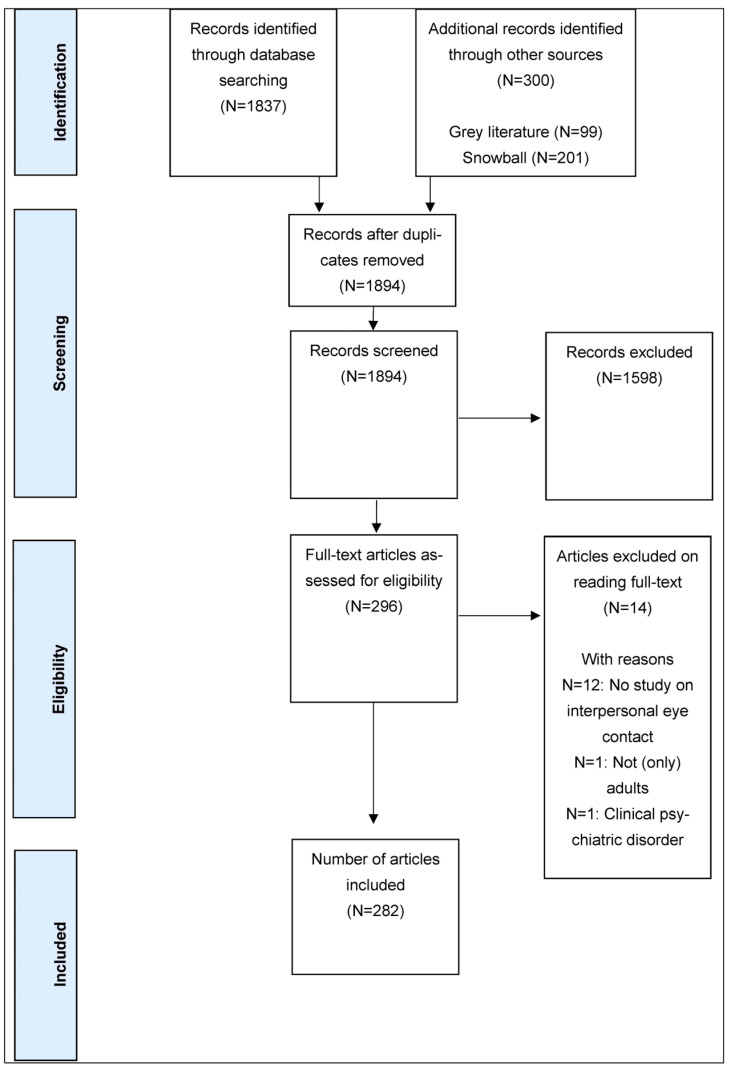
PRISMA flowchart for research question 1 of the Scoping review process.

The databank, grey literature and snowball searches, supplemented with the consultation of a research expert in the field of social categorizations, yielded a total of 2137 potential records for research sub-question 1 (see Figure 1 for PRISMA flowchart). After duplicates were removed, 1894 records were independently screened for eligibility, based on title and abstract. After exclusion, 296 records were assessed further. Finally, 282 records were included: 105 articles (with 118 sub-studies) focusing on presentations of eye contact and 177 articles (with 232 sub-studies) focusing on interpretations of eye contact. During this process, multiple consensus meetings occurred whereby Cohen’s Kappa values for inter-rater reliability [63] ranged between 0.65 (moderate) and 0.94 (almost perfect).

#### 3.1.2. Research Sub-Question 2: What Is Known from the Existing Research Literature about Variations in Appearances and Interpretations of Interpersonal Eye Contact in Persons with Psychiatric Disorders Worldwide?

**Figure 2:**
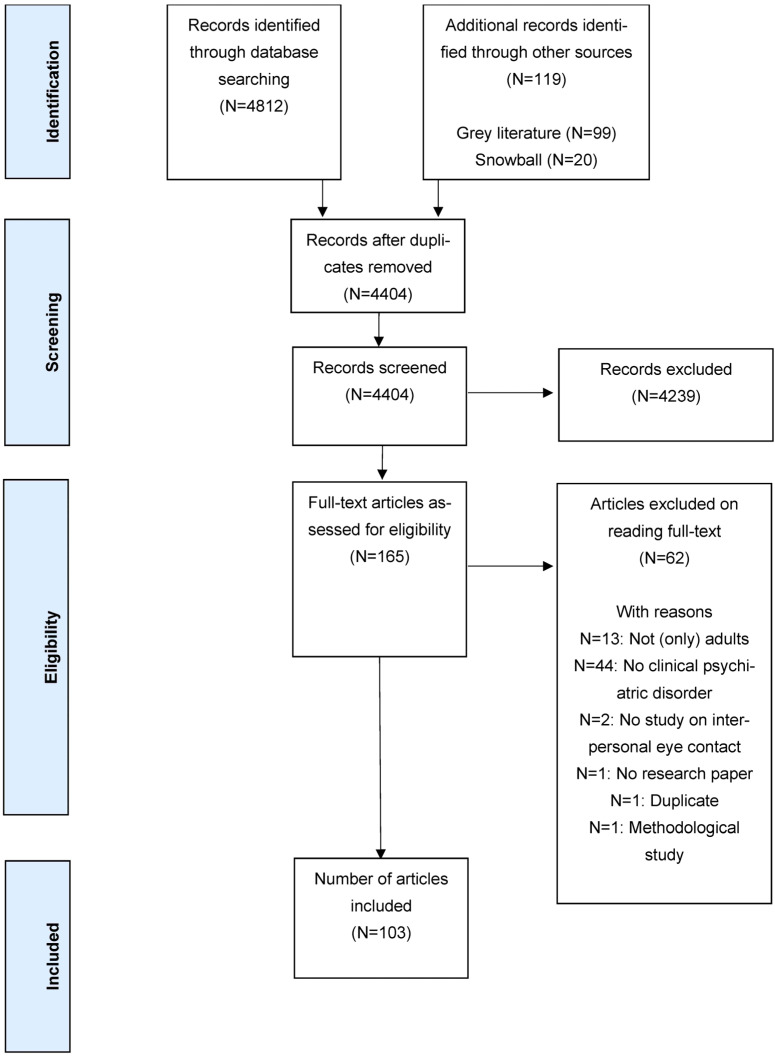
PRISMA flowchart for research question 2 of the scoping review process.

The databank, grey literature and snowball searches yielded a total of 4931 potential articles for research sub-question 2 (see Figure 2 for PRISMA flowchart). After duplicates were removed, 4404 articles were independently screened by two reviewers for title and abstract. After exclusion, 165 articles were assessed for eligibility. Finally, 103 articles were included in this research: 42 articles (with 42 sub-studies) focusing on presentations of eye contact and 62 articles (with 63 sub-studies) focusing on interpretations of eye contact. During this process, multiple consensus meetings occurred whereby Cohen’s Kappa values for inter-rater reliability [63] ranged between 0.66 (moderate) and 0.90 (strong).

### 3.2. Characteristics of Sources of Evidence

#### 3.2.1. Research Sub-Question 1

Regarding the study design for presentations of eye contact in social categorizations, 92 quasi-experimental studies, 9 RCTs, 2 qualitative research studies, 1 diagnostic test accuracy study and 1 textual evidence: narrative were included. A summary of countries where the studies were conducted is presented in Table 1, and social categorizations that were studied are provided in Table 2. Data were extracted according to article characteristics, as described in Section 2. Method. See Supplementary Materials, Table S5 for total data-extraction information, including references and the meanings of abbreviations.

For interpretations of eye contact in social categorizations, the study designs included 144 quasi-experimental studies, 17 textual evidence: narrative, 5 RCTs, 4 qualitative studies, 3 case reports, 2 diagnostic test accuracy studies, 1 systematic review and 1 textual evidence: expert opinion. The countries where the studies were conducted are summarized in Table 1 (multiple research projects were conducted in multiple countries). The social categorizations that were studied are presented in Table 2. Data on the same article characteristics were extracted as described for presentations of eye contact in social categorizations. For total data extraction information, including references and the meaning of abbreviations, see Supplementary Materials, Table S5.

#### 3.2.2. Research Sub-Question 2

Concerning the study design for presentations of eye contact in psychiatric disorders, 33 quasi-experimental studies, 3 systematic reviews, 2 RCTs, 2 textual evidence examples: expert opinion, 1 textual evidence example: narrative and 1 case report were included. A summary of countries where the studies were conducted is presented in Table 3. The psychiatric disorders that were studied are listed in Table 4. Data were extracted according to article characteristics as described in Section 2. Method. See Supplementary Materials, Table S6 for total data-extraction information, including references and the meaning of abbreviations.

For interpretations of eye contact in psychiatric disorders, the study designs included 52 quasi-experimental studies, 4 textual evidence examples: narrative, 2 RCTs, 2 diagnostic test accuracy studies, 1 case report and 1 systematic review. The countries where the studies were conducted are provided in Table 3, and the psychiatric disorders that were studied are presented in Table 4. Data on the same article characteristics were extracted as described for presentations of eye contact in psychiatric disorders. See Supplementary Materials, Table S6 for total data-extraction information, including references and the meaning of abbreviations.

### 3.3. Critical Appraisal within Sources of Evidence

#### 3.3.1. Research Sub-Question 1

Critical appraisal of all 105 articles on presentations of eye contact in social categorizations was performed according to the JBI criteria [59]. Overall, 1 RCT met all the JBI criteria. Critical appraisal according to the JBI criteria was also performed on the 177 articles with a focus on interpretations of eye contact in social categorizations. Of these, 18 textual evidence examples: narrative, 1 RCT and the systematic review met all the JBI criteria. For a critical appraisal of all studies on presentations and interpretations, see Supplementary Materials, Table S7.

#### 3.3.2. Research Sub-Question 2

Critical appraisal according to the JBI criteria was also performed for all 42 articles on presentations of eye contact in psychiatric disorders. Of these articles, 1 textual evidence example: narrative, 2 textual evidence examples: expert opinion, 1 systematic review and 1 RCT met all the JBI criteria. Of the 62 articles with a focus on interpretations of eye contact in social categorizations, critical appraisal according to the JBI criteria showed that 3 textual evidence examples: narrative, 1 systematic review, 1 RCT and 1 quasi-experimental study met all the JBI criteria. For a critical appraisal of all studies on presentations and interpretations, see Supplementary Materials, Table S8.

### 3.4. Results of Individual Sources of Evidence

For each source of evidence included, relevant outcomes were charted that relate to the review questions and objectives of both the presentation and interpretation of eye contact. Altogether, N = 386 articles were included with a total of N = 282 articles for research sub-question 1 (N = 105 articles on presentation and N = 177 articles on interpretation) and a total of N = 104 articles for research sub-question 2 (N = 42 articles on presentation and N = 62 articles on interpretation). Overall, N = 455 sub-studies were included with a total of N = 350 sub-studies for research sub-question 1 (N = 118 sub-studies on presentation and N = 232 sub-studies on interpretation) and a total of N = 105 sub-studies for research sub-question 2 (N = 42 sub-studies on presentation and N = 63 sub-studies on interpretation). Ultimately, N = 603 individual outcomes were included with a total of N = 481 outcomes for research sub-question 1 (N = 174 outcomes on presentation and N = 307 outcomes on interpretation) and a total of N = 122 outcomes for research sub-question 2 (N = 50 outcomes on presentation and N = 72 outcomes for interpretation). Due to the large number of sub-studies and outcomes, these are separately presented in appendices. For research sub-question 1, see Supplementary Materials, Table S9 for details on presentations and Supplementary Materials, Table S10 for details on interpretations. For research sub-question 2, see Supplementary Materials, Table S11 for details on presentations and Supplementary Materials, Table S12 for details on interpretations.

### 3.5. Synthesis of Results

#### 3.5.1. Research Sub-Question 1

All sub-studies with a focus on the presentation of eye contact in social categorizations were included. Data were thematically analyzed and clustered into nine different forms. For an overview of the number of outcomes categorized by theme and the number of articles mentioned, see Table 5. The results show that within the different themes, there is no gold standard for the presentation of eye contact. No clear correlation is found in variations between populations or variations between themes. Additionally, the results do not reveal why a specific variation is used in a particular population. Many studies do not describe the nationality, culture or religion of the population studied. When this was described, studies mainly focused on one specific characteristic, namely nationality, age, sex or psychiatric traits. Investigations of differences between populations mainly concern comparisons between Caucasia and Asia or males and females. Further, specific outcomes occur more often than others. For example, multiple studies show that the duration of eye contact appears to be linked to the experienced quality of contact between dyads [17,52,64,65]. A decreased amount of gaze is often found in psychiatric traits [66,67,68,69,70,71], and most articles on gaze avoidance focus on social anxiety [35,72,73,74,75,76,77]. Multiple studies describe the eye region as the facial region of preference in Asians while the mouth is preferred by Caucasians [78,79,80]. Similarly, males seem to prefer the mouth over the eyes, in contrast to females [81,82,83]. It is also suggested that eye contact functions as a signal for speaking and listening in conversations [49,84,85,86,87,88].

All sub-studies were included for research on the interpretation of eye contact in social categorizations. Data were thematically analyzed and clustered into 10 different forms of interpretations. See Table 6 for an overview of the number of outcomes categorized by theme and the number of articles mentioned. As with presentations of eye contact in social categorizations, it is shown that there are no clear correlations found between populations or variations between themes in the case of interpretations. The focus of studies appears to be mainly on one specific characteristic of populations. No study with a focus on a specific culture or religion is found, although several indicate that perceptions partly arise from cultures [89,90,91]. While no gold standard emerged from the results, some outcomes are found in multiple studies. Various research shows that gaze direction can have both a positive and negative influence on attributions to others, such as competences, personality traits, attractiveness, intelligence and liking (for example: [29,30,31,32,92,93,94,95,96,97]). Furthermore, several indications are found that a more mutual gaze is experienced more positively than a reduced gaze [26,27,98,99], although it is also described that extended gazing is experienced as threatening [44,100,101]. Most research into interpretations of eye contact in social categorizations has been conducted on the brain regions involved. These studies show an increased activity of the amygdala, gyri, sulci, working memory and social brain during eye contact (for example, [11,21,102,103,104,105,106,107,108,109]). There are indications that the activation of brain regions as a response to gaze is strongly modulated by the social meaning of a gaze cue and the belief that another person is making eye contact [110].

#### 3.5.2. Research Sub-Question 2

All sub-studies with a focus on the presentation of eye contact in psychiatric disorders were included. Data were thematically analyzed and clustered into six different forms of presentations. For an overview of the number of outcomes categorized by theme and the number of articles mentioned, see Table 7. The results show that each study focused on presentations of eye contact in a single specific disorder without assessing the correlation with other categorizations, such as age, nationality or culture, with the exception of a study by Riemer [111]. A large variety of psychiatric disorders are also found with variations within the diverse themes whereby the correlation between the different disorders is not evident. Furthermore, most studies focus on the amount of gaze and gaze avoidance. Far more results were found for decreased gaze than increased gaze. Decreased gaze is mainly seen in autism [112,113,114,115,116,117] and schizophrenia [118,119], whereas gaze avoidance is mainly shown in social anxiety disorder (for example: [41,120,121,122,123]. Fewer outcomes are also found for longer durations of eye contact than shorter durations. Shorter duration is found in persons with aphasia [124], PTSD [125], psychopathy [126] and social anxiety [127]. Additionally, it is notable that none of the studies investigated the participants’ reasons for presenting specific variations or investigated their experiences or visions regarding eye contact.

All sub-studies were included for research into the interpretation of eye contact in psychiatric disorders. Data were thematically analyzed and clustered into seven different forms of interpretations. See Table 8 for an overview of the number of outcomes categorized by theme and the number of articles mentioned. As with presentations of eye contact in psychiatric disorders, it is shown that, except in the research of MacDonald [128], each study focuses on a single specific disorder without assessing the correlation with other categorizations, such as age, nationality or culture. It is also indicated that the correlation between disorders sharing the same variation outcomes does not emerge. Moreover, no studies seem to describe the views or experiences of participants regarding eye contact. Notably, a wider cone of gaze is mainly seen in social anxiety disorder [3,35,129,130], although a wider cone of gaze is also found in schizophrenia [37,130], schizotypy [131] and bipolar disorder [39]. Furthermore, it appears that during the interpretation of others’ faces, the eye region is most preferred in social anxiety [132] and Huntington’s disease [133], and the mouth region most in schizophrenia [134] and autism [135]. Accuracy of emotional recognition from the eye region seems to be most impaired in autism [42,136,137,138,139]. It is also striking that atypical activation of brain regions is found in autism [140,141,142,143,144,145], social anxiety disorder [146], schizophrenia [147,148], PTSD [149,150] and fragile X syndrome [151].

## 4. Discussion

This review synthesized the relevant literature on variations in the usage and meaning of eye contact in social categorizations and psychiatric disorders worldwide and provided information for future research. Critical appraisal according to JBI criteria was applied to determine the quality of the included studies in this review. For both social categorizations and psychiatric disorders, the included studies met the majority of the JBI criteria. The lack of meeting all criteria was mainly in studies with quasi-experimental and RCT designs.

A novelty of this scoping review is that it provides an unprecedented broad overview of the existing knowledge about variations in appearances and interpretations of interpersonal eye contact in all possible populations worldwide. The results show that there are many variations in social categorizations and psychiatric disorders worldwide, with the vast majority of outcomes focusing on populations without psychiatric disorders. It remains unclear why populations use specific forms of eye contact or respond to them in a specific way and for what reason populations do or do not share variations in appearances or interpretations.

One possible reason is that existing research does not include sufficient factors when examining dyadic eye contact. Results from this scoping review show that variations are influenced by multiple characteristics, such as nationality, age, personality, sex and psychiatric traits. This finding indicates that effective insight into eye contact in populations requires that all influencing characteristics of these populations and between populations should be weighed. However, almost every study focuses on a singular characteristic. Additionally, there are some characteristics that may influence eye contact but which have received little attention in studies thus far. For example, of the total of 386 articles, only one study focuses on an African country [152] and one study on a South American country [49], which raises the question of to what extent eye contact is a point of attention in these continents. No research was found with a focus on religion, and very limited research was identified with a focus on cultures, despite previous research having shown that approaches to eye contact partly arise also from cultural contexts [89,90,91]. As with social categorizations, no final insight has yet been obtained into the cause, effect and management of eye contact in psychiatric disorders. A reason for this may be that research to date has mainly focused on autism, schizophrenia and social anxiety disorder, while aberrances have been described in many other disorders. Another reason could be that the views and experiences of participants regarding eye contact have not been inventoried, with the exception of one study by Trevisan et al. [44].

Because the studies found do not provide a clear image of how eye contact should be presented and interpreted worldwide, this could raise the question of whether there is such a thing as a “gold standard” for how eye contact should be managed. Although similarities are found within nationalities and characteristics such as gender in how eye contact is conducted, the totality of outcomes in this review suggests that a global gold standard does not exist. Rather, managing eye contact seems to arise from an individual combination of influencing characteristics that determine social meaning, which is consistent with previous research, such as that of Hamilton [110].

The fact that no gold standard has been found to date also makes it difficult to determine on what specific basis appearances and interpretations of eye contact must be seen as atypical in populations with, compared to without, psychiatric disorders. Following the previous observation that approaches to eye contact arise from social meaning, eye contact in psychiatric disorders may better be seen as a spectral deviation from social norms rather than a symptom of illness. From this perspective, people who experience inconvenience because of eye contact would benefit from future research that focuses more on the consequences of eye contact on their perceived quality of life.

### 4.1. Strengths and Limitations

One strength of this scoping review is that it provides, for the first time, a transparent and reproducible overview of existing research on presentations and interpretations of eye contact in all possible human populations worldwide. Additionally, the current scoping review shows that there is a large amount of variation in presentations and interpretations, not only between populations but also within populations. Another strength is that potential bias is reduced by the critical appraisal of study quality, the use of peer review during the selection process and the calculation of inter-rater reliability.

There are also some limitations that must be considered. For example, this scoping review assumes dyadic eye contact in the broadest sense of the term. However, the debate is still ongoing about what the precise definition of dyadic eye contact should be [4,5,6,153,154], so there may be differences of opinion about which studies should have been included. Another limitation is that although this scoping review analyzed many articles, important articles on presentations and interpretations of eye contact may have been missed. This could be due to the different views on and descriptions of eye contact in articles, because of which, search terms were not met. Articles were also excluded if the participants (partly) consisted of children, but these articles may have yielded meaningful data. Additionally, articles may have been missed because only Dutch and English articles were included, which could also explain why almost no studies were found from the African and South American continents in both social categorizations and psychiatric disorders. Another limitation is that in much research, only limited descriptions were given of the social categorizations of participants. Therefore, a less detailed oversight may have been given than desired. For example, out of necessity, the country in which the research was conducted was noted when the nationalities of participants were not clearly described.

### 4.2. Conclusions

An important conclusion of this scoping review is that there are many variations worldwide in how eye contact is presented and interpreted. This review identified 386 articles that contained 603 different outcomes on a wide range of populations, subdivided into social categorizations and psychiatric disorders. Overall, methodological quality was moderate to good. The prior literature has mainly examined presentations or interpretations of eye contact in specific populations. The diversity of these interpretations has been confirmed in this scoping review, but the current study also shows that there is significant variation between and within populations. Another conclusion is that aberrances of presentations and interpretations of eye contact are found in populations with and without psychiatric disorders. These findings also suggest that eye contact in psychiatric disorders could be a transdiagnostic factor that allows for a wide range of variation. Further, a gold standard for how eye contact should be presented and interpreted does not clearly emerge from the studies, so it is questionable to make general statements about abnormalities in eye contact. A shortcoming seems to be that virtually no research has been found that gives reasons for variations in presentations and interpretations of eye contact, how these variations are exactly linked to a specific population, or why multiple populations have these variations in common. One reason for this lack could be that, to date, very limited research has been conducted in which the views or experiences of participants regarding eye contact are inventoried. These described gaps could provide inspiration for future research.

## Figures and Tables

**Table 1:** Countries where studies for presentation and interpretation of eye contact in social categorizations were conducted.

Country	Presentation (N)	Interpretation (N)	Total Presentation and Interpretation (N)
USA	39	55	94
UK	29	41	70
Japan	3	13	16
Canada	2	12	14
Germany	4	10	14
Australia	8	5	13
China	3	10	13
France	1	10	11
The Netherlands	7	2	9
Finland	1	6	7
Switzerland	0	4	4
Italy	1	2	3
Belgium	0	3	3
Spain	0	3	3
Portugal	1	1	2
South Africa	1	0	1
Taiwan	1	0	1
Russia	0	1	1
Georgia	0	1	1
South Korea	0	1	1
Unspecified	4	2	6
Total	105	182	287

**Table 2:** Social categorizations that were studied for presentation and interpretation of eye contact.

Social Categorization	Presentation (N)	Interpretation (N)	Total Presentation and Interpretation (N)
People in Australia, Belgium, Canada, Finland, France, Germany, Italy, Portugal, Spain, Switzerland, The Netherlands, UK, USA	33	88	121
Males in Australia, Belgium, Canada, France, Germany, Italy, UK and USA	11	20	31
Chinese, Indians, Japanese, South Koreans, Taiwanese	6	13	19
Western Caucasians in Canada, UK and USA	8	8	16
Females in Australia, Canada, China, UK and USA	7	5	12
Brain damage in USA, Belgium, Japan, Switzerland and USA	2	7	9
Traits of anxiety in Australia, Canada, China, Japan, UK and USA	3	6	9
Traits of social anxiety in Germany, Portugal, South Africa, The Netherlands and USA	7	2	9
Traits of autism in Australia, China, Japan and The Netherlands	5	0	5
Love couples in Australia and USA	3	0	3
Traits of aggressiveness in USA	1	1	2
Traits of alexithymia in Canada	1	1	2
Traits of neuroticism in Finland and USA	2	0	2
Traits of psychopathy in male inmates in USA	2	0	2
Turner syndrome in UK	0	2	2
British born in China	1	0	1
Church worshippers in USA	0	1	1
Congenitally deaf in Japan	1	0	1
Doctors with burn-out symptoms in The Netherlands	1	0	1
FMR1 premutation in USA	1	0	1
Foreign language teachers in China	0	1	1
High-dominancy in USA	0	1	1
Low- and high-power receivers in USA	1	0	1
Native English speakers in The Netherlands	1	0	1
Non-Black students in Canada	0	1	1
Nursing students in Sweden	0	1	1
Parents of children with autism in UK	0	1	1
Patients at emergency departments in Australia	1	0	1
Relatives of adults with autism in UK	0	1	1
Sad persons in UK	1	0	1
Traits of affectiveness in postnatal mothers in UK	1	0	1
Traits of extraversion in UK	1	0	1
Traits of gelotophobia in Spain	0	1	1
Traits of hypomania in The Netherlands	1	0	1
Traits of psychopathy in Germany	1	0	1
Worldwide	1	0	1
Unspecified	1	16	17
Total	105	177	282

**Table 3:** Countries where studies for presentation and interpretation of eye contact in psychiatric disorders were conducted.

Country	Presentation (N)	Interpretation (N)	Total Presentation and Interpretation (N)
USA	12	16	29
Germany	6	7	13
UK	3	10	13
Australia	7	5	12
France	3	5	8
Canada	1	5	6
Switzerland	1	3	4
The Netherlands	3	0	3
Brazil	2	0	2
South Korea	2	0	2
Japan	0	2	2
Spain	0	2	2
Belgium	1	0	1
China	0	1	1
Denmark	0	1	1
Hungary	0	1	1
Italy	0	1	1
South Africa	0	1	1
Sweden	0	1	1
Unspecified	1	1	2
Total	42	62	105

**Table 4:** Psychiatric disorders that were studied for presentation and interpretation of eye contact.

Psychiatric Disorder	Presentation (N)	Interpretation (N)	Total Presentation and Interpretation (N)
Autism	12	19	31
Schizophrenia	4	14	18
Social anxiety disorder	12	6	18
Borderline personality disorder	2	3	5
Post-traumatic stress disorder	1	3	4
Psychiatric disorders	2	1	3
Bipolar disorder	0	3	3
Alzheimer’s disease	1	1	2
Depressive disorder	2	0	2
Frontotemporal dementia	1	1	2
Mental retardation	1	1	2
Eating disorders	0	2	2
Huntington’s disease	0	2	2
Aphasia	1	0	1
Alcohol use disorder	0	1	1
Klinefelter syndrome (47,XXY)	1	0	1
Mood disorders	1	0	1
Psychopathy	1	0	1
Schizotypy	0	1	1
Fragile X syndrome	0	1	1
Insomnia	0	1	1
Parkinson’s disease	0	1	1
Unspecified	0	1	1
Total	42	62	104

**Table 5:** Thematic clustering of outcomes and the number of articles mentioned on presentations of eye contact in social categorizations.

Research Sub-Question 1		
*Presentation of eye contact in social categorizations*		
**Theme**	**Articles (N)**	**Outcomes (N)**
(1) Frequency of gaze	11	14
• Increased frequency	4	5
• Decreased frequency	5	6
• Dependencies of frequency	2	3
(2) Duration of gaze	14	16
• Longer duration	9	10
• Shorter duration	4	5
• Dependencies of duration	1	1
(3) Amount of gaze	42	45
• Increased gaze	19	22
• Decreased gaze	17	17
• Dependencies of amount	6	6
(4) Gaze avoidance	13	14
• Gaze avoidance and anxiety	7	7
• Gaze avoidance and personality	2	3
• Other dependencies of avoidance	4	4
(5) Gaze direction in response to expressed emotion	8	11
• Direct gaze in response to emotion	5	5
• Averted gaze in response to emotion	1	2
• Shifting of attention	2	4
(6) Gaze direction preferences to facial regions	26	29
• Eye region	11	11
• Central face region	2	2
• Local and flanking region	1	2
• Nose and mouth region	8	10
• Region exploration	4	4
(7) Eye behavior and conversation	13	29
• Eye behavior and speaker-listening	4	12
• Eye behavior during questioning	6	12
• Other dependencies of eye behavior and conversation	3	5
(8) Gaze cueing	6	11
• Gaze cueing and emotional expression	3	6
• Gaze cueing and other dependencies	3	5
(9) Pupil dilation and constriction	4	5
Total	137	174

**Table 6:** Thematic clustering of outcomes and the number of articles mentioned on interpretations of eye contact in social categorizations.

Research Sub-Question 1		
*Interpretation of eye contact in social categorizations*		
**Theme**	**Articles (N)**	**Outcomes (N)**
(1) Influence of gaze direction of others on attribution	26	36
• Positive attributions	13	18
• Negative attributions	7	9
• Other	6	9
(2) Influence of gaze direction of others on emotion perception	13	20
• Positive attributions	3	4
• Negative attributions	4	6
• Intensity of attributions	2	5
• Other	4	5
(3) Influence of eye contact on experiences	25	40
• Positive emotions	11	18
• Negative emotions	5	7
• Physical sensations	5	7
• Experience of time	2	5
• Other	2	3
(4) Influence of eye contact on behavior	20	30
• Positive behavior	8	10
• Negative behavior	3	3
• Task behavior	6	9
• Influence of starring on behavior	3	8
(5) Accuracy of emotion recognition from the eye region	27	35
• Accuracy	11	15
• Inaccuracy	10	14
• Dependencies of accuracy	6	6
(6) Accuracy of gaze discrimination	36	48
• Accuracy	11	15
• Inaccuracy	12	15
• Dependencies of accuracy	13	18
(7) Gaze direction to facial regions during interpretation	10	13
• Eye region	3	4
• Mouth region	2	2
• Multiple regions	5	7
(8) Gaze direction and attention	8	8
(9) Eye contact and neural network	54	67
• Amygdala responses	14	18
• Gyrus responses	6	7
• Sulcus responses	6	7
• Fronto-parietal responses	3	6
• Social brain responses	5	5
• EEG latencies	7	8
• Other brain region responses	3	4
• Unspecified brain regions	10	12
(10) Eye contact and space	10	18
• Personal space	3	11
• Physical space	7	7
Total	229	307

**Table 7:** Thematic clustering of outcomes and the number of articles mentioned on presentations of eye contact in psychiatric disorders.

Research Sub-Question 2		
*Presentation of eye contact in psychiatric disorders*		
**Theme**	**Articles (N)**	**Outcomes (N)**
(1) Frequency of eye contact	5	5
• Increased frequency	1	1
• Decreased frequency	3	3
• Normal frequency	1	1
(2) Duration of eye contact	5	5
• Longer duration	1	1
• Shorter duration	4	4
(3) Amount of gaze	14	15
• Increased gaze	2	2
• Decreased gaze	10	11
• Dependencies of amount	2	2
(4) Gaze avoidance	15	15
• Gaze avoidance and anxiety	12	12
• Other dependencies of avoidance	3	3
(5) Gaze direction in response to expressed emotion	8	8
• Direct gaze in response to emotion	6	6
• Averted gaze in response to emotion	2	2
(6) Atypical gaze	2	2
Total	49	50

**Table 8:** Thematic clustering of outcomes and the number of articles mentioned on interpretations of eye contact in psychiatric disorders.

Research Sub-Question 2		
*Interpretation of eye contact in psychiatric disorders*		
**Theme**	**Articles (N)**	**Outcomes (N)**
(1) Influence of gaze direction of others on emotion perception	3	3
(2) Influence of eye contact on behavior	2	2
(3) Accuracy of emotion recognition from the eye region	15	15
• Impairments in autism	5	5
• Impairments in other disorders	10	10
(4) Accuracy of gaze discrimination	18	23
• Cone of gaze	8	11
• Other impairments	5	5
• Velocity of judgement	3	5
• No Impairments	2	2
(5) Gaze direction to facial regions during interpretation	9	9
• Eye region	3	3
• Mouth region	2	2
• Eye and mouth region	2	2
• Other facial regions	2	2
(6) Eye contact and neural network	15	16
• Amygdala responses	4	4
• Gyrus responses	2	2
• EEG latencies	2	3
• Other brain region responses	7	7
(7) Influence of gaze direction of others on attribution	3	4
Total	65	72

## Supplementary Materials

The following supporting information can be downloaded at: https://www.mdpi.com/article/10.3390/ijerph21081092/s1, Table S1: Search string research subquestion 1; Table S2: Search string research subquestion 2; Table S3: Template Data Extraction Form research subquestion 1; Table S4: Template Data Extraction Form research subquestion 2; Table S5: Data Extraction research subquestion 1; Table S6: Data Extraction research subquestion 2; Table S7: Critical Appraisal research subquestion 1; Table S8: Critical Appraisal research subquestion 2; Table S9: Outcomes research subquestion 1—Presentations; Table S10: Outcomes research subquestion 1—Interpretations; Table S11: Outcomes research subquestion 2—Presentations; Table S12: Outcomes research subquestion 2 Interpretations.
