# Supplementary material for: Variations in the Appearance and Interpretation of Interpersonal Eye Contact in Social Categorizations and Psychiatric Populations Worldwide: A Scoping Review with a Critical Appraisal of the Literature
Source: Int J Environ Res Public Health. 2024 Aug 18;21(8):1092. doi: 10.3390/ijerph21081092 (PMC11354482; doi:10.3390/ijerph21081092)
Supplement: Supplementary file 1 [file ijerph-21-01092-s001.zip › Table S1 Search string research subquestion 1.pdf]

Table S1: Search string Research subquestion 1

PubMed (Medline)

("Anthropology"[Mesh] OR anthropol*[tiab] OR cultur*[tiab] OR ethnol*[tiab] OR ethnic*[tiab] OR caucasian*[tiab] OR asian*[tiab] OR african*[tiab] OR american*[tiab] OR european*[tiab] OR mediterranian*[tiab] OR chinese*[tiab] OR japanese*[tiab] OR muslim*[tiab] OR arab*[tiab] OR islam*[tiab] OR middle-east*[tiab] OR latin*[tiab] OR hispanic*[tiab] OR eastern[tiab] OR western[tiab] OR multicultur*[tiab]) AND ("Eye Movements"[Mesh] OR eye-movements[tiab] OR eyemovements[tiab] OR eye-contact*[tiab] OR eyecontact*[tiab] OR gaze[tiab] OR gazes[tiab] OR gazing[tiab] OR eye-track*[tiab] OR eyetrack*[tiab] OR saccad*[tiab] OR facial-express*[tiab] OR face-read*[tiab] OR reading-face*[tiab] OR reading-facial*[tiab] OR face-processing[tiab] OR processing-face*[tiab] OR processing-facial*[tiab] OR face-scanning[tiab] OR scanning-face*[tiab] OR head-orientat*[tiab] OR visual-attention*[tiab] OR visual-fixation*[tiab] OR visual-processing[tiab] OR ("Nonverbal Communication"[Mesh] OR "Social Interaction"[Mesh] OR social-interact*[tiab] OR ((non-verbal*[tiab] OR nonverbal*[tiab]) AND communicat*[tiab]) AND ("Eye"[Mesh] OR "Face"[Mesh] OR eye[tiab] OR eyes[tiab] OR face[tiab] OR faces[tiab] OR facial[tiab]))) AND ("Perception"[Mesh] OR "Awareness"[Mesh] OR "Association"[Mesh] OR "Judgment"[Mesh] OR aware*[tiab] OR percepti*[tiab] OR mispercepti*[tiab] OR mis-percepti*[tiab] OR perceiv*[tiab] OR mis-perceiv*[tiab] OR recogni*[tiab] OR cognition*[tiab] OR reception*[tiab] OR interpretat*[tiab] OR encod*[tiab] OR misinterpretat*[tiab] OR mis-interpretat*[tiab] OR associat*[tiab] OR correlat*[tiab] OR judgment*[tiab] OR judging*[tiab] OR judge[tiab]) AND ("Expressed Emotion"[Mesh] OR "Emotions"[Mesh] OR emotion*[tiab] OR facial-affect*[tiab] OR affective-process*[tiab] OR affective-context*[tiab] OR happy[tiab] OR happiness[tiab] OR sad[tiab] OR sadness[tiab] OR angry[tiab] OR anger[tiab] OR fear[tiab] OR fright*[tiab] OR repuls*[tiab] OR disgust*[tiab] OR surprise*[tiab]) AND (English[lang] OR Dutch[lang]) NOT (("Child"[Mesh] OR "Pediatrics"[Mesh] OR "Infant"[Mesh] OR child*[tiab] OR pediatric*[tiab] OR paediatric*[tiab] OR boy[tiab] OR boys[tiab] OR girl*[tiab] OR infant*[tiab] OR newborn*[tiab] OR neonate*[tiab]) NOT ("Adult"[Mesh] OR adult*[tiab] OR man[tiab] OR men[tiab] OR woman[tiab] OR women[tiab]))
