# Supplementary material for: Variations in the Appearance and Interpretation of Interpersonal Eye Contact in Social Categorizations and Psychiatric Populations Worldwide: A Scoping Review with a Critical Appraisal of the Literature
Source: Int J Environ Res Public Health. 2024 Aug 18;21(8):1092. doi: 10.3390/ijerph21081092 (PMC11354482; doi:10.3390/ijerph21081092)
Supplement: Supplementary file 1 [file ijerph-21-01092-s001.zip › Table S10 Outcomes Q1 - Interpretations.pdf]

Table S10: Outcomes Research subquestion 1 – Interpretations of eye contact in social categorizations

Social categorization	Modality	Source	Substudy
	**Influence of gaze direction of others on attribution**		
	Positive attributions		
Belgium	The more gaze, the higher the ratings on activity/potency of others	Argyle et al., 1974	
Belgium	Ratings of liking/evaluation of others increase from zero eye contact to normal eye contact	Argyle et al., 1974	
USA	An increase in the amount of eye contact generated by a speaker in a live public-speaking situation enhances the listener's perception of the speaker's credibility	Beebe, 1974	
China	Gaze patterns of more but shorter glances produces impressions of higher activity and higher potency	Bond & Goodman, 1980	
USA	Attributions of power increases as the proportion of looking while speaking increases, and attributions of power decreases as the proportion of looking while listening increases	Dovidio et al., 1982	Study 1
USA	Attributions of power increases as the proportion of looking while speaking increases, and attributions of power decreases as the proportion of looking while listening increases	Dovidio et al., 1982	Study 2
Australia	Direct gaze leads to higher attractiveness ratings	Ewing et al., 2010	
UK	Making eye contact enhances the appeal of an attractive unfamiliar face, irrespective of gender	Kampe et al., 2001	
USA	The communication of respect and genuineness is positively and significantly related to greater eye contact	Kelly & True, 1980	
USA	Persons who look in long gazes are liked more than persons who look in short frequent gazes	Kendon & Cook, 1969	
Caucasians	When Caucasians recognize emotional states from the eyes, happiness is more attributed to females with a cap or a shawl than with a niqab	Kret & de Gelder, 2012	
Germany	Speakers' direct gaze promotes trust in truth-ambiguous statements	Kreysa et al., 2016	
Females, USA	Conversational tone and listener attitude ratings are higher with increased interpersonal gaze	Naiman & Breed, 1974	
Males, USA	The effect that as eye contact increases, an individual is judged more positively, is less pronounced when the individual is a male	Napieralski et al., 1995	
Females, USA	The effect that as eye contact increases, an individual is judged more positively, is more pronounced when the individual is a female	Napieralski et al., 1995	
Low and intermediate social competence, USA	Persons of low and intermediate social competence are positively influenced by attraction and eye contact	Scherwitz & Helmreich, 1973	Study 3
Males, USA	With negative verbal content, increasing eye contact leads to greater liking in males	Scherwitz & Helmreich, 1973	Study 1
USA	With impersonal evaluation, high eye contact leads to greater attraction	Scherwitz & Helmreich, 1973	Study 2
	Negative attributions		
UK	Heightened arousal reduces trust evaluations of trustworthy and attractive faces with averted gaze more than with direct gaze	Abbott et al., 2018	Study 2
Belgium	Ratings of liking/evaluation of others become lower for continuous eye contact	Argyle et al., 1974	
Caucasians	When Caucasians recognize emotional states from the eyes, fear and anger are more attributed to females with a niqab than with a cap or a shawl	Kret & de Gelder, 2012	
Males, USA	With positive verbal content, high eye contact leads to less liking in males	Scherwitz & Helmreich, 1973	Study 1
USA	With personal positive evaluation, the other is less liked when high eye contact is established	Scherwitz & Helmreich, 1973	Study 2
USA	Job applicants who show no eye contact is seen as less self-confident, in individuals with low and high social status	Tessler & Sushelsky, 1978	
USA	Longer durations of eye contact and fewer eye shifts are more likely to be rated as having a higher intelligence	Wheeler et al., 1979	
UK	Persons rate faces with averted, compared to direct, gaze as less trustworthy	Wyland & Forgas, 2010	
UK	Persons in a positive mood rate faces with averted gaze as less friendly than faces with direct gaze, persons in a negative mood do not	Wyland & Forgas, 2010	
	Other		
USA	Eye contact affects the interviewers' evaluations of the applicants, and those evaluations are related to the decision to hire	Amalfitano et al., 1977	
Portugal	For romantic attraction in both males and females, an important feature is the partner's eye color and size, but even more to the eyes being expressive and friendly	Karandashev et al., 2016	
Georgia	For romantic attraction in both males and females, an important feature is the partner's eye color and size, but even more to the eyes being expressive and friendly	Karandashev et al., 2016	
Russia	For romantic attraction in both males and females, an important feature is the partner's eye color and size, but even more to the eyes being expressive and friendly	Karandashev et al., 2016	
France	For romantic attraction in both males and females, an important feature is the partner's eye color and size, but even more to the eyes being expressive and friendly	Karandashev et al., 2016	
Not applicable	Gaze of others functions to provide information about liking and attraction, attentiveness, competence, social skills and mental health	Kleinke, 1986	
UK	Gazing does serve primarily as means of collecting information rather than expressing interpersonal attitudes	Rutter & Stephenson, 1979	
High social competence, USA	Persons high on social competence are less effected by attractiveness and not effected by eye contact	Scherwitz & Helmreich, 1973	Study 3
Not applicable	Persons in different cultures share the common sense by using the same body language such as gazing and eye management, but can indicate different meanings	Zhi-Peng, 2014	
	**Influence of gaze direction of others on emotion perception**		
	Positive attributions		
Finland	Individuals from Finland perceive another’s face less as being angrier, unapproachable, and unpleasant when making eye contact as compared to individuals from Japan	Akechi et al., 2013	
Not applicable	Gaze functions to express intimacy	Kleinke, 1986	
Asia	A direct gaze relative to an averted gaze increases the perceived likelihood of the other being perceived as having a joyful disposition	Liang et al., 2021	Study 1
Asia	A direct gaze relative to an averted gaze increases the perceived likelihood of the other being perceived as having a joyful disposition	Liang et al., 2021	Study 2
	Negative attributions		
Students, USA	When displaying direct gaze, ambiguous facial blends are given more anger than fear labels and also anger as more intensely rated than fear	Adams & Kleck, 2005	Study 2
Students, USA	When displaying averted gaze, facial blends are given more fear than anger labels and also fear as more intensely rated than anger	Adams & Kleck, 2005	Study 2
UK	Not sad, happy or angry faces but only fearful faces shows an effect of eye gaze, with more fearful ratings when gaze is direct than averted	Bindemann et al., 2008	Study 4

Japan	Individuals from Japan perceive another's face as being angrier, unapproachable, and unpleasant when making eye contact as compared to individuals from Finland	Akechi et al., 2013	
Asia	An averted gaze relative to a direct gaze is more often associated with an angry and fearful disposition	Liang et al., 2021	Study 1
Asia	An averted gaze relative to a direct gaze is more often associated with an angry and fearful disposition	Liang et al., 2021	Study 2
	Intensity of attributions		
Students, USA	Direct gaze enhances the perceived intensity of pure expressions of anger and joy, whereas averted gaze enhances the perceived intensity of pure expressions of fear and sadness	Adams & Kleck, 2005	Study 3
China	The perceived intensity of an emotion expressed by ethnic in-group members is independent of gaze direction	Krämer et al., 2013	
China	When directly gazed at, emotions are perceived as more pronounced in Germans than in ethnic in-group members	Krämer et al., 2013	
Germany	The perceived intensity of an emotion expressed by ethnic in-group members is independent of gaze direction	Krämer et al., 2013	
Germany	When directly gazed at, emotions are perceived as more pronounced in Chinese than in ethnic in-group members	Krämer et al., 2013	
	Other		
Students, USA	Gaze direction systematically influences the perceived emotion disposition conveyed by neutral faces	Adams & Kleck, 2005	Study 1
Students, USA	Gaze direction influences the processing of facial anger and fear displays, in terms of both processing speed and perceptual interpretation	Adams & Kleck, 2003	Study 1
Students, USA	Gaze direction influences the processing of facial joy and sadness displays, in terms of both processing speed and perceptual interpretation	Adams & Kleck, 2003	Study 2
Canada	Specific gaze directions functionally overlap with emotion and attention discrimination, precursors to more elaborated theory of mind processes	McCrackin & Itier, 2019	
UK	Detection of emotional faces is modulated by the direction of eye gaze	Milders et al., 2011	
	**Influence of eye contact on experiences**		
	Positive emotions		
USA	Higher amounts of gaze is interpreted as more intimate, more immediacy and involvement, and more composure, informality and nonarousal	Burgoon et al., 1985	
Finland	Direct gaze automatically elicit more positive affective reactions than closed eyes	Chen et al., 2017	Study 1
Finland	Direct gaze automatically elicit more positive affective reactions than closed eyes and direct gaze is evaluated to be more positive than averted gaze, independent of time of display	Chen et al., 2017	Study 2
Females, USA	In contrast to males, direct gaze appears to promote intimacy between females, while gaze avoidance has the opposite effect	Ellsworth & Ross, 1975	
USA	Eye contact creates higher feelings of affection towards the interaction partner	Kellerman et al., 1989	Study 1
USA	Eye contact creates higher feelings of liking and loving between interaction partners	Kellerman et al., 1989	Study 2
UK	For faces with neutral expressions, persons clearly prefer eyes looking towards them compared to eyes gazing to the right or left	Lawson, 2015	Study 1
UK	For faces with neutral expressions, persons clearly prefer eyes looking towards them compared to eyes gazing to the right or left with faces turned to the side	Lawson, 2015	Study 2
UK	For faces with neutral expressions, persons clearly prefer eyes looking towards them compared to eyes gazing to the right or left with faces turned upside-down	Lawson, 2015	Study 3
UK	For faces with angry expressions, persons clearly prefer eyes looking towards them compared to eyes gazing to the right or left	Lawson, 2015	Study 4
UK	For faces with angry expressions, persons clearly prefer eyes looking towards them compared to eyes gazing to the right or left	Lawson, 2015	Study 5
USA	Eye contact has an important role in establishing meaningful rapport	MacDonald, 2009	
USA	Eye contact has an important role in provisioning hope and embodied empathy	MacDonald, 2009	
Agressiveness traits, USA	Aggressive persons are far less deterred when stared at, in comparison to unaggressive persons	Moore & Gilliland, 1921	Study 2
USA	Direct gaze is rated as more pleasant than averted gaze	Schmitz et al., 2012	
Nursing students, Sweden	Nurses feel empathy to patients and their relatives when they experience eye contact	Söderberg et al., 2017	
China	Subliminal eye emotion affects a viewer's empathy for pain, whereby subliminal sad eyes have a greater impact on empathy for pain than subliminal fearful eyes	Song et al., 2019	
UK	Eye contact of radiographers, among other elements, is a key aspect of communication as a fundament for giving compassionate patient-centred care	Taylor et al., 2021	
	Negative emotions		
Males, USA	In contrast to females, direct gaze appears to promote reticence between males, while gaze avoidance has the opposite effect	Ellsworth & Ross, 1975	
Not applicable	Direct contact is one of many classes of potential threat	Skuse, 2006	
USA	Receivers of stares rate themselves more angry, unfriendly, unpleasant and embarrassed, while the starer is rated as more tense, angry, embarrassed, passive and less intelligent	Strom & Buck, 1979	
Males, USA	Being stared at by a stranger in absence of verbal communication is seen as a threat in which the person who cannot stand up to the other' stare, feels been beaten of dominated	Thayer, 1969	
Autism traits, Canada	Persons with self-declared autism experience feelings of being invaded while making eye contact	Trevisan et al., 2017	
Autism traits, Canada	Persons with self-declared autism experience difficulties understanding social nuances while making eye contact	Trevisan et al., 2017	
Autism traits, Canada	Persons with self-declared autism experience difficulties receiving and sending nonverbal information while making eye contact	Trevisan et al., 2017	
	Physical sensations		
Males, USA	Only in the first five seconds of males seeing smiling faces produces arousal, there is no effect of seeing direct gazing on arousal	Martin & Gardner, 1979	
UK	Both frequency and amplitude of galvanic skin response are greater during eye contact	Nichols & Champness, 1971	
UK	Galvanic skin responses are greater when gazes are reciprocated than when unreciprocated	Nichols & Champness, 1971	
USA	Being stared at creates more arousal in the presence of an opposite-sex interaction partner	Strom & Buck, 1979	
Autism traits, Canada	Persons with self-declared autism experience sensory overload while making eye contact	Trevisan et al., 2017	
Autism traits, Canada	Persons with self-declared autism experience adverse physiological reactions while making eye contact	Trevisan et al., 2017	
Social anxiety traits, Germany	Persons with social anxiety respond to direct gaze with more pronounced cardiac acceleration	Wieser et al., 2009	
	Experience of time		
Switzerland	Time perception is underestimated during eye contact	Burra & Kerzel, 2021	Study 1

Switzerland	Time perception is underestimated during eye contact	Burra & Kerzel, 2021	Study 2
Switzerland	Time perception is underestimated during eye contact	Burra & Kerzel, 2021	Study 3
Switzerland	Time perception is underestimated during eye contact	Burra & Kerzel, 2021	Study 4
Females, USA	During eye contact time is experienced as passing more slowly	Thayer & Schiff, 1975	
	Other		
Finland	Seeing a live face with a direct gaze is processed more intensely, as the direct gaze is capable of intensifying the feeling of being the target of the other's interest and intentions	Pönkänen et al., 2011	
Males, USA	Males weigh eye contact cues of duration and reciprocity less heavily than do females	Thayer & Schiff, 1974	
Feales, USA	Females weigh eye contact cues of duration and reciprocity more heavily than do males	Thayer & Schiff, 1974	
	**Influence of eye contact on behavior**		
	Positive behavior		
UK	Significantly more money is donated to charity during door-to-door collections when the collector makes eye contact. Style of dress or locality has no effect on the amount of donations	Bull & Gibson-Robinson, 1981	
USA	Students participate more in class when teacher makes eye contact with them	Caprioni et al., 1977	
UK	Watching eyes causes persons to behave more prosocially and causes increased donations to charity	Fathi et al., 2014	
Finland	Another individual's direct gaze reduces lying	Hietanen et al., 2018	
Males, USA	Males are most favorable toward females when they think they give the females low levels of gaze	Kleinke et al., 1973	
Females, USA	Females gives males the most favorable ratings when they are ostensibly gazed at the males	Kleinke et al., 1973	
Males, USA	Gaze and touch serves additive functions of increasing compliance to unambiguous requests in males	Kleinke, 1977	
Not applicable	Gaze functions to regulate interaction	Kleinke, 1986	
Not applicable	Gaze functions to exercise social control	Kleinke, 1986	
Females, USA	When an individual seeks help with direct gaze, helping is increased when both the victim and the person are female	Valentine & Ehrlichman, 1979	
	Negative behavior		
UK	Persons give significantly more shocks to others with direct gaze than averted gaze	Ellsworth et al., 1973	
Females, USA	Females tend to use reciprocal eye contact to signal dominance in response to direct gaze	Fromme & Beam, 1974	
Males, USA	When an individual seeks help with direct gaze, helping is decreased when both the victim and the person are male	Valentine & Ehrlichman, 1979	
	Task behavior		
France	The Stroop effect is stronger in the context of direct gaze than in the context of closed eyes, and independent of head orientation	Conty et al., 2010	Study 1
France	The Stroop effect is not influenced in the context of averted gaze or closed eyes, and independent of head orientation	Conty et al., 2010	Study 3
Foreign language teachers, China	Eye contact between foreign English teachers and students can eliminate communication barriers between them and improve student learning efficiency	Dai, 2021	
Not applicable	Gaze functions to facilitate service and task goals	Kleinke, 1986	
UK	Reaction times to the direction in which the head is oriented are significantly slower when the eyes are gazing in the opposite direction to the head	Langton, 2000	Study 1
UK	Direct eye gaze does not prompt a general enhancement in task performance, instead, the effects of gaze direction are confined to the accessibility of categorical knowledge	Macrae et al., 2002	Study 2
UK	When processing faces, gender categorization times are fastest when faces show a direct gaze	Macrae et al., 2002	Study 1
Japan	Persons are slower to respond to a task that follows faces with direct gaze	Senju & Hasegawa, 2005	Study 1
Japan	Persons spend equally time to respond to a task that follows faces with direct gaze as with averted gaze	Senju & Hasegawa, 2005	Study 2
	Influence of staring on behavior		
Church worshippers, USA	Persons invading pews in churches while staring increases the likelihood of a worshipper moving over or return gaze, worshippers rarely make both responses	Campbell & Lancioni, 1979	
USA	Pedestrians do not walk faster but slower, when being stared at on the street	Elman et al., 1977	Study 1
USA	When confronted with a long stare at an elevator persons walk significantly faster, when confronted with a short stare they walk significantly slower	Elman et al., 1977	Study 2
Caucasian automobile drivers, USA	Persons who are stared at in traffic, cross the intersection significantly faster than persons who are not stared at	Ellsworth et al., 1972	Study 1
Caucasian automobile drivers, USA	Persons who are stared at in traffic, cross the intersection significantly faster than persons who are not stared at	Ellsworth et al., 1972	Study 2
Caucasian automobile drivers, USA	Persons who are stared at in traffic, cross the intersection significantly faster than persons who are not stared at	Ellsworth et al., 1972	Study 3
Pedestrians, USA	Persons who are stared at in traffic, cross the intersection significantly faster than persons who are not stared at	Ellsworth et al., 1972	Study 4
USA	Persons who are stared at in traffic, cross the intersection significantly faster than persons who are not stared at	Ellsworth et al., 1972	Study 5
	**Accuracy of emotion recognition from the eye region**		
	Accuracy		
Females, Belgium	Females show superiority in facial emotion recognition from the eye region	Alaerts et al., 2011	Study 2
UK	Accuracy of facial emotion recognition from the eye region is enhanced with working memory	Eddy & Hansen, 2020	
Schizotypal personality traits, UK	Accuracy of facial emotion recognition from the eye region is enhanced in persons with schizotypal personality traits	Eddy & Hansen, 2020	
UK	Accuracy of facial emotion recognition from the eye region is enhanced in persons with empathy	Eddy & Hansen, 2020	
UK	Accuracy of facial emotion recognition from the eye region is enhanced in persons liking dogs	Eddy & Hansen, 2020	
Non-Black students, Canada	White persons distinguishes more between true and false smiles on White than Black targets except for when focus is on the eyes	Friesen et al., 2019	Study 6
Females, Italy	Females perform better in recognizing emotional states from the eye region, in comparison to males	Guariglia et al., 2015	Study 1
Artists, Italy	Artists perform better in recognizing emotional states from the eye region, in comparison to non-artists	Guariglia et al., 2015	Study 2

Females, Germany	Females perform better in recognizing emotional states from the eye region, in comparison to males	Isernia et al., 2020	
Not applicable	Females show superiority in 'Reading the Mind in the Eyes Test'	Kirkland et al., 2013	
China	Chinese recognize sad expressions in Asian faces with direct gaze better than in White ones, and accuracy is higher for White neutral, happy, fearful, and disgusted expressions	Ma et al., 2022	
China	When processing expressions and social intensions from the eyes, Chinese are particularly accurate and sensitive the more they self-report greater collectivistic and lower individualistic values	Mai et al., 2021	
Canada	Persons are able to recognize very subtle differences of mental states when judging the eye region, in very short times	Schmidtmann et al., 2020	
USA	US Americans are more accurate and have a more contrasting strategy than Chinese at recognizing emotional expressions on faces with direct gaze	Stanley et al., 2013	
Females, Australia	Females are significantly faster than males in correctly identifying facial expressions	Vassallo et al., 2009	
	Inaccuracy		
Amygdala damage, USA	Persons with amygdala damage are impaired when asked to recognize social emotions from the eye region	Adolphs et al., 2002	
Males, Belgium	Males show less accuracy in facial emotion recognition from the eye region	Alaerts et al., 2011	Study 2
Males, UK	Inflammation in males leads to a significant decrease of recognizing social emotions from the eye region of the face	Balter et al., 2018	
UK	Response time to facial emotional expressions are consistently slower to faces with averted eye gaze than direct gaze, both for happy and sad emotional expressions	Bindemann et al., 2008	Study 1
UK	Persons respond slower to faces with horizontally averted gaze relative to direct gaze, independent of the displayed facial expression	Bindemann et al., 2008	Study 2
UK	Response time to happy, sad, angry and fearful faces with averted gaze are consistently slower than with direct gaze	Bindemann et al., 2008	Study 3
UK	The perception of angry expressions is weakened by averted eye gaze, whereas the perception of fear is actually enhanced	Bindemann et al., 2008	Study 5
UK	Response time to happy, sad, angry and fearful faces with averted gaze are consistently slower than with direct gaze	Bindemann et al., 2008	Study 6
Alexithymia traits, Canada	Persons with alexithymia show lower accuracy of emotion recognition from the eyes	Fujiwara, 2018	
Males, Italy	Males perform poorer in recognizing emotional states from the eye region, in comparison to females	Guariglia et al., 2015	Study 1
Males, Germany	Males perform poorer in recognizing emotional states from the eye region, in comparison to females	Isernia et al., 2020	
Females with Turner syndrome, UK	The processing of displays of the eye region affording social and affective information is specifically affected in females with Turner syndrome	Lawrence et al., 2003	
China	Chinese are less accurate and have a less contrasting strategy than US Americans at recognizing emotional expressions on faces with direct gaze	Stanley et al., 2013	
Males, Australia	Males are significantly slower than females in correctly identifying facial expressions	Vassallo et al., 2009	
	Dependencies of accuracy		
Not applicable	The study of how other persons' eyes is processed encompasses several realms of psychological, sociological, anthropological, clinical, and neuroscientific investigation	Frischen et al., 2007	
Not applicable	Emotion perceptions seems actively constructed by perceivers to fit the social and physical constraints of their cultural worlds	Gendron, 2017	
Not applicable	Culture affects by means of experiential shaping and social categorization the way in which information on gaze is collected and perceived	Hadders-Algra, 2022	
China	The typical Eastern decoding process to identify and interpret others' facial expressions and social intentions is with use of the eyes as diagnostic cues	Mai et al., 2021	
Spain	Accuracy of recognizing emotional states from the eye region is associated with complex emotional intelligence abilities	Megías-Robles et al., 2020	
Western	Perceived eye contact by Westerners can interact with facial processing during gender judgements and recognition memory, even when gaze direction is task-irrelevant	Vuilleumier et al., 2005	
	**Accuracy of gaze discrimination**		
	Accuracy		
STG lesion, Female, Japan	Right superior temporal gyrus (STG) lesion results in much more precision in detecting mutual gaze where the pursuit begins at extreme right eyesight	Akiyama et al., 2006	Study 2
Spain	Discrimination of a gaze is enhanced when persons can interpret it as looking directly at them even when that implies a reverse congruency effect	Cañadas & Lupiáñez, 2012	Study 1
Caucasians	In Caucasians, sensitivity to direct gaze is better for own-race than Asian-race faces	Collova et al., 2017	Study 1
Asians	In Asians, sensitivity to direct gaze is better for own-race than Caucasian-race faces	Collova et al., 2017	Study 1
Caucasians	In Caucasians, sensitivity to direct gaze is better for own-race than Asian-race faces, and greater for upright than inverted- faces	Collova et al., 2017	Study 2
Asians	In Asians, sensitivity to direct gaze is better for own-race than Caucasian-race faces, and greater for upright than inverted- faces	Collova et al., 2017	Study 2
Anxiety traits, UK	When orienting to the direction of eye gaze, high trait-anxious persons show an enhanced orienting to the eye gaze of faces with fearful expressions relative to other expressions	Fox et al., 2007	
Switzerland	There is no evidence for a stare-in-the-crowd effect	Framorando et al., 2016	Study 4
Western Caucasian	Western Caucasians and East Asians are evenly accurate in judging gaze direction of others	Jack et al., 2012	Study 2
East Asia	Western Caucasians and East Asians are evenly accurate in judging gaze direction of others	Jack et al., 2012	Study 2
UK	Judgements of gaze direction remain accurate for highly 'unusual' red-and-green coloured eyes, when the iris remains darker than the surrounding sclera	Ricciardelli et al., 2008	Study 3
UK	Persons perform significantly more accurate for judgements of direct gaze based on both eyes compared to when the judgements are based on just one eye	Ricciardelli et al., 2002	Study 1
Young adults, UK	Young adults show a less conservative bias than older adults in distinguishing others' direct and averted gaze	Slessor et al., 2008	Study 1
Gelotophobia traits, Spain	Persons with gelotophobia do not exhibit any problem with identifying others' emotions and affective features by faces with direct and averted gaze	Torres-Marin et al., 2017	Study 2
Finland	Finnish have a smaller bias toward judging slightly averted gazes as directed at them when judging Finnish rather than Japanese faces	Uono & Hietanen, 2015	
	Inaccuracy		
STG lesion, Female, Japan	Right superior temporal gyrus (STG) lesion results in both gaze processing deficit and concurrent aberrant gaze behavior of the victim self	Akiyama et al., 2006a	Study 1
STG lesion, Female, Japan	Right superior temporal gyrus (STG) lesion results in perceiving left gaze as straight and to a lesser degree, straight gaze as right	Akiyama et al., 2006a	Study 1
STG lesion, Female, Japan	Right superior temporal gyrus (STG) lesion results in perceiving left gaze as straight and to a lesser degree, straight gaze as right	Akiyama et al., 2006a	Study 3
STG lesion, Female, Japan	A circumscribed lesion to the right STG impairs the ability to utilize biological directional information such as gaze, but leaves non-biological counterpart intact	Akiyama et al., 2006b	
Females with Turner syndrome, UK	In females with Turner syndrome both the detection of whether gaze is directed at the person, as the detection of gaze cuing is impaired	Elgar et al., 2002	
Switzerland	Persons are faster at discriminating the position of the eyes of straight than averted gazes in a crowd	Framorando et al., 2016	Study 2
Australia	Persons have a prior expectation that other persons' gaze is directed toward them, especially when there is high uncertainty, such as at night or when the other person is wearing sunglasses	Mareschal et al., 2013	Study 2
UK	Black sclera and white irises impairs gaze perception to a much wider extent than white sclera and black irises, except for direct gaze in a straight face	Ricciardelli et al., 2008	Study 1
UK	Gaze judgements are worse for negative than positive eyes, regardless of the polarity of the surrounding face	Ricciardelli et al., 2008	Study 2

USA	Stress increases the feeling of being looked at	Rimmele & Lobmaier, 2012	
Social anxiety traits, Germany	There is a relationship between self-reported social anxiety and stronger self-directed perception of others' gaze directions, particularly for negative (angry, fearful) and neutral expressions	Schulze et al., 2013	
Older adults, UK	Older adults show a more conservative bias than younger adults in distinguishing others' direct and averted gaze	Slessor et al., 2008	Study 1
Gelotophobia traits, Spain	Persons with gelotophobia are less accurate at discriminating direct and averted gaze	Torres-Marin et al., 2017	Study 1
Japan	When judging slightly averted gazes as directed at them, Japanese show the same bias for faces of Japanese and for Finnish	Uono & Hietanen, 2015	
Male, frontal-lobe damage, USA	Individual with frontal-lobe damage exhibits a general impairment in orienting attention endogenously, and this impairment includes orienting from gaze cues	Vecera & Rizzo, 2006	
	Dependencies of accuracy		
Japan	When searching for oddly directed gaze, the primary determinant of search efficiency is not the local-feature of the location of the pupil in the eye, but the perceived direction of the gaze	Doi & Ueda, 2007	
Switzerland	The occurrence of the stare-in-the crowd effect is limited to conditions where the gaze stimulus is part of the target face	Framorando et al., 2016	Study 3
Canada	When interpreting gaze direction of others, people use covert, reflexive orienting to peripheral locations in response to uniformative gaze shifts	Friesen & Kingstone, 1998	
Germany	Perceived gaze shifted toward the direction in which the head points, especially when the viewing distance is small and only one eye is visible	Gamer & Hecht, 2007	Study 1
Germany	Covering one of the eyes of others produces a roughly symmetric effect on the judged direction of the gaze cone	Gamer & Hecht, 2007	Study 2
Germany	Social stimuli entirely irrelevant to gaze discrimination has no influence on the observer's gaze direction estimates	Gamer & Hecht, 2007	Study 3
Germany	The head rotation of a gazing person affects the direction of the gaze cone by exerting an attraction	Gamer & Hecht, 2007	Study 4
Anxiety traits, Japan	The perceptual volume is much larger than the actual volume of eye-contact, and the subjective judgment of eye-contact elicits greater pupil dilation in perceivers	Honma, 2013	
Anxiety traits, China	Persons with anxiety traits have a wider cone of direct gaze for angry facial expressions of others, and a narrower cone of direct gaze for fearful expressions	Hu et al., 2017	
UK	Classification of eye gaze direction is influenced by the irrelevant orientation of the head	Langton, 2000	Study 2
USA	Faces with direct gaze are better memorized than with averted gaze	Mason et al., 2004	Study 1
USA	Faces with direct gaze are better memorized than with averted gaze	Mason et al., 2004	Study 2
UK	Gaze direction judgements are uninfluenced by contrast polarity of heads, irises and sclera	Ricciardelli et al., 2008	Study 4
Italy	Gaze judgements are not solely driven by the eye region but are also affected by part of the head and face	Ricciardelli et al., 2008	Study 3
Males, USA	The acquaintance of a subject and looker as well as the depth of gaze affects male subjects' judgement of a female's looking behaviour	Teske, 1988	
Unspecified, USA	Gaze cues direct attention in a voluntary, not a reflexive, manner	Vecera & Rizzo, 2006	
Relatives of adults with autism, UK	Relatives of individuals with autism show a similar sensitivity to direct compared with averted gaze direction	Wallace et al., 2010	
USA	Sclera color enhances the perception of gaze direction	Yorzinski & Miller, 2020	Study 2
	**Gaze direction to facial regions during interpretation**		
	Eye region		
UK	For complex mental states, seeing the eyes alone produce significantly better performance than seeing the mouth alone, and is as informative as the whole face	Baron-Cohen et al., 1997	Study 1
UK	For complex mental states, seeing the eyes alone produce significantly better performance than seeing the mouth alone, and is as informative as the whole face	Baron-Cohen et al., 1997	Study 2
Australia	In detecting emotions from faces, like fear, disgust and surprise, persons fixate more to the eyes than to mouth and nose	Vaidya et al., 2014	
Japan	Japanese focus more strongly on the eyes than the mouth when interpreting other's emotions, in contrast to US Americans	Yuki et al., 2007	Study 2
	Mouth region		
Caucasian	Mouths, more than eyes, is the most important cue for discriminating between static and dynamic facial expressions	Blais et al., 2012	
USA	US Americans tend to interpret emotions based on the position of the mouth, in contrast to Japanese	Yuki et al., 2007	Study 2
	Multiple regions		
UK	For emotion recognition from the face, the whole face is more informative than either the eyes or the mouth	Baron-Cohen et al., 1997	Study 1
UK	For emotion recognition from the face, the whole face is more informative than either the eyes or the mouth	Baron-Cohen et al., 1997	Study 2
Brain damage, prosopagnosia, female, Belgium	Persons with brain damage and prosopagnosia do not use optimal eye information to identify familiar faces, but instead the lower part of the face, including the mouth and the external contours	Caldara et al., 2005	
South-Korea	In South Koreans, fear is most recognized from the eyes, happiness, surprise, sadness, disgust and anger most recognized from the mouth	Kim et al., 2022	
UK	Information from both gaze and head orientation is extracted from the visual images even when participants are attempting to ignore these attention signals	Langton, 2000	Study 3
Males, Germany	Males reading emotions from the eyes and the face, trusting configural information	Pavlova et al., 2022	
Females, Germany	Females reading emotions from eyes, faces and bodies, trusting merely dynamic cues	Pavlova et al., 2022	
	**Gaze direction and attention**		
Unilateral amygdala lesions, Japan	Persons with unilateral amygdala lesions show a robust deficit in attentional orienting triggered by gaze direction	Akiyama et al., 2007	
Spain	Faces exert a powerful effect on capturing attention, especially when they display a direct gaze	Cañadas & Lupiáñez, 2012	Study 2
Switzerland	Irrespective of gaze direction, opened eyes are salient in the context of closed eyes due to their physical characteristics	Framorando et al., 2016	Study 1
Canada	Gaze direction triggers a reflexive shift of spatial attention	Friesen et al., 2005	
Canada	Eyes always attract attention but gaze orienting is task-dependent	Itier et al., 2007	
Not applicable	Although the eyes are an important cue for visual attention, other cues such as head orientation and pointing gestures contributes to the computation of another's direction of attention	Langton et al., 2000	
Anxiety traits, UK	Attention is more likely to be guided by the direction of fearful than neutral gaze, but only in persons with anxiety traits	Mathews et al., 2003	
White students, USA	White persons show biased attention toward Black faces relative to White faces	Trawalter et al., 2008	Study 1
	**Eye contact and neural network**		

	Amygdala responses		
Japan	Greater neural responsivity to averted -versus direct- gaze fear in regions related to face and emotion processing, when posed on same-culture faces in Japanese	Adams et al., 2010	
US Caucasian	Greater neural responsivity to averted -versus direct- gaze fear in regions related to face and emotion processing, when posed on same-culture faces in US Caucasians	Adams et al., 2010	
US Caucasian	US Caucasians show significantly greater activation in the right amygdala to averted gaze fear when viewing same-culture faces	Adams et al., 2010	
Japan	Japanese show no difference in activation of the right amygdala to averted gaze fear when viewing same-culture faces	Adams et al., 2010	
Bilateral cortical blindness, Male, Switzerland	Amygdala response to eye contact does not require an intact primary visual cortex	Burra et al., 2013	
Not applicable	Task demand or top-down modulation could play a more crucial role at gaze processing than formerly assumed in the fast-track modulator model	Burra et al., 2019	
Males, USA	The right amygdala and left amygdala/substantia innominata are sensitive to the pupil size of others, exhibiting increased activity for faces with relatively large pupils	Demos et al., 2008	
Males, Germany	Amygdala activity is specifically enhanced for fearfully widened eyes in males	Gamer & Büchel, 2009	
UK	Direct gaze leads to greater correlation between activity in the fusiform and the amygdala, a region associated with emotional responses and stimulus saliency	George et al., 2001	
Finland	Seeing direct and averted gaze activates the approach–avoidance motivational brain systems	Hietanen et al., 2008	
USA	Increases in neural processing in the amygdala facilitate the analysis of gaze cues when a person is actively monitoring for emotional gaze events	Hooker et al., 2003	
Japan	The left amygdala plays a general role in the interpretation of eye gaze direction	Kawashima et al., 1999	
Japan	The activity of the right amygdala of the person increases when another individual's gaze is directed towards him	Kawashima et al., 1999	
Germany	When anger is expressed with averted gaze, activity increases in the amygdala and the striatum in response to cultural ingroup compared to out-group (Asian)	Krämer et al., 2014	
UK	Amygdala responds to both feature-specific and configural aspects of fearful eyes	Morris et al., 2002	
Not applicable	A sub-cortical neural pathway, routed through the amygdala responds to direct eye contact, which could be a key player in the mystery of disorders of social cognition	Skuse, 2003	
Not applicable	Eye contact evokes autonomic arousal and activates the subcortical neural pathway from the retina to the superior colliculus, to the pulvinar nucleus of the thalamus and then to the amygdala	Skuse, 2006	
Australia	A large-scale neural network connected to bilateral amygdala is engaged with gaze during recognition of angry expressions	Ziaei et al., 2017	
	Gyrus responses		
Parents of children with autism, UK	Both fathers and mothers of children with autism presents more activity in left inferior frontal gyrus when viewing social emotions from the eye region	Baron-Cohen et al., 2006	
UK	Anterior superior temporal sulcus codes the direction of another's gaze direction regardless of how this information is conveyed	Carlin et al., 2011	
UK	Direct relative to averted gaze elicits stronger activation for faces in ventral occipitotemporal cortices around the fusiform gyrus, regardless of head orientation	George et al., 2001	
UK	Faces with averted gaze yields increased correlation between activity in the fusiform and the intraparietal sulcus, a region associated with shifting attention to the periphery	George et al., 2001	
USA	Perception of face identity is mediated more by regions in the inferior occipital and fusiform gyri, and perception of eye gaze is mediated more by regions in the superior temporal sulci	Hoffman & Haxby, 2000	
Females, China	Perceived gaze direction modulates neural (i.e. right superior temporal gyrus) processing of prosocial decision making	Sun et al., 2018	
Males, France	Brain regions involved in gaze processing are the occipital part of the fusiform gyrus, the right parietal lobule, the right- inferior temporal gyrus, and the middle temporal gyrus in both hemispheres	Wicker et al., 1998	
	Sulcus responses		
France	There are distinct effects of gaze direction on the involvement of the posterior and anterior regions of the superior temporal sulcus (STS)	Burra et al., 2017	
USA	Gaze perception seems to recruit the spatial cognition system in the intraparietal sulcus to encode the direction of another's gaze and to focus attention in that direction	Hoffman & Haxby., 2000	
USA	Increases in neural processing in the superior temporal sulcus support the analysis of gaze cues that provide socially meaningful spatial information	Hooker et al., 2003	
USA	Mutual gaze evokes greater activity in the superior temporal sulcus (STS) than averted gaze does, whereas fusiform gyrus (FFG) responds equivalently to mutual and averted gaze	Pelphrey et al., 2004	
USA	Anterior posterior superior temporal sulcus prefers fixated the mouth of the talker while the posterior superior temporal sulcus prefers fixations on the eye of the talker	Rennig & Beauchamp, 2018	
USA	Anterior posterior superior temporal sulcus responds more strongly to auditory and audiovisual speech than posterior posterior superior temporal sulcus eye-preferring regions	Rennig & Beauchamp, 2018	
France	Interfering with the right posterior superior sulcus neural activity transitorily disrupts the behavior of orienting toward the eyes and thus indirectly gaze perception	Saitovitch et al., 2016	
	Fronto-parietal responses		
UK	Western persons show enhanced accuracy of working memory for happy Japanese faces with averted gaze	Gregory et al., 2020	Study 1
Japan	Japanese show poorer accuracy of working memory for angry Caucasian faces with averted versus direct gaze	Gregory et al., 2020	Study 2
Japan	Japanese show no influence of working memory for happy Caucasian faces	Gregory et al., 2020	Study 2
Japan	Working memory is not modulated in Japanese when viewing Japanese faces with direct gaze	Gregory et al., 2020	Study 2
Not applicable	A core network of frontoparietal and temporal brain regions is recruited when the focus of attention is shifted in response to when it is seen that someone else shifts his or her gaze	Grosbras et al., 2005	
Australia	Recognition of angry faces is gaze-dependent, engaging the salience neural network when presented with direct gaze, but fronto-parietal areas when presented with averted gaze	Ziaei et al., 2017	
	Social brain responses		
Not applicable	The detection and perception of someone's gaze is an essential part of the development of the social brain	Babinet et al., 2022	
Females, UK	There is a considerable degree of overlap between the medial frontal areas involved in eye gaze processing and theory of mind tasks in females	Calder et al., 2002	
Not applicable	The perception of direct gaze elicits early neural processes that are related to face and eye movement encoding as well as to emotion and theory-of-mind	George & Conty, 2008	
UK	Gaze can act as an arousal cue and can modulate actions, and can activate brain regions linked to theory of mind and self-related processing	Hamilton, 2016	
Not applicable	Perceived eye contact is initially detected by a subcortical route, which then modulates the activation of the social brain as it processes the accompanying detailed sensory information	Senju & Johnson, 2009	
	EEG latencies		
USA	Neural activity earlier than 300ms may index processing of gaze change independent of social context	Carrick et al., 2007	
France	The perception of direct relative to averted gaze evokes a greater, later and longer lasting N170 ERP, suggesting that gaze contact recruit more resources than averted gaze	Conty et al., 2007	
Males, UK	Averted look yields higher EEG amplitude than direct gaze in males	Gale et al., 1972	
UK	Larger N170 ERP and N240 amplitudes in ERP response are shown for direct gaze more than for averted gaze, but only in the N240 component is this effect modulated by spatial frequency	Mares et al., 2018	
Social anxiety traits, USA	Persons with social anxiety traits show a trend for higher ERP amplitudes to averted gaze and significantly enhanced processing at late latencies	Schmitz et al., 2012	
Social anxiety traits, USA	Persons with social anxiety traits show enhanced processing of both direct and averted gaze at intermediate latencies	Schmitz et al., 2012	
Anxiety traits, Japan	In persons with social anxiety traits in Japan, negative ERP's at a right occipito-temporal site (N170) and positive ERPs at the fronto-central region (P2) are evoked by another's eye gaze	Tsuji & Shimada, 2017	

Japan	The magnetoencephalography interpeak latency 1M-2M to eyes are significantly longer than to face, which shows that it takes longer to recognize the eyes than the whole face	Watanabe et al., 1999	
	Other brain region responses		
France	Early binding of visual social signals, including gaze direction of others, engages the dorsal pathway and the premotor cortex	Conty et al., 2012	
Germany	More activation shown in dorsomedial and dorsolateral prefrontal cortices in response to cultural out-group (Asian) compared to in-group members when anger is expressed with direct gaze	Krämer et al., 2014	
Germany	Enhanced neural activation in medial and lateral prefrontal cortical areas during processing of cultural in-group compared to out-group (Asian) expressing happiness with direct gaze	Krämer et al., 2014	
Not applicable	Integrated functioning of the subcortical neural pathway in response to eye contact is modulated by one or more X-linked genes, yet to be identified	Skuse, 2006	
	Unspecified brain regions		
Japan	Greater neural responsivity to direct -versus averted- gaze fear when posed on other-culture faces in Japanese	Adams et al., 2010	
US Caucasian	Greater neural responsivity to direct -versus averted- gaze fear when posed on other-culture faces in US Caucasians	Adams et al., 2010	
Not applicable	Understanding the neural system for gaze processing requires a combination of multivariate pattern analysis approaches as well as connectivity-based methods	Carlin & Calder, 2013	
Canada	There is a neural architecture specialized for processing eyes; gaze-triggered attention is more strongly reflexive than orienting to arrows	Friesen et al., 2004	Study 1
UK	The activation of brain regions as response to gaze, is strongly modulated by the social meaning of a gaze cue and the belief that another person is directly looking	Hamilton, 2016	
Germany	Gaze direction modulates the relation between neural responses to faces and visual awareness	Madipakkam et al., 2015	
Canada	Perceived gaze direction modulates neural activity differently depending on task demands	McCrackin & Itier, 2019	
Finland	Early-stage neural processing of facial information is enhanced by another person's direct gaze when the person is faced live in contrast to when the person is faced from a picture	Pönkänen et al., 2011	
UK	There is a left visual field advantage for perception of gaze direction	Ricciardelli et al., 2002	Study 2
UK	Left visual field dominance that has been found for judgements of gaze direction is not found when persons make a judgement of pupil size	Ricciardelli et al., 2002	Study 3
France	Direct gaze or averted gaze have larger neural responses than upward gaze and eye closed	Taylor et al., 2001	
Males, France	Brain regions involved in processing of direct an averted gaze are different from those involved in face processing	Wicker et al., 1998	
	**Eye contact and space**		
	Personal space		
USA	Most persons avoid violating the personal space of a directly gazing male	Buchanan et al., 1977	Study 1
Males, USA	In contrast to females, males avoid to violate the personal space of a directly gazing female less	Buchanan et al., 1977	Study 1
Females, USA	Females avoid to violate the personal space of a directly gazing female more, and most females demonstrate a preference to violate the space of a directly gazing female	Buchanan et al., 1977	Study 1
USA	When forced to violate personal space of others in public elevators, persons choose overwhelmingly to violate persons with their back turned in contrast to direct gaze	Buchanan et al., 1977	Study 2
Males, USA	Males show little gender preference of violating the personal space of males over females.	Buchanan et al., 1977	Study 3
Females, USA	Females have a significantly preference of violating the personal space of directly gazing females over directly gazing males	Buchanan et al., 1977	Study 3
Males, USA	Males tend to use personal space and approach rate to signal dominance in response to direct gaze	Fromme & Beam, 1974	
USA	In an elevator, as eye contact of males increases, violations of personal space decreases in males and females	Hughes & Goldman, 1978	Study 1
USA	In an elevator, as eye contact of females increases, violations of personal space increases in males and females	Hughes & Goldman, 1978	Study 1
Males, USA	Males prefer to violate personal space of others in an elevator whose back is to them instead of direct gaze	Hughes & Goldman, 1978	Study 2
Females, USA	Female subjects prefer to violate personal space of others in an elevator who smile while gazing directly	Hughes & Goldman, 1978	Study 2
	Physical space		
USA	Eye contact determines physical distance to the interaction partner	Daniell & Lewis, 1972	
High-dominancy, USA	High-dominant people show increased proxemic behavior in responding to a direct gaze, while low-dominant people show a decrease	Fromme & Beam, 1974	
USA	Inconsistency between studies on the relation between gaze and distance between people, may be due to differences in research design and the nature of relationship between participants	Kleinke, 1972	
USA	Determining presence of eye contact increases with greater interaction distance	Knight et al., 1973	
Not applicable	The equilibrium theory prediction seems to only appear in distracted settings involving programmed directional gaze and not in situations involving visual interaction between naive persons	Patterson, 1975	
Males, Canada	Physical distance and eye contact are salient in impression information	Scherer, 1974	
UK	Experienced eye contact increases with increased distance between interaction partners	Stephenson & Rutter, 1970	

Abbreviations: EEG = electroencephalogram, ERP = event related potential.

Reference list

Abbott, J., Middlemiss, M., Bruce, V., Smailes, D., & Dudley, R. (2018). The effect of arousal and eye gaze direction on trust evaluations of stranger's faces: A potential pathway to paranoid thinking. *Journal of Behavior Therapy and Experimental Psychiatry*, *60*, 29–36. https://doi.org/10.1016/j.jbtep.2018.02.007

Adams, R. B., Franklin, R. G., Rule, N. O., Freeman, J. B., Kveraga, K., Hadjikhani, N., Yoshikawa, S., & Ambady, N. (2010). Culture, gaze and the neural processing of fear expressions. *Social Cognitive and Affective Neuroscience*, *5*(2–3), 340–348. https://doi.org/10.1093/scan/nsp047

Adams, R. B., & Kleck, R. E. (2003). Perceived Gaze Direction and the Processing of Facial Displays of Emotion. *Psychological Science*, *14*(6), 644–647. https://doi.org/10.1046/j.0956-7976.2003.psci_1479.x

Adams, R. B., & Kleck, R. E. (2005). Effects of Direct and Averted Gaze on the Perception of Facially Communicated Emotion. *Emotion*, *5*(1), 3–11. https://doi.org/10.1037/1528-3542.5.1.3

Adolphs, R., Baron-Cohen, S., & Tranel, D. (2002). Impaired Recognition of Social Emotions following Amygdala Damage. *Journal of Cognitive Neuroscience*, *14*(8), 1264–1274. https://doi.org/10.1162/089892902760807258

Akechi, H., Senju, A., Uibo, H., Kikuchi, Y., Hasegawa, T., & Hietanen, J. K. (2013). Attention to Eye Contact in the West and East: Autonomic Responses and Evaluative Ratings. *PLoS ONE*, *8*(3), e59312. https://doi.org/10.1371/journal.pone.0059312

Akiyama, T., Kato, M., Muramatsu, T., Saito, F., Nakachi, R., & Kashima, H. (2006a). A deficit in discriminating gaze direction in a case with right superior temporal gyrus lesion. *Neuropsychologia*, *44*(2), 161–170. https://doi.org/10.1016/j.neuropsychologia.2005.05.018

Akiyama, T., Kato, M., Muramatsu, T., Saito, F., Umeda, S., & Kashima, H. (2006b). Gaze but not arrows: A dissociative impairment after right superior temporal gyrus damage. *Neuropsychologia*, *44*(10), 1804–1810. https://doi.org/10.1016/j.neuropsychologia.2006.03.007

Akiyama, T., Kato, M., Muramatsu, T., Umeda, S., Saito, F., & Kashima, H. (2007). Unilateral Amygdala Lesions Hamper Attentional Orienting Triggered by Gaze Direction. *Cerebral Cortex*, *17*(11), 2593–2600. https://doi.org/10.1093/cercor/bhl166

Alaerts, K., Nackaerts, E., Meyns, P., Swinnen, S. P., & Wenderoth, N. (2011). Action and Emotion Recognition from Point Light Displays: An Investigation of Gender Differences. *PLoS ONE*, *6*(6), e20989. https://doi.org/10.1371/journal.pone.0020989

Amalfitano, J. G., & Kalt, N. C. (1977). Effects of Eye Contact on the Evaluation of Job Applicants. *Journal of Employment Counseling*, *14*(1), 46–48. https://doi.org/10.1002/j.2161-1920.1977.tb00637.x

Argyle, M., Lefebvre, L., & Cook, M. (1974). The meaning of five patterns of gaze. *European Journal of Social Psychology*, *4*(2), 125–136. https://doi.org/10.1002/ejsp.2420040202

Babinet, M.-N., Cublier, M., Demily, C., & Michael, G. A. (2022). Eye Direction Detection and Perception as Premises of a Social Brain: A Narrative Review of Behavioral and Neural Data. *Cognitive, Affective, & Behavioral Neuroscience*, *22*(1), 1–20. https://doi.org/10.3758/s13415-021-00959-w

Balter, L. J. T., Hulsken, S., Aldred, S., Drayson, M. T., Higgs, S., Veldhuijzen Van Zanten, J. J. C. S., Raymond, J. E., & Bosch, J. A. (2018). Low-grade inflammation decreases emotion recognition – Evidence from the vaccination model of inflammation. *Brain, Behavior, and Immunity*, *73*, 216–221. https://doi.org/10.1016/j.bbi.2018.05.006

Baron-Cohen, S., Ring, H., Chitnis, X., Wheelwright, S., Gregory, L., Williams, S., Brammer, M., & Bullmore, E. (2006). fMRI of parents of children with Asperger Syndrome: A pilot study. *Brain and Cognition*, *61*(1), 122–130. https://doi.org/10.1016/j.bandc.2005.12.011

Baron-Cohen, S., Wheelwright, S., & Jolliffe, A. T. (1997). Is There a "Language of the Eyes"? Evidence from Normal Adults, and Adults with Autism or Asperger Syndrome. *Visual Cognition*, *4*(3), 311–331. https://doi.org/10.1080/713756761

Beebe, S. A. (1974). Eye contact: A nonverbal determinant of speaker credibility. *The Speech Teacher*, *23*(1), 21–25. https://doi.org/10.1080/03634527409378052

Bindemann, M., Mike Burton, A., & Langton, S. R. H. (2008). How do eye gaze and facial expression interact? *Visual Cognition*, *16*(6), 708–733. https://doi.org/10.1080/13506280701269318

Blais, C., Roy, C., Fiset, D., Arguin, M., & Gosselin, F. (2012). The eyes are not the window to basic emotions. *Neuropsychologia*, *50*(12), 2830–2838. https://doi.org/10.1016/j.neuropsychologia.2012.08.010

Body Language in Different Cultures. (2014). *US-China Foreign Language*, *12*(12). https://doi.org/10.17265/1539-8080/2014.12.008

Bond, M., & Goodman, G. (1980). Gaze patterns and interaction contexts: Effects on personality impressions and attributions. *Psychologia: An International Journal of Psychology in the Orient*, *23*(2), 70–77.

Buchanan, D. R., Goldman, M., & Juhnke, R. (1977). Eye Contact, Sex, and the Violation of Personal Space. *The Journal of Social Psychology*, *103*(1), 19–25. https://doi.org/10.1080/00224545.1977.9713291

Bull, R., & Gibson-Robinson, E. (1981). The Influences of Eye-Gaze, Style of Dress, and Locality on the Amounts of Money Donated to a Charity. *Human Relations*, *34*(10), 895–905. https://doi.org/10.1177/001872678103401005

Burgoon, J. K., Manusov, V., Mineo, P., & Hale, J. L. (1985). Effects of gaze on hiring, credibility, attraction and relational message interpretation. *Journal of Nonverbal Behavior*, *9*(3), 133–146. https://doi.org/10.1007/BF01000735

Burra, N., Baker, S., & George, N. (2017). Processing of gaze direction within the N170/M170 time window: A combined EEG/MEG study. *Neuropsychologia*, *100*, 207–219. https://doi.org/10.1016/j.neuropsychologia.2017.04.028

Burra, N., Hervais-Adelman, A., Kerzel, D., Tamietto, M., De Gelder, B., & Pegna, A. J. (2013). Amygdala Activation for Eye Contact Despite Complete Cortical Blindness. *Journal of Neuroscience*, *33*(25), 10483–10489. https://doi.org/10.1523/JNEUROSCI.3994-12.2013

Burra, N., & Kerzel, D. (2021). Meeting another's gaze shortens subjective time by capturing attention. *Cognition*, *212*, 104734. https://doi.org/10.1016/j.cognition.2021.104734

Burra, N., Mares, I., & Senju, A. (2019). The influence of top-down modulation on the processing of direct gaze. *WIREs Cognitive Science*, *10*(5), e1500. https://doi.org/10.1002/wcs.1500

Caldara, R., Schyns, P., Mayer, E., Smith, M. L., Gosselin, F., & Rossion, B. (2005). Does Prosopagnosia Take the Eyes Out of Face Representations? Evidence for a Defect in Representing Diagnostic Facial Information following Brain Damage. *Journal of Cognitive Neuroscience*, *17*(10), 1652–1666. https://doi.org/10.1162/089892905774597254

Calder, A. J., Lawrence, A. D., Keane, J., Scott, S. K., Owen, A. M., Christoffels, I., & Young, A. W. (2002). Reading the mind from eye gaze. *Neuropsychologia*, *40*(8), 1129–1138. https://doi.org/10.1016/S0028-3932(02)00008-8

Campbell, D. E., & Lancioni, G. E. (1979). The Effects of Staring and Pew Invasion in Church Settings. *The Journal of Social Psychology*, *108*(1), 19–24. https://doi.org/10.1080/00224545.1979.9711956

Cañadas, E., & Lupiáñez, J. (2012). Spatial interference between gaze direction and gaze location: A study on the eye contact effect. *Quarterly Journal of Experimental Psychology*, *65*(8), 1586–1598. https://doi.org/10.1080/17470218.2012.659190

Caproni, V., Levine, D., O'neal, E., McDonald, P., & Garwood, G. (1977). Seating Position, Instructor's Eye Contact Availability, and Student Participation in a Small Seminar. *The Journal of Social Psychology*, *103*(2), 315–316. https://doi.org/10.1080/00224545.1977.9713335

Carlin, J. D., & Calder, A. J. (2013). The neural basis of eye gaze processing. *Current Opinion in Neurobiology*, *23*(3), 450–455. https://doi.org/10.1016/j.conb.2012.11.014

Carlin, J. D., Calder, A. J., Kriegeskorte, N., Nili, H., & Rowe, J. B. (2011). A Head View-Invariant Representation of Gaze Direction in Anterior Superior Temporal Sulcus. *Current Biology*, *21*(21), 1817–1821. https://doi.org/10.1016/j.cub.2011.09.025

Carrick, O. K., Thompson, J. C., Epling, J. A., & Puce, A. (2007). It's all in the eyes: Neural responses to socially significant gaze shifts. *NeuroReport*, *18*(8), 763–766. https://doi.org/10.1097/WNR.0b013e3280ebb44b

Chen, T., Helminen, T. M., & Hietanen, J. K. (2017). Affect in the eyes: Explicit and implicit evaluations. *Cognition and Emotion*, *31*(6), 1070–1082. https://doi.org/10.1080/02699931.2016.1188059

Collova, J. R., Kloth, N., Crookes, K., Burton, N., Chan, C. Y. H., Hsiao, J. H., & Rhodes, G. (2017). A new other-race effect for gaze perception. *Journal of Experimental Psychology: Human Perception and Performance*, *43*(11), 1857–1863. https://doi.org/10.1037/xhp0000460

Conty, L., Dezecache, G., Hugueville, L., & Grèzes, J. (2012). Early Binding of Gaze, Gesture, and Emotion: Neural Time Course and Correlates. *The Journal of Neuroscience*, *32*(13), 4531–4539. https://doi.org/10.1523/JNEUROSCI.5636-11.2012

Conty, L., Gimmig, D., Belletier, C., George, N., & Huguet, P. (2010). The cost of being watched: Stroop interference increases under concomitant eye contact. *Cognition*, *115*(1), 133–139. https://doi.org/10.1016/j.cognition.2009.12.005

Conty, L., N'Diaye, K., Tijus, C., & George, N. (2007). When eye creates the contact! ERP evidence for early dissociation between direct and averted gaze motion processing. *Neuropsychologia*, *45*(13), 3024–3037. https://doi.org/10.1016/j.neuropsychologia.2007.05.017

Dai, Y. (2021). Foreign Language Teachers' Emotion Recognition in College Oral English Classroom Teaching. *Frontiers in Psychology*, *12*, 782379. https://doi.org/10.3389/fpsyg.2021.782379

Daniell, R. J., & Lewis, P. (1972). Stability of eye contact and physical distance across a series of structured interviews. *Journal of Consulting and Clinical Psychology*, *39*(1), 172–172. https://doi.org/10.1037/h0033160

Demos, K. E., Kelley, W. M., Ryan, S. L., Davis, F. C., & Whalen, P. J. (2008). Human Amygdala Sensitivity to the Pupil Size of Others. *Cerebral Cortex*, *18*(12), 2729–2734. https://doi.org/10.1093/cercor/bhn034

Doi, H., & Ueda, K. (2007). Searching for a Perceived Stare in the Crowd. *Perception*, *36*(5), 773–780. https://doi.org/10.1068/p5614

Dovidio, J. F., & Ellyson, S. L. (1982). Decoding Visual Dominance: Attributions of Power Based on Relative Percentages of Looking While Speaking and Looking While Listening. *Social Psychology Quarterly*, *45*(2), 106. https://doi.org/10.2307/3033933

Eddy, C. M., & Hansen, P. C. (2020). Predictors of performance on the Reading the Mind in the Eyes Test. *PLOS ONE*, *15*(7), e0235529. https://doi.org/10.1371/journal.pone.0235529

Elgar, K., Campbell, R., & Skuse, D. (2002). Are you looking at me? Accuracy in processing line–of–sight in Turner syndrome. *Proceedings of the Royal Society of London. Series B: Biological Sciences*, *269*(1508), 2415–2422. https://doi.org/10.1098/rspb.2002.2173

Ellsworth, P. C., Carlsmith, J. M., & Henson, A. (1972). The stare as a stimulus to flight in human subjects: A series of field experiments. *Journal of Personality and Social Psychology*, *21*(3), 302–311. https://doi.org/10.1037/h0032323

Ellsworth, P., & Carlsmith, J. M. (1973). Eye contact and gaze aversion in an aggressive encounter. *Journal of Personality and Social Psychology*, *28*(2), 280–292. https://doi.org/10.1037/h0035779

Ellsworth, P., & Ross, L. (1975). Intimacy in response to direct gaze. *Journal of Experimental Social Psychology*, *11*(6), 592–613.

Elman, D., Schulte, D. C., & Bukoff, A. (1977). Effects of facial expression and stare duration on walking speed: Two field experiments. *Environmental Psychology and Nonverbal Behavior*, *2*(2), 93–99. https://doi.org/10.1007/BF01145825

Ewing, L., Rhodes, G., & Pellicano, E. (2010). Have you got the look? Gaze direction affects judgements of facial attractiveness. *Visual Cognition*, *18*(3), 321–330. https://doi.org/10.1080/13506280902965599

Fathi, M., Bateson, M., & Nettle, D. (2014). Effects of Watching Eyes and Norm Cues on Charitable Giving in a Surreptitious Behavioral Experiment. *Evolutionary Psychology*, *12*(5), 878–887. https://doi.org/10.1177/147470491401200502

Fox, E., Mathews, A., Calder, A. J., & Yiend, J. (2007). Anxiety and sensitivity to gaze direction in emotionally expressive faces. *Emotion*, *7*(3), 478–486. https://doi.org/10.1037/1528-3542.7.3.478

Framorando, D., George, N., Kerzel, D., & Burra, N. (2016). Straight gaze facilitates face processing but does not cause involuntary attentional capture. *Visual Cognition*, *24*(7–8), 381–391. https://doi.org/10.1080/13506285.2017.1285840

Friesen, C. K., & Kingstone, A. (1998). The eyes have it! Reflexive orienting is triggered by nonpredictive gaze. *Psychonomic Bulletin & Review*, *5*(3), 490–495. https://doi.org/10.3758/BF03208827

Friesen, C. K., Moore, C., & Kingstone, A. (2005). Does gaze direction really trigger a reflexive shift of spatial attention? *Brain and Cognition*, *57*(1), 66–69. https://doi.org/10.1016/j.bandc.2004.08.025

Friesen, C. K., Ristic, J., & Kingstone, A. (2004). Attentional Effects of Counterpredictive Gaze and Arrow Cues. *Journal of Experimental Psychology: Human Perception and Performance*, *30*(2), 319–329. https://doi.org/10.1037/0096-1523.30.2.319

Friesen, J. P., Kawakami, K., Vingilis-Jaremko, L., Caprara, R., Sidhu, D. M., Williams, A., Hugenberg, K., Rodríguez-Bailón, R., Cañadas, E., & Niedenthal, P. (2019). Perceiving happiness in an intergroup context: The role of race and attention to the eyes in differentiating between true and false smiles. *Journal of Personality and Social Psychology*, *116*(3), 375–395. https://doi.org/10.1037/pspa0000139

Frischen, A., Bayliss, A. P., & Tipper, S. P. (2007). Gaze cueing of attention: Visual attention, social cognition, and individual differences. *Psychological Bulletin*, *133*(4), 694–724. https://doi.org/10.1037/0033-2909.133.4.694

Fromme, D. K., & Beam, D. C. (1974). Dominance and sex differences in nonverbal responses to differential eye contact. *Journal of Research in Personality*, *8*(1), 76–87. https://doi.org/10.1016/0092-6566(74)90047-6

Fujiwara, E. (2018). Looking at the eyes interferes with facial emotion recognition in alexithymia. *Journal of Abnormal Psychology*, *127*(6), 571–577. https://doi.org/10.1037/abn0000361

Gale, A., Lucas, B., Nissim, R., & Harpham, B. (1972). Some EEG Correlates of Face-to-Face Contact. *British Journal of Social and Clinical Psychology*, *11*(4), 326–332. https://doi.org/10.1111/j.2044-8260.1972.tb00822.x

Gamer, M., & Buchel, C. (2009). Amygdala Activation Predicts Gaze toward Fearful Eyes. *Journal of Neuroscience*, *29*(28), 9123–9126. https://doi.org/10.1523/JNEUROSCI.1883-09.2009

Gamer, M., & Hecht, H. (2007). Are you looking at me? Measuring the cone of gaze. *Journal of Experimental Psychology: Human Perception and Performance*, *33*(3), 705–715. https://doi.org/10.1037/0096-1523.33.3.705

Gendron, M. (2017). Revisiting diversity: Cultural variation reveals the constructed nature of emotion perception. *Current Opinion in Psychology*, *17*, 145–150. https://doi.org/10.1016/j.copsyc.2017.07.014

George, N., & Conty, L. (2008). Facing the gaze of others. *Neurophysiologie Clinique/Clinical Neurophysiology*, *38*(3), 197–207. https://doi.org/10.1016/j.neucli.2008.03.001

George, N., Driver, J., & Dolan, R. J. (2001). Seen Gaze-Direction Modulates Fusiform Activity and Its Coupling with Other Brain Areas during Face Processing. *NeuroImage*, *13*(6), 1102–1112. https://doi.org/10.1006/nimg.2001.0769

Gregory, S. E. A., Langton, S. R. H., Yoshikawa, S., & Jackson, M. C. (2020). A cross-cultural investigation into the influence of eye gaze on working memory for happy and angry faces. *Cognition and Emotion*, *34*(8), 1561–1572. https://doi.org/10.1080/02699931.2020.1782353

Grosbras, M., Laird, A. R., & Paus, T. (2005). Cortical regions involved in eye movements, shifts of attention, and gaze perception. *Human Brain Mapping*, *25*(1), 140–154. https://doi.org/10.1002/hbm.20145

Guariglia, P., Piccardi, L., Giaimo, F., Alaimo, S., MiccichÃ¨, G., & Antonucci, G. (2015). The eyes test is influenced more by artistic inclination and less by sex. *Frontiers in Human Neuroscience*, *9*. https://doi.org/10.3389/fnhum.2015.00292

Hadders-Algra, M. (2022). Human face and gaze perception is highly context specific and involves bottom-up and top-down neural processing. *Neuroscience & Biobehavioral Reviews*, *132*, 304–323. https://doi.org/10.1016/j.neubiorev.2021.11.042

Hamilton, A. F. D. C. (2016). Gazing at me: The importance of social meaning in understanding direct-gaze cues. *Philosophical Transactions of the Royal Society B: Biological Sciences*, *371*(1686), 20150080. https://doi.org/10.1098/rstb.2015.0080

Hietanen, J. K., Leppänen, J. M., Peltola, M. J., Linna-aho, K., & Ruuhiala, H. J. (2008). Seeing direct and averted gaze activates the approach–avoidance motivational brain systems. *Neuropsychologia*, *46*(9), 2423–2430. https://doi.org/10.1016/j.neuropsychologia.2008.02.029

Hietanen, J. O., Syrjämäki, A. H., Zilliacus, P. K., & Hietanen, J. K. (2018). Eye contact reduces lying. *Consciousness and Cognition*, *66*, 65–73. https://doi.org/10.1016/j.concog.2018.10.006

Hoffman, E. A., & Haxby, J. V. (2000). Distinct representations of eye gaze and identity in the distributed human neural system for face perception. *Nature Neuroscience*, *3*(1), 80–84. https://doi.org/10.1038/71152

Honma, M. (2013). Hyper-volume of eye-contact perception and social anxiety traits. *Consciousness and Cognition*, *22*(1), 167–173. https://doi.org/10.1016/j.concog.2012.12.002

Hooker, C. I., Paller, K. A., Gitelman, D. R., Parrish, T. B., Mesulam, M.-M., & Reber, P. J. (2003). Brain networks for analyzing eye gaze. *Cognitive Brain Research*, *17*(2), 406–418. https://doi.org/10.1016/S0926-6410(03)00143-5

Hu, Z., Gendron, M., Liu, Q., Zhao, G., & Li, H. (2017). Trait Anxiety Impacts the Perceived Gaze Direction of Fearful But Not Angry Faces. *Frontiers in Psychology*, *8*, 1186. https://doi.org/10.3389/fpsyg.2017.01186

Hughes, J., & Goldman, M. (1978). Eye Contact, Facial Expression, Sex, and the Violation of Personal Space. *Perceptual and Motor Skills*, *46*(2), 579–584. https://doi.org/10.2466/pms.1978.46.2.579

Isernia, S., Sokolov, A. N., Fallgatter, A. J., & Pavlova, M. A. (2020). Untangling the Ties Between Social Cognition and Body Motion: Gender Impact. *Frontiers in Psychology*, *11*, 128. https://doi.org/10.3389/fpsyg.2020.00128

Itier, R. J., Villate, C., & Ryan, J. D. (2007). Eyes always attract attention but gaze orienting is task-dependent: Evidence from eye movement monitoring. *Neuropsychologia*, *45*(5), 1019–1028. https://doi.org/10.1016/j.neuropsychologia.2006.09.004

Jack, R. E., Caldara, R., & Schyns, P. G. (2012). Internal representations reveal cultural diversity in expectations of facial expressions of emotion. *Journal of Experimental Psychology: General*, *141*(1), 19–25. https://doi.org/10.1037/a0023463

Kampe, K. K. W., Frith, C. D., Dolan, R. J., & Frith, U. (2001). Reward value of attractiveness and gaze. *Nature*, *413*(6856), 589–589. https://doi.org/10.1038/35098149

Karandashev, V., Zarubko, E., Artemeva, V., Neto, F., Surmanidze, L., & Feybesse, C. (2016). Sensory Values in Romantic Attraction in Four Europeans Countries: Gender and Cross-Cultural Comparison. *Cross-Cultural Research*, *50*(5), 478–504. https://doi.org/10.1177/1069397116674446

Kawashima, R., Sugiura, M., Kato, T., Nakamura, A., Hatano, K., Ito, K., Fukuda, H., Kojima, S., & Nakamura, K. (1999). The human amygdala plays an important role in gaze monitoring. *Brain*, *122*(4), 779–783. https://doi.org/10.1093/brain/122.4.779

Kellerman, J., Lewis, J., & Laird, J. D. (1989). Looking and loving: The effects of mutual gaze on feelings of romantic love. *Journal of Research in Personality*, *23*(2), 145–161. https://doi.org/10.1016/0092-6566(89)90020-2

Kelly, E. W., & True, J. H. (1980). Eye Contact and Communication of Facilitative Conditions. *Perceptual and Motor Skills*, *51*(3), 815–820. https://doi.org/10.2466/pms.1980.51.3.815

Kendon, A., & Cook, M. (1969). The consistency of gaze patterns in social interaction. *British Journal of Psychology*, *60*(4), 481–494. https://doi.org/10.1111/j.2044-8295.1969.tb01222.x

Kim, G., Seong, S. H., Hong, S.-S., & Choi, E. (2022). Impact of face masks and sunglasses on emotion recognition in South Koreans. *PLOS ONE*, *17*(2), e0263466. https://doi.org/10.1371/journal.pone.0263466

Kirkland, R., Peterson, E., Baker, C., Miller, S., & Pulos, S. (2013). Meta-analysis Reveals Adult Female Superiority in “Reading the Mind in the Eyes Test.” *North American Journal of Psychology*, *15*(1), 121–146.

Kleinke, C. (1972). Interpersonal attraction as it relates to gaze and distance between people. *Representative Research in Social Psychology*, *3*, 105–120.

Kleinke, C. L. (1977). Compliance to requests made by gazing and touching experimenters in field settings. *Journal of Experimental Social Psychology*, *13*(3), 218–223. https://doi.org/10.1016/0022-1031(77)90044-0

Kleinke, C. L. (1986). Gaze and eye contact: A research review. *Psychological Bulletin*, *100*(1), 78–100. https://doi.org/10.1037/0033-2909.100.1.78

Kleinke, C. L., Bustos, A. A., Meeker, F. B., & Staneski, R. A. (1973). Effects of self-attributed and other-attributed gaze on interpersonal evaluations between males and females. *Journal of Experimental Social Psychology*, *9*(2), 154–163. https://doi.org/10.1016/0022-1031(73)90007-3

Knight, D. J., Langmeyer, D., & Lundgren, D. C. (1973a). Eye-Contact, Distance, and Affiliation: The Role of Observer Bias. *Sociometry*, *36*(3), 390. https://doi.org/10.2307/2786340

Knight, D. J., Langmeyer, D., & Lundgren, D. C. (1973b). Eye-Contact, Distance, and Affiliation: The Role of Observer Bias. *Sociometry*, *36*(3), 390. https://doi.org/10.2307/2786340

Krämer, K., Bente, G., Kuzmanovic, B., Barisic, I., Pfeiffer, U. J., Georgescu, A. L., & Vogeley, K. (2014). Neural correlates of emotion perception depending on culture and gaze direction. *Culture and Brain*, *2*(1), 27–51. https://doi.org/10.1007/s40167-014-0013-9

Krämer, K., Bente, G., Luo, S., Pfeiffer, U. J., Han, S., & Vogeley, K. (2013). Influence of Ethnic Group-Membership and Gaze Direction on the Perception of Emotions. A Cross-Cultural Study between Germany and China. *PLoS ONE*, *8*(6), e66335. https://doi.org/10.1371/journal.pone.0066335

Kret, M. E., & De Gelder, B. (2012). Islamic Headdress Influences How Emotion is Recognized from the Eyes. *Frontiers in Psychology*, *3*. https://doi.org/10.3389/fpsyg.2012.00110

Kreysa, H., Kessler, L., & Schweinberger, S. R. (2016). Direct Speaker Gaze Promotes Trust in Truth-Ambiguous Statements. *PLOS ONE*, *11*(9), e0162291. https://doi.org/10.1371/journal.pone.0162291

Langton, S. R. H. (2000). The Mutual Influence of Gaze and Head Orientation in the Analysis of Social Attention Direction. *The Quarterly Journal of Experimental Psychology Section A*, *53*(3), 825–845. https://doi.org/10.1080/713755908

Langton, S. R. H., Watt, R. J., & Bruce, V. (2000). Do the eyes have it? Cues to the direction of social attention. *Trends in Cognitive Sciences*, *4*(2), 50–59. https://doi.org/10.1016/S1364-6613(99)01436-9

Lawrence, K., Campbell, R., Swettenham, J., Terstegge, J., Akers, R., Coleman, M., & Skuse, D. (2003). Interpreting gaze in Turner syndrome: Impaired sensitivity to intention and emotion, but preservation of social cueing. *Neuropsychologia*, *41*(8), 894–905. https://doi.org/10.1016/S0028-3932(03)00002-2

Lawson, R. (2015). I just love the attention: Implicit preference for direct eye contact. *Visual Cognition*, *23*(4), 450–488. https://doi.org/10.1080/13506285.2015.1039101

Liang, J., Zou, Y.-Q., Liang, S.-Y., Wu, Y.-W., & Yan, W.-J. (2021). Emotional Gaze: The Effects of Gaze Direction on the Perception of Facial Emotions. *Frontiers in Psychology*, *12*, 684357. https://doi.org/10.3389/fpsyg.2021.684357

Ma, X., Fu, M., Zhang, X., Song, X., Becker, B., Wu, R., Xu, X., Gao, Z., Kendrick, K., & Zhao, W. (2022). Own Race Eye-Gaze Bias for All Emotional Faces but Accuracy Bias Only for Sad Expressions. *Frontiers in Neuroscience*, *16*, 852484. https://doi.org/10.3389/fnins.2022.852484

MacDonald, K. (2009). Patient-Clinician Eye Contact: Social Neuroscience and Art of Clinical Engagement. *Postgraduate Medicine*, *121*(4), 136–144. https://doi.org/10.3810/pgm.2009.07.2039

Macrae, C. N., Hood, B. M., Milne, A. B., Rowe, A. C., & Mason, M. F. (2002). Are You Looking at Me? Eye Gaze and Person Perception. *Psychological Science*, *13*(5), 460–464. https://doi.org/10.1111/1467-9280.00481

Madipakkam, A. R., Rothkirch, M., Guggenmos, M., Heinz, A., & Sterzer, P. (2015). Gaze Direction Modulates the Relation between Neural Responses to Faces and Visual Awareness. *The Journal of Neuroscience*, *35*(39), 13287–13299. https://doi.org/10.1523/JNEUROSCI.0815-15.2015

Mai, X., Ge, Y., Tao, L., Tang, H., Liu, C., & Luo, Y.-J. (2011). Eyes Are Windows to the Chinese Soul: Evidence from the Detection of Real and Fake Smiles. *PLoS ONE*, *6*(5), e19903. https://doi.org/10.1371/journal.pone.0019903

Mares, I., Smith, M. L., Johnson, M. H., & Senju, A. (2018). Revealing the neural time-course of direct gaze processing via spatial frequency manipulation of faces. *Biological Psychology*, *135*, 76–83. https://doi.org/10.1016/j.biopsycho.2018.03.001

Mareschal, I., Calder, A. J., & Clifford, C. W. G. (2013). Humans Have an Expectation That Gaze Is Directed Toward Them. *Current Biology*, *23*(8), 717–721. https://doi.org/10.1016/j.cub.2013.03.030

Martin, W. W., & Gardner, S. N. (1979). The Relative Effects of Eye-Gaze and Smiling on Arousal in Asocial Situations. *The Journal of Psychology*, *102*(2), 253–259. https://doi.org/10.1080/00223980.1979.9923495

Mason, M., Hood, B., & Macrae, C. N. (2004). Look into my eyes: Gaze direction and person memory. *Memory*, *12*(5), 637–643. https://doi.org/10.1080/09658210344000152

Mathews, A., Fox, E., Yiend, J., & Calder, A. (2003). The face of fear: Effects of eye gaze and emotion on visual attention. *Visual Cognition*, *10*(7), 823–835. https://doi.org/10.1080/13506280344000095

McCrackin, S. D., & Itier, R. J. (2019). Perceived Gaze Direction Differentially Affects Discrimination of Facial Emotion, Attention, and Gender – An ERP Study. *Frontiers in Neuroscience*, *13*, 517. https://doi.org/10.3389/fnins.2019.00517

Megías-Robles, A., Gutiérrez-Cobo, M. J., Cabello, R., Gómez-Leal, R., Baron-Cohen, S., & Fernández-Berrocal, P. (2020). The 'Reading the mind in the Eyes' test and emotional intelligence. *Royal Society Open Science*, *7*(9), 201305. https://doi.org/10.1098/rsos.201305

Milders, M., Hietanen, J. K., Leppänen, J. M., & Braun, M. (2011). Detection of emotional faces is modulated by the direction of eye gaze. *Emotion*, *11*(6), 1456–1461. https://doi.org/10.1037/a0022901

Moore, H. T., & Gilliland, A. R. (1921). The Measurement of Aggressiveness. *Journal of Applied Psychology*, *5*(2), 97–118. https://doi.org/10.1037/h0073691

Morris, J. S., deBonis, M., & Dolan, R. J. (2002). Human Amygdala Responses to Fearful Eyes. *NeuroImage*, *17*(1), 214–222. https://doi.org/10.1006/nimg.2002.1220

Naiman, T., & Breed, G. (1974). Gaze duration as a cue for judging conversational tone. *Representative Research in Social Psychology*, *5*, 115–122.

Napieralski, L. P., Brooks, C. I., & Droney, J. M. (1995). The Effect of Duration of Eye Contact on American College Students' Attributions of State, Trait, and Test Anxiety. *The Journal of Social Psychology*, *135*(3), 273–280. https://doi.org/10.1080/00224545.1995.9713957

Nichols, K. A., & Champness, B. G. (1971). Eye gaze and the GSR. *Journal of Experimental Social Psychology*, *7*(6), 623–626. https://doi.org/10.1016/0022-1031(71)90024-2

Patterson, M. L. (1975). Eye Contact and Distance: A Re-Examination of Measurement Problems. *Personality and Social Psychology Bulletin*, *1*(4), 600–603. https://doi.org/10.1177/014616727500100411

Pavlova, M. A., Romagnano, V., Kubon, J., Isernia, S., Fallgatter, A. J., & Sokolov, A. N. (2022). Ties between reading faces, bodies, eyes, and autistic traits. *Frontiers in Neuroscience*, *16*, 997263. https://doi.org/10.3389/fnins.2022.997263

Pelphrey, K. A., Viola, R. J., & McCarthy, G. (2004). When Strangers Pass: Processing of Mutual and Averted Social Gaze in the Superior Temporal Sulcus. *Psychological Science*, *15*(9), 598–603. https://doi.org/10.1111/j.0956-7976.2004.00726.x

Pönkänen, L. M., Alhoniemi, A., Leppänen, J. M., & Hietanen, J. K. (2011). Does it make a difference if I have an eye contact with you or with your picture? An ERP study. *Social Cognitive and Affective Neuroscience*, *6*(4), 486–494. https://doi.org/10.1093/scan/nsq068

Rennig, J., & Beauchamp, M. S. (2018). Free viewing of talking faces reveals mouth and eye preferring regions of the human superior temporal sulcus. *NeuroImage*, *183*, 25–36. https://doi.org/10.1016/j.neuroimage.2018.08.008

Ricciardelli, P. (2002). A left visual field advantage in perception of gaze direction. *Neuropsychologia*, *40*(7), 769–777. https://doi.org/10.1016/S0028-3932(01)00190-7

Ricciardelli, P., Baylis, G., & Driver, J. (2000). The positive and negative of human expertise in gaze perception. *Cognition*, *77*(1), B1–B14. https://doi.org/10.1016/S0010-0277(00)00092-5

Ricciardelli, P., & Driver, J. (2008). Effects of Head Orientation on Gaze Perception: How Positive Congruency Effects Can be Reversed. *Quarterly Journal of Experimental Psychology*, *61*(3), 491–504. https://doi.org/10.1080/17470210701255457

Rimmele, U., & Lobmaier, J. S. (2012). Stress increases the feeling of being looked at. *Psychoneuroendocrinology*, *37*(2), 292–298. https://doi.org/10.1016/j.psyneuen.2011.06.013

Rutter, D. R., & Stephenson, G. M. (1979). The functions of Looking: Effects of friendship on gaze. *British Journal of Social and Clinical Psychology*, *18*(2), 203–205. https://doi.org/10.1111/j.2044-8260.1979.tb00326.x

Saitovitch, A., Popa, T., Lemaitre, H., Rechtman, E., Lamy, J.-C., Grévent, D., Calmon, R., Meunier, S., Brunelle, F., Samson, Y., Boddaert, N., & Zilbovicius, M. (2016). Tuning Eye-Gaze Perception by Transitory STS Inhibition. *Cerebral Cortex*, *26*(6), 2823–2831. https://doi.org/10.1093/cercor/bhw045

Scherer, S. E. (1974). Influence of Proximity and Eye Contact on Impression Formation. *Perceptual and Motor Skills*, *38*(2), 538–538. https://doi.org/10.2466/pms.1974.38.2.538

Scherwitz, L., & Helmreich, R. (1973). Interactive effects of eye contact and verbal content on interpersonal attraction in dyads. *Journal of Personality and Social Psychology*, *25*(1), 6–14. https://doi.org/10.1037/h0034270

Schmidtmann, G., Logan, A. J., Carbon, C.-C., Loong, J. T., & Gold, I. (2020). In the Blink of an Eye: Reading Mental States From Briefly Presented Eye Regions. *I-Perception*, *11*(5), 204166952096111. https://doi.org/10.1177/2041669520961116

Schmitz, J., Scheel, C. N., Rigon, A., Gross, J. J., & Blechert, J. (2012). You don't like me, do you? Enhanced ERP responses to averted eye gaze in social anxiety. *Biological Psychology*, *91*(2), 263–269. https://doi.org/10.1016/j.biopsycho.2012.07.004

Schulze, L., Lobmaier, J. S., Arnold, M., & Renneberg, B. (2013). All eyes on me?! Social anxiety and self-directed perception of eye gaze. *Cognition & Emotion*, *27*(7), 1305–1313. https://doi.org/10.1080/02699931.2013.773881

Senju, A., & Hasegawa, T. (2005). Direct gaze captures visuospatial attention. *Visual Cognition*, *12*(1), 127–144. https://doi.org/10.1080/13506280444000157

Senju, A., & Johnson, M. H. (2009). The eye contact effect: Mechanisms and development. *Trends in Cognitive Sciences*, *13*(3), 127–134. https://doi.org/10.1016/j.tics.2008.11.009

Skuse, D. (2003). Fear Recognition and the Neural Basis of Social Cognition. *Child and Adolescent Mental Health*, *8*(2), 50–60. https://doi.org/10.1111/1475-3588.00047

Skuse, D. (2006). Genetic influences on the neural basis of social cognition. *Philosophical Transactions of the Royal Society B: Biological Sciences*, *361*(1476), 2129–2141. https://doi.org/10.1098/rstb.2006.1935

Slessor, G., Phillips, L. H., & Bull, R. (2008). Age-related declines in basic social perception: Evidence from tasks assessing eye-gaze processing. *Psychology and Aging*, *23*(4), 812–822. https://doi.org/10.1037/a0014348

Söderberg, A., Sundbaum, J. K., & Engström, Å. (2017). Nursing Students' Reflections After Meetings With Patients and Their Relatives Enacted by Professional Actors: Being Touched and Feeling Empathy. *Issues in Mental Health Nursing*, *38*(2), 139–144. https://doi.org/10.1080/01612840.2016.1251517

Song, J., Wei, Y., & Ke, H. (2019). The effect of emotional information from eyes on empathy for pain: A subliminal ERP study. *PLOS ONE*, *14*(12), e0226211. https://doi.org/10.1371/journal.pone.0226211

Stanley, J. T., Zhang, X., Fung, H. H., & Isaacowitz, D. M. (2013). Cultural differences in gaze and emotion recognition: Americans contrast more than Chinese. *Emotion*, *13*(1), 36–46. https://doi.org/10.1037/a0029209

Strom, J., & Buck, R. (1979). Staring and participants' sex: Physiological and subjective reactive responses. *Personality and Social Psychology Bulletin*, *5*, 114–117.

Sun, D., Shao, R., Wang, Z., & Lee, T. M. C. (2018). Perceived Gaze Direction Modulates Neural Processing of Prosocial Decision Making. *Frontiers in Human Neuroscience*, *12*, 52. https://doi.org/10.3389/fnhum.2018.00052

Taylor, A., Bleiker, J., & Hodgson, D. (2021). Compassionate communication: Keeping patients at the heart of practice in an advancing radiographic workforce. *Radiography*, *27*, S43–S49. https://doi.org/10.1016/j.radi.2021.07.014

Taylor, M. J., George, N., & Ducorps, A. (2001). Magnetoencephalographic evidence of early processing of direction of gaze in humans. *Neuroscience Letters*, *316*(3), 173–177. https://doi.org/10.1016/S0304-3940(01)02378-3

Teske, J. A. (1988). Seeing Her Looking at You: Acquaintance and Variation in the Judgment of Gaze Depth. *The American Journal of Psychology*, *101*(2), 239. https://doi.org/10.2307/1422837

Tessler, R., & Sushelsky, L. (1978). Effects of eye contact and social status on the perception of a job applicant in an employment interviewing situation. *Journal of Vocational Behavior*, *13*, 338–347.

Thayer, S. (1969). The Effect of Interpersonal Looking Duration on Dominance Judgments. *The Journal of Social Psychology*, *79*(2), 285–286. https://doi.org/10.1080/00224545.1969.9922427

Thayer, S., & Schiff, W. (1974). Observer judgment of social interaction: Eye contact and relationship inferences. *Journal of Personality and Social Psychology*, *30*(1), 110–114. https://doi.org/10.1037/h0036647

Thayer, S., & Schiff, W. (1975). Eye-Contact, Facial Expression, and the Experience of Time. *The Journal of Social Psychology*, *95*(1), 117–124. https://doi.org/10.1080/00224545.1975.9923242

Torres-Marín, J., Carretero-Dios, H., Acosta, A., & Lupiáñez, J. (2017). Eye Contact and Fear of Being Laughed at in a Gaze Discrimination Task. *Frontiers in Psychology*, *8*, 1954. https://doi.org/10.3389/fpsyg.2017.01954

Trawalter, S., Todd, A. R., Baird, A. A., & Richeson, J. A. (2008). Attending to threat: Race-based patterns of selective attention. *Journal of Experimental Social Psychology*, *44*(5), 1322–1327. https://doi.org/10.1016/j.jesp.2008.03.006

Trevisan, D. A., Roberts, N., Lin, C., & Birmingham, E. (2017). How do adults and teens with self-declared Autism Spectrum Disorder experience eye contact? A qualitative analysis of first-hand accounts. *PLOS ONE*, *12*(11), e0188446. https://doi.org/10.1371/journal.pone.0188446

Tsuji, Y., & Shimada, S. (2017). Socially anxious tendencies affect neural processing of gaze perception. *Brain and Cognition*, *118*, 63–70. https://doi.org/10.1016/j.bandc.2017.08.002

Uono, S., & Hietanen, J. K. (2015). Eye Contact Perception in the West and East: A Cross-Cultural Study. *PLOS ONE*, *10*(2), e0118094. https://doi.org/10.1371/journal.pone.0118094

Vaidya, A. R., Jin, C., & Fellows, L. K. (2014). Eye spy: The predictive value of fixation patterns in detecting subtle and extreme emotions from faces. *Cognition*, *133*(2), 443–456. https://doi.org/10.1016/j.cognition.2014.07.004

Valentine, M. E., & Ehrlichman, H. (1979). Interpersonal Gaze and Helping Behavior. *The Journal of Social Psychology*, *107*(2), 193–198. https://doi.org/10.1080/00224545.1979.9922698

Vassallo, S., Cooper, S. L., & Douglas, J. M. (2009). Visual scanning in the recognition of facial affect: Is there an observer sex difference? *Journal of Vision*, *9*(3), 11–11. https://doi.org/10.1167/9.3.11

Vecera, S. P., & Rizzo, M. (2006). Eye gaze does not produce reflexive shifts of attention: Evidence from frontal-lobe damage. *Neuropsychologia*, *44*(1), 150–159. https://doi.org/10.1016/j.neuropsychologia.2005.04.010

Vuilleumier, P., George, N., Lister, V., Armony, J., & Driver, J. (2005). Effects of perceived mutual gaze and gender on face processing and recognition memory. *Visual Cognition*, *12*(1), 85–101. https://doi.org/10.1080/13506280444000120

Wallace, S., Sebastian, C., Pellicano, E., Parr, J., & Bailey, A. (2010). Face processing abilities in relatives of individuals with ASD. *Autism Research*, *3*(6), 345–349. https://doi.org/10.1002/aur.161

Watanabe, S., Kakigi, R., Koyama, S., & Kirino, E. (1999). It takes longer to recognize the eyes than the whole face in humans: *NeuroReport*, *10*(10), 2193–2198. https://doi.org/10.1097/00001756-199907130-00035

Wheeler, R. W., Baron, J. C., Michell, S., & Ginsburg, H. J. (1979). Eye contact and the perception of intelligence. *Bulletin of the Psychonomic Society*, *13*(2), 101–102. https://doi.org/10.3758/BF03335025

Wicker, B., Michel, F., Henaff, M.-A., & Decety, J. (1998). Brain Regions Involved in the Perception of Gaze: A PET Study. *NeuroImage*, *8*(2), 221–227. https://doi.org/10.1006/nimg.1998.0357

Wieser, M. J., Pauli, P., Alpers, G. W., & Mühlberger, A. (2009). Is eye to eye contact really threatening and avoided in social anxiety?—An eye-tracking and psychophysiology study. *Journal of Anxiety Disorders*, *23*(1), 93–103. https://doi.org/10.1016/j.janxdis.2008.04.004

Wyland, C., & Forgas, J. (2010). Here's looking at you kid: Mood effects on processing eye gaze as a heuristic cue. *Social Cognition*, *28*(1), 133–144.

Yorzinski, J. L., & Miller, J. (2020). Sclera color enhances gaze perception in humans. *PLOS ONE*, *15*(2), e0228275. https://doi.org/10.1371/journal.pone.0228275

Yuki, M., Maddux, W. W., & Masuda, T. (2007). Are the windows to the soul the same in the East and West? Cultural differences in using the eyes and mouth as cues to recognize emotions in Japan and the United States. *Journal of Experimental Social Psychology*, *43*(2), 303–311. https://doi.org/10.1016/j.jesp.2006.02.004

Ziaei, M., Ebner, N. C., & Burianová, H. (2017). Functional brain networks involved in gaze and emotional processing. *European Journal of Neuroscience*, *45*(2), 312–320. https://doi.org/10.1111/ejn.13464
