# Supplementary material for: Variations in the Appearance and Interpretation of Interpersonal Eye Contact in Social Categorizations and Psychiatric Populations Worldwide: A Scoping Review with a Critical Appraisal of the Literature
Source: Int J Environ Res Public Health. 2024 Aug 18;21(8):1092. doi: 10.3390/ijerph21081092 (PMC11354482; doi:10.3390/ijerph21081092)
Supplement: Supplementary file 1 [file ijerph-21-01092-s001.zip › Table S11 Outcomes Q2 - Presentations.pdf]

Table S11: Outcomes Research subquestion 2 – Presentations of eye contact in psychiatric disorders

Psychiatric Disorder	Modality	Source	Substudy
	**Frequency of eye contact**		
	Increased frequency		
Frontotemporal Dementia	Persons with behavioral-variant frontotemporal dementia display more fixations to the eyes of emotional faces	Hutchings et al., 2018	
	Decreased frequency		
Depressive disorder	Depressive persons show significant smaller total times of eye events and reveal a tendency to disengage from emotional stimuli	Figueiredo et al., 2019	
Psychopathy	Psychopathic persons show lower frequency of the initial fixations on the eye region	Gehrer et al., 2019	
Social anxiety disorder	Persons with social anxiety show less eye gaze frequency	Howell et al., 2016	
	Normal frequency		
Autism	Persons with autism show normal ratio of looking at eyes and mouth, also normal looking time at eyes and first fixations	Rutherford & Towns, 2008	
	**Duration of eye contact**		
	Longer duration		
Mental retardation	Submissive males with mental retardation maintain significantly longer eye contrast as contrasted with dominant males with mental retardation	Rago, 1977	
	Shorter duration		
Aphiasia	Persons with aphasia have shorter duration of first fixation and total fixation duration on sad faces	Ashaie & Cherney, 2020	
Posttraumatic stress disorder (PTSD)	Persons with PTSD look at sad eyes for a significantly less time, not less at neutral or happy eyes	Coll et al., 2022	
Psychopathy	Psychopathic persons show shorter absolute dwell time on the eye region	Gehrer et al., 2019	
Social anxiety disorder	Persons with social anxiety show less eye gaze duration	Howell et al., 2016	
	**Amount of gaze**		
	Increased gaze		
Autism	Better social functioning, individual characteristics and complexity of social scene is associated with increased eye gaze toward faces/head and eye regions in persons with autism	Riddiford et al., 2021	
Social anxiety disorder	Persons with social anxiety show hyper scanning (increased scan path length) of emotional faces	Horley et al., 2004	
	Decreased gaze		
Aphiasia	Persons with aphasia fixate faster on emotional faces but direct their gaze less often to happy faces	Ashaie & Cherney, 2020	
Autism	Persons with autism look significantly less to the eyes of smiling faces	Boraston et al., 2007	
Schizophrenia	During conversations, persons with schizophrenia show a smaller amount of eye gaze and inadequate levels of augmentation in eye gaze during negative emotional situations	Choi et al., 2010	
Autism	Persons with autism spend significantly less time fixating at the eye region of faces	Corden et al., 2008	
Autism	Autistic individuals respond to various gaze conditions in similar patterns to neurotypical individuals, but to a lesser extent	Crehan & Althoff, 2021	
Autism	Persons with autism exhibit less spontaneous attention toward the eye region	Galazka et al., 2019	
Autism	The greater the pupillary contagion, the less time persons with autism generally spend looking at the eye region	Galazka et al., 2019	
Schizophrenia	Persons with schizophrenia demonstrate significantly fewer saccadic eye movements when viewing faces without context	Green et al., 2008	
Autism	Persons with autism show a reduced preference for direct gaze	Kliemann et al., 2010	
Autism	The Enactive Mind hypothesis of autism in which the acquisition of embodied social cognition is derailed early on, as a result of reduced salience of stimuli such as the eye region of others	Klin et al., 2003	
Klinefelter Syndrome (47,XXY)	Persons with Klinefelter syndrome fixate less on the eye region of faces and do not show the typical tendency to first fixate on the eyes when presented with a face	Van Rijn, 2015	
	Dependencies of amount		
Borderline personality disorder	Latency of initial saccades and fixation duration correlates negatively with aggressiveness in persons with borderline personality disorder	Bertsch et al., 2017	
Mood disorders	Oxytocin administration in persons with mood disorders doesn't show significant effect on fixations in the eye region	Rutter et al., 2019	
	**Gaze avoidance**		
	Gaze avoidance and anxiety		
Social anxiety disorder	Persons with social anxiety disorder exhibit hypervigilance-avoidance of the eyes in response to emotions, primarily in the case of negative expressions	Claudino et al., 2019	
Schizophrenia	Persons with schizophrenia show faster avoidance responses to happy faces with averted gaze	de la Asuncion et al., 2015	
Social Anxiety Disorder	Testosterone administration alleviates gaze avoidance in persons with social anxiety	Enter et al., 2016	
Psychiatric disorders	Gaze aversion indicates a desire to attenuate the interpersonal experience and thereby decrease anxiety	Grumet, 1983	
Social anxiety disorder	Eye contact avoidance with emotional faces is a prominent feature in persons with social anxiety disorder	Günther et al., 2021	
Social anxiety disorder	Persons with social anxiety show avoidance of the eyes, particularly evident for angry faces	Horley et al., 2004	

Social anxiety disorder	Persons with social anxiety show an avoidance of facial features, particularly the eyes, but extensive scanning of non-features	Horley et al., 2003
Social anxiety disorder	Persons with social anxiety without fear of blushing show gaze avoidance in comparison to persons without social anxiety and persons with social anxiety with fear of blushing	Moukheiber et al., 2012
Social anxiety disorder	Persons with social anxiety show gaze avoidance	Moukheiber et al., 2010
Social anxiety disorder	Persons with social anxiety report significantly increased levels of fear and avoidance of eye contact, which decreases significantly after 8 to 12 weeks of treatment with paroxetine	Schneier et al., 2011
Social Anxiety Disorder	Persons with social anxiety disorder exhibit greater global gaze avoidance in response to a person giving both positive and negative feedback with direct gaze	Weeks et al., 2013
Social Anxiety Disorder	Persons with social anxiety disorder exhibit greater global gaze avoidance in response to a person giving both positive and negative feedback with direct gaze	Weeks et al., 2019
	Other dependencies of avoidance	
Schizophrenia	In persons with schizophrenia show more gaze avoidance and aberrant pupil diameter dilation when viewing faces with direct gaze	Jeon et al., 2022
Autism	Persons with autism show more frequent eye avoidance	Kliemann et al., 2010
Autism	Eye avoidance may be used to reduce amygdala-related hyperarousal among persons with autism	Stuart et al., 2023
	**Gaze direction in response to expressed emotion**	
	Direct gaze in response to emotion	
Borderline personality disorder	Persons with borderline personality disorder make faster saccades towards the eyes of neutral faces and slower saccades away from fearful eyes	Bertsch et al., 2017
Autism	When viewing complex emotions from faces, autistic persons fixate evenly as others on the eyes, but longer on the mouth region	Black et al., 2020
Social anxiety disorder	Persons with social anxiety orient their attention more frequently towards the eyes of emotional faces and a delays time course of eye gaze processing	Boll et al., 2016
Depressive disorder	Persons with social anxiety do not show increased eye fixations on dysphoric faces	Rutter et al., 2021
Borderline personality disorder	Persons with borderline personality disorder reflexively direct their gaze more quickly towards the eyes of emotional and neutral faces and do not adapt their fixation patterns	Seitz et al., 2012
Alzheimer's Disease	Persons with behavioral variant of Alzheimer's Disease and frontotemporal dementia spend normal dwell time on the eyes and significantly less dwell time on the mouth	Singleton et al., 2023
	Averted gaze in response to emotion	
Autism	Persons with autism attend less to the eyes for neutral faces and look more to the mouth in happy faces than in angry faces	Åsberg Johnels et al., 2016
Anxiety disorder	Persons with social anxiety show increased eye fixations in the mouth region of sad and happy faces and reduced fixations in the eye region of happy faces	Rutter et al., 2021
	**Atypical gaze**	
Autism	Atypical gaze to the eyes is best predicted by alexithymia in both autistic and non-autistic persons	Cuve et al., 2021
Psychiatric disorders	Abnormalities of eye contact are excessive blinking, depressed look, dramatic gaze, guarded gaze, absent gaze and averted gaze	Riemer, 1955

Reference list

Åsberg Johnels, J., Hovey, D., Zürcher, N., Hippolyte, L., Lemonnier, E., Gillberg, C., & Hadjikhani, N. (2017). Autism and emotional face-viewing. *Autism Research*, *10*(5), 901–910. https://doi.org/10.1002/aur.1730

Ashaie, S. A., & Cherney, L. R. (2020). Eye Tracking as a Tool to Identify Mood in Aphasia: A Feasibility Study. *Neurorehabilitation and Neural Repair*, *34*(5), 463–471. https://doi.org/10.1177/1545968320916160

Bertsch, K., Krauch, M., Stopfer, K., Haeussler, K., Herpertz, S. C., & Gamer, M. (2017). Interpersonal Threat Sensitivity in Borderline Personality Disorder: An Eye-Tracking Study. *Journal of Personality Disorders*, *31*(5), 647–670. https://doi.org/10.1521/pedi_2017_31_273

Black, M. H., Chen, N. T., Lipp, O. V., Bölte, S., & Girdler, S. (2020). Complex facial emotion recognition and atypical gaze patterns in autistic adults. *Autism*, *24*(1), 258–262. https://doi.org/10.1177/1362361319856969

Boll, S., Bartholomaeus, M., Peter, U., Lupke, U., & Gamer, M. (2016). Attentional mechanisms of social perception are biased in social phobia. *Journal of Anxiety Disorders*, *40*, 83–93. https://doi.org/10.1016/j.janxdis.2016.04.004

Boraston, Z. L., Corden, B., Miles, L. K., Skuse, D. H., & Blakemore, S.-J. (2008). Brief Report: Perception of Genuine and Posed Smiles by Individuals with Autism. *Journal of Autism and Developmental Disorders*, *38*(3), 574–580. https://doi.org/10.1007/s10803-007-0421-1

Choi, S.-H., Ku, J., Han, K., Kim, E., Kim, S. I., Park, J., & Kim, J.-J. (2010). Deficits in Eye Gaze During Negative Social Interactions in Patients With Schizophrenia. *Journal of Nervous & Mental Disease*, *198*(11), 829–835. https://doi.org/10.1097/NMD.0b013e3181f97c0d

Claudino, R. G. E., De Lima, L. K. S., De Assis, E. D. B., & Torro, N. (2019). Facial expressions and eye tracking in individuals with social anxiety disorder: A systematic review. *Psicologia: Reflexão e Crítica*, *32*(1), 9. https://doi.org/10.1186/s41155-019-0121-8

Coll, S. Y., Eustache, F., Doidy, F., Fraisse, F., Peschanski, D., Dayan, J., Gagnepain, P., & Laisney, M. (2022). Avoidance behaviour generalizes to eye processing in posttraumatic stress disorder. *European Journal of Psychotraumatology*, *13*(1), 2044661. https://doi.org/10.1080/20008198.2022.2044661

Corden, B., Chilvers, R., & Skuse, D. (2008). Avoidance of emotionally arousing stimuli predicts social–perceptual impairment in Asperger's syndrome. *Neuropsychologia*, *46*(1), 137–147. https://doi.org/10.1016/j.neuropsychologia.2007.08.005

Crehan, E. T., & Althoff, R. R. (2021). Me looking at you, looking at me: The stare-in-the-crowd effect and autism spectrum disorder. *Journal of Psychiatric Research*, *140*, 101–109. https://doi.org/10.1016/j.jpsychires.2021.05.050

Cuve, H. C., Castiello, S., Shiferaw, B., Ichijo, E., Catmur, C., & Bird, G. (2021). Alexithymia explains atypical spatiotemporal dynamics of eye gaze in autism. *Cognition*, *212*, 104710. https://doi.org/10.1016/j.cognition.2021.104710

De La Asuncion, J., Docx, L., Sabbe, B., Morrens, M., & De Bruijn, E. R. A. (2015). Converging evidence of social avoidant behavior in schizophrenia from two approach-avoidance tasks. *Journal of Psychiatric Research*, *69*, 135–141. https://doi.org/10.1016/j.jpsychires.2015.08.008

Enter, D., Terburg, D., Harrewijn, A., Spinhoven, P., & Roelofs, K. (2016). Single dose testosterone administration alleviates gaze avoidance in women with Social Anxiety Disorder. *Psychoneuroendocrinology*, *63*, 26–33. https://doi.org/10.1016/j.psyneuen.2015.09.008

Figueiredo, G. R., Ripka, W. L., Romaneli, E. F. R., & Ulbricht, L. (2019). Attentional bias for emotional faces in depressed and non-depressed individuals: An eye-tracking study. *2019 41st Annual International Conference of the IEEE Engineering in Medicine and Biology Society (EMBC)*, 5419–5422. https://doi.org/10.1109/EMBC.2019.8857878

Galazka, M. A., Åsberg Johnels, J., Zürcher, N. R., Hippolyte, L., Lemonnier, E., Billstedt, E., Gillberg, C., & Hadjikhani, N. (2019). Pupillary Contagion in Autism. *Psychological Science*, *30*(2), 309–315. https://doi.org/10.1177/0956797618809382

Gehrer, N. A., Scheeff, J., Jusyte, A., & Schönenberg, M. (2019). Impaired attention toward the eyes in psychopathic offenders: Evidence from an eye tracking study. *Behaviour Research and Therapy*, *118*, 121–129. https://doi.org/10.1016/j.brat.2019.04.009

Green, M. J., Waldron, J. H., Simpson, I., & Coltheart, M. (2008). Visual processing of social context during mental state perception in schizophrenia. *Journal of Psychiatry & Neuroscience: JPN*, *33*(1), 34–42.

Grumet, G. W. (1983). Eye Contact: The Core of Interpersonal Relatedness. *Psychiatry*, *46*(2), 172–180. https://doi.org/10.1080/00332747.1983.11024189

Günther, V., Kropidlowski, A., Schmidt, F. M., Koelkebeck, K., Kersting, A., & Suslow, T. (2021). Attentional processes during emotional face perception in social anxiety disorder: A systematic review and meta-analysis of eye-tracking findings. *Progress in Neuro-Psychopharmacology and Biological Psychiatry*, *111*, 110353. https://doi.org/10.1016/j.pnpbp.2021.110353

Horley, K., Williams, L. M., Gonsalvez, C., & Gordon, E. (2003). Social phobics do not see eye to eye: *Journal of Anxiety Disorders*, *17*(1), 33–44. https://doi.org/10.1016/S0887-6185(02)00180-9

Horley, K., Williams, L. M., Gonsalvez, C., & Gordon, E. (2004). Face to face: Visual scanpath evidence for abnormal processing of facial expressions in social phobia. *Psychiatry Research*, *127*(1–2), 43–53. https://doi.org/10.1016/j.psychres.2004.02.016

Howell, A. N., Zibulsky, D. A., Srivastav, A., & Weeks, J. W. (2016). Relations among Social Anxiety, Eye Contact Avoidance, State Anxiety, and Perception of Interaction Performance during a Live Conversation. *Cognitive Behaviour Therapy*, *45*(2), 111–122. https://doi.org/10.1080/16506073.2015.1111932

Hutchings, R., Palermo, R., Bruggemann, J., Hodges, J. R., Piguet, O., & Kumfor, F. (2018). Looking but not seeing: Increased eye fixations in behavioural-variant frontotemporal dementia. *Cortex*, *103*, 71–81. https://doi.org/10.1016/j.cortex.2018.02.011

Jeon, G., Choi, H.-S., Jung, D.-U., Moon, S., Kim, G., Kim, S.-J., Moon, J.-J., Kim, Y.-S., & Jeon, D.-W. (2022). Evaluation of the correlation between gaze avoidance and schizophrenia psychopathology with deep learning-based emotional recognition. *Asian Journal of Psychiatry*, *68*, 102974. https://doi.org/10.1016/j.ajp.2021.102974

Kliemann, D., Dziobek, I., Hatri, A., Steimke, R., & Heekeren, H. R. (2010). Atypical Reflexive Gaze Patterns on Emotional Faces in Autism Spectrum Disorders. *The Journal of Neuroscience*, *30*(37), 12281–12287. https://doi.org/10.1523/JNEUROSCI.0688-10.2010

Klin, A., Jones, W., Schultz, R., & Volkmar, F. (2003). The enactive mind, or from actions to cognition: Lessons from autism. *Philosophical Transactions of the Royal Society of London. Series B: Biological Sciences*, *358*(1430), 345–360. https://doi.org/10.1098/rstb.2002.1202

Moukheiber, A., Rautureau, G., Perez-Diaz, F., Jouvent, R., & Pelissolo, A. (2012). Gaze behaviour in social blushers. *Psychiatry Research*, *200*(2–3), 614–619. https://doi.org/10.1016/j.psychres.2012.07.017

Moukheiber, A., Rautureau, G., Perez-Diaz, F., Soussignan, R., Dubal, S., Jouvent, R., & Pelissolo, A. (2010). Gaze avoidance in social phobia: Objective measure and correlates. *Behaviour Research and Therapy*, *48*(2), 147–151. https://doi.org/10.1016/j.brat.2009.09.012

Rago, W. (1977). Eye gaze and dominance heirarchy in profoundly mentally retarded males. *American Journal of Mental Deficiency*, *82*, 145–148.

Riddiford, J. A., Enticott, P. G., Lavale, A., & Gurvich, C. (2022). Gaze and social functioning associations in autism spectrum disorder: A systematic review and META-ANALYSIS. *Autism Research*, *15*(8), 1380–1446. https://doi.org/10.1002/aur.2729

Riemer, M. D. (1955). Abnormalities of the gaze—A classification. *The Psychiatric Quarterly*, *29*(1–4), 659–672. https://doi.org/10.1007/BF01567483

Rutherford, M. D., & Towns, A. M. (2008). Scan Path Differences and Similarities During Emotion Perception in those With and Without Autism Spectrum Disorders. *Journal of Autism and Developmental Disorders*, *38*(7), 1371–1381. https://doi.org/10.1007/s10803-007-0525-7

Rutter, L. A., Norton, D. J., Brown, B. S., & Brown, T. A. (2019). A Double-Blind Placebo Controlled Study of Intranasal Oxytocin's Effect on Emotion Recognition and Visual Attention in Outpatients with Emotional Disorders. *Cognitive Therapy and Research*, *43*(3), 523–534. https://doi.org/10.1007/s10608-018-9974-x

Rutter, L. A., Norton, D. J., & Brown, T. A. (2021). Visual attention toward emotional stimuli: Anxiety symptoms correspond to distinct gaze patterns. *PLOS ONE*, *16*(5), e0250176. https://doi.org/10.1371/journal.pone.0250176

Schneier, F. R., Rodebaugh, T. L., Blanco, C., Lewin, H., & Liebowitz, M. R. (2011). Fear and avoidance of eye contact in social anxiety disorder. *Comprehensive Psychiatry*, *52*(1), 81–87. https://doi.org/10.1016/j.comppsych.2010.04.006

Seitz, K. I., Leitenstorfer, J., Krauch, M., Hillmann, K., Boll, S., Ueltzhoeffer, K., Neukel, C., Kleindienst, N., Herpertz, S. C., & Bertsch, K. (2021). An eye-tracking study of interpersonal threat sensitivity and adverse childhood experiences in borderline personality disorder. *Borderline Personality Disorder and Emotion Dysregulation*, *8*(1), 2. https://doi.org/10.1186/s40479-020-00141-7

Singleton, E. H., Fieldhouse, J. L. P., Van 'T Hooft, J. J., Scarioni, M., Van Engelen, M.-P. E., Sikkes, S. A. M., De Boer, C., Bocancea, D. I., Van Den Berg, E., Scheltens, P., Van Der Flier, W. M., Papma, J. M., Pijnenburg, Y. A. L., & Ossenkoppele, R. (2023). Social cognition deficits and biometric signatures in the behavioural variant of Alzheimer's disease. *Brain*, *146*(5), 2163–2174. https://doi.org/10.1093/brain/awac382

Stuart, N., Whitehouse, A., Palermo, R., Bothe, E., & Badcock, N. (2023). Eye Gaze in Autism Spectrum Disorder: A Review of Neural Evidence for the Eye Avoidance Hypothesis. *Journal of Autism and Developmental Disorders*, *53*(5), 1884–1905. https://doi.org/10.1007/s10803-022-05443-z

Van Rijn, S. (2015). Social Attention in 47,XXY (Klinefelter Syndrome): Visual Scanning of Facial Expressions Using Eyetracking. *Journal of the International Neuropsychological Society*, *21*(5), 364–372. https://doi.org/10.1017/S1355617715000302

Weeks, J. W., Howell, A. N., & Goldin, P. R. (2013). Gaze avoidance in social anxiety disorder. *Depression and Anxiety*, *30*(8), 749–756. https://doi.org/10.1002/da.22146

Weeks, J. W., Howell, A. N., Srivastav, A., & Goldin, P. R. (2019). "Fear guides the eyes of the beholder": Assessing gaze avoidance in social anxiety disorder via covert eye tracking of dynamic social stimuli. *Journal of Anxiety Disorders*, *65*, 56–63. https://doi.org/10.1016/j.janxdis.2019.05.005
