# Supplementary material for: Variations in the Appearance and Interpretation of Interpersonal Eye Contact in Social Categorizations and Psychiatric Populations Worldwide: A Scoping Review with a Critical Appraisal of the Literature
Source: Int J Environ Res Public Health. 2024 Aug 18;21(8):1092. doi: 10.3390/ijerph21081092 (PMC11354482; doi:10.3390/ijerph21081092)
Supplement: Supplementary file 1 [file ijerph-21-01092-s001.zip › Table S12 Outcomes Q2 - Interpretations.pdf]

Table S12: Outcomes Research subquestion 2 – Interpretations of eye contact in psychiatric disorders

Psychiatric Disorder	Modality	Source	Substudy
	**Influence of gaze direction of others on emotion perception**		
Schizophrenia	Persons with schizophrenia need more accurate emotion recognition in faces with anger with direct gaze, and fear with averted gaze	Caruana et al., 2020	
Anorexia Nervosa	Persons with recovered anorexia nervosa show no deficits in emotion recognition from faces with direct gaze	Dinkler et al., 2019	
Schizophrenia	Persons with schizophrenia are more likely to endorse eye contact when gaze is ambiguous, which is modulated by head orientation and emotion	Tso et al., 2012	
	**Influence of eye contact on behavior**		
Mental retardation	Retarded persons show smaller response latencies for avoidance and aggressive behavior when exposed to threatening-stare	Bailey et al., 1977	
Not applicable	Nonverbal behaviors such as eye contact, of both patients and psychiatrists can serve to either facilitate or hinder the patient-physician interaction	Foley & Gentile, 2010	
	**Accuracy of emotion recognition from the eye region**		
	Impairments in autism		
Autism	Persons with autism show significant impairment in recognize complex mental states from the faces, and most marked from the eyes alone	Baron-Cohen et al., 1997	Study 3
Autism	Persons with autism show significant impairment in recognize complex mental states from the eyes	Baron-Cohen et al., 2001	
Autism	Impairments in processing eyes and gaze may represent a core deficiency in persons with autism and may be central to abnormal social cognition	Itier & Batty, 2009	
Autism	Persons with autism show impairment in recognize mental states from the eyes	Kirchner et al., 2011	
Autism	Reading the Mind in the Eyes Test' performance is negatively correlated with performance IQ in persons with autism	Peñuelas-Calvo et al., 2019	
	Impairments in other disorders		
Huntington's Disease	Persons with Huntington's Disease have significantly more difficulties inferring complex mental states from people’s eyes	Eddy et al., 2012	
Borderline personality disorder	Persons with borderline personality disorder perform significantly better reading mental states from expressions in the eye region	Fertuck et al., 2009	
Frontotemporal Dementia	Persons with frontotemporal dementia (FTP) have a breakdown in key elements of the face processing network when processing the eye region	Hutchings et al., 2017	
Schizophrenia	Persons with schizophrenia show impaired recognition of complex mental states, but not of basic emotions, from eye expressions alone	Kington et al., 2000	
Psychiatric disorders	Eye contact has an important role in supplying implicit information about a psychiatric patient’s emotional state	MacDonald, 2009	
Bipolar disorder I	Persons with bipolar disorder show impairment in recognize mental states from the eye region	Marotta et al., 2017	
Alcohol use disorder	Persons with alcoholism after long-term abstinence do not show impairment in recognize mental states from the eye region	Mátyássy et al., 2006	
Posttraumatic stress disorder (PTSD)	Persons with PTSD show impairment in recognize mental states from the eye region	Schmidt & Zachariae, 2009	
Schizophrenia	Persons with schizophrenia show compromised visual integration, and this visual integration is a significant predictor of eye-contact perception	Tso et al., 2014	
Insomnia	Persons with insomnia are less accurate in recognizing angry faces in the eyes and misidentified them as fearful faces	Zhang et al., 2019	
	**Accuracy of gaze discrimination**		
	Cone of gaze		
Social anxiety disorder	Persons with social anxiety experience a wider cone of gaze	Gamer et al., 2011	
Social anxiety disorder	The more people are looking at persons with social anxiety, the wider the experienced cone of gaze	Harbort et al., 2017	Study 1
Social anxiety disorder	Persons with social anxiety show they experience a wider cone of gaze	Harbort et al., 2017	Study 2
Schizophrenia	Persons with schizophrenia are accurate at identifying direct gaze, but are more likely to misinterpret averted gaze as directed at them	Hooker & Park, 2005	
Schizophrenia	Persons with schizophrenia are accurate at identifying direct gaze but they are more likely to misinterpret averted gaze as directed at them	Hooker & Park, 2005	
Social anxiety disorder	Females with social anxiety show no difference in experienced wider cone of direct gaze than females with low social anxiety	Jun et al., 2013	
Social anxiety disorder	Males with social anxiety show a wider cone of direct gaze than males with low social anxiety	Jun et al., 2013	
Schizophrenia	Persons with schizophrenia experience a wider cone of direct gaze	Rosse et al., 1994	
Social anxiety disorder	Socially anxious persons exhibit an enhanced self-directed perception of gaze directions and demonstrate a pronounced fear of direct eye contact	Schulze et al., 2013	
Schizoptypy	Persons with schizotypy report feeling as though they are being looked at across a wider range of angles	Wastler & Lenzenweger, 2018	
Bipolar disorder	Persons with bipolar disorder over-perceive eye contact when gaze direction is ambiguous and need weaker eye-contact signal to start perceiving gaze as self-directed	Yao et al., 2018	
	Other impairments		
Autism	Persons with autism show atypical integration of social cues for orienting to gaze direction	Ashwin et al., 2015	
Autism	Persons with autism are worse at judging gaze direction with positive expressions of others and more difficulty when information from the eyes is more difficult and ambiguous	Ashwin et al., 2009	
Autism	The detection of gaze direction is less accurate in persons with autism	Forgeot et al., 2017	
Autism	Persons with autism are impaired in judgement of direction of another person's gaze	Pantelis & Kennedy, 2017	
Autism	In persons with autism, the perception of eye contact is not tuned to be finer for upright than inverted faces but is integrated across expression and gaze direction	Vida et al., 2013	
	Velocity of judgement		
Borderline personality disorder	Persons with borderline personality disorder recognize faces with averted gaze faster than faces with direct gaze	Berchio et al., 2017b	
Alzheimer's Disease	Persons with Alzheimer Disease are slower at remembering faces with direct than averted gaze	Lopis et al., 2019	

Schizophrenia	For persons with schizophrenia, faces with direct eye gaze becomes visible significantly faster than faces with averted gaze, which is the same for healthy persons	Seymour et al., 2016
	No impairments	
Schizophrenia	Schizophrenic adults do not show any specific impairment in detecting the direction of others' gaze	Franck et al., 1998
Schizophrenia	While people with schizophrenia may suffer deficits in interpreting another person's gaze, detecting averted gaze and reflexively orienting to the gazed-at location are intact	Seymour et al., 2017
	**Gaze direction to facial regions during interpretation**	
	Eye region	
Eating disorders	Persons with eating disorders and comorbid alexithymia show reduced attention to the eye region of faces which contains anger or disgust	Fujiwara et al., 2017
Social anxiety disorder	When viewing faces with ambiguous smiling faces, persons with social anxiety look earlier and longer at the eye region, whereas others preferentially look at the smiling mouth region	Gutiérrez-García et al., 2018
Huntington's Disease	Persons with Huntington's Disease perform better on emotion recognition when fixating on the eye region but not on nose or mouth region	Kordsachia et al., 2018
	Mouth region	
Schizophrenia	To identify fearful faces, in contrast to others, persons with schizophrenia rely less on eye regions and to identify happy faces, they rely more on the mouth	Lee et al., 2010
Autism	During emotional recognition, persons with autism fail to make use of information from the eye region of faces, instead rely primarily on information from the mouth	Spezio et al., 2007
	Eye and mouth region	
Schizophrenia	Persons with schizophrenia are generally slower in detecting targets that are superimposed in the eye and mouth regions of faces	Leppänen et al., 2008
Parkinson's Disease	Cognitively intact Parkinson's Disease persons scan faces with preference for mouth and eyes	Waldthaler et al., 2019
	Other facial regions	
Schizophrenia	During affect recognition, persons with schizophrenia spend less time examining the eyes and mouth and often examine other regions of the face or areas other than the face	Shimizu et al., 2000
Mild cognitive impairment	Persons with mild cognitive impairment when scanning faces, tend to look at the center of the face and spend less time fixating the mouth	Waldthaler et al., 2019
	**Eye contact and neural network**	
	Amygdala responses	
Autism	When making mentalistic inferences from the eyes, the amygdala is less activated in persons with autism	Baron-Cohen et al., 1999
Autism	Oxytocin administration increases left amygdala reactivity and the neural network involved in social cognition, which enhances emotion recognition from eyes and mouth in persons with autism	Domes et al., 2013
Schizophrenia	Persons with schizophrenia demonstrate significantly reduced amygdala responses to direct-gaze anger expressions	Pinkham et al., 2011
Posttraumatic stress disorder (PTSD)	Persons with PTSD show enhanced coupling of the amygdala and the insula within the salience network during gaze processing	Thome et al., 2014
	Gyrus responses	
Fragile X syndrome	Persons with fragile X syndrome show greater fusiform gyrus activation and in brain areas that might suggest increased task difficulty when looking at the eyes of another person	Dalton et al., 2008
Social anxiety disorder	During paroxetine treatment, a decreased neural response to eye contact is seen in inferior and middle frontal gyri, anterior cingulate, posterior cingulate, precuneus and inferior parietal lobule	Schneier et al., 2011
	EEG latencies	
ADHD	Longer N170 latencies on EEG in autistic persons when observing eye gaze besides alterations in gaze modulation and lack of face inversion effect indexed by a delayed N170	Aydin et al., 2023
Autism	Alterations in gaze modulation in persons with autism, and lack of face inversion effect indexed by a delayed N170 during EEG	Aydin et al., 2023
Bipolar disorder	Persons with bipolar disorder show diminished P200 amplitude, stronger P200 and augmented P300 responses on EEG to eye contact	Berchio et al., 2017a
	Other brain region responses	
Autism	Lack of modulation of the superior temporal sulcus (STS) region by gaze shifts that convey different intentions contributes to eye gaze processing deficits in persons with autism	Pelphrey et al., 2005
Social anxiety disorder	Persons with social anxiety show elevated neural response to eye contact in parahippocampal cortex, inferior parietal lobule, supramarginal gyrus, posterior cingulate and middle occipital cortex	Schneier et al., 2011
Schizophrenia	Persons with schizophrenia show greater neural activation in primary visual cortex and regions involved in attentional control when viewing faces	Spilka et al., 2019
Posttraumatic stress disorder (PTSD)	When viewing direct and averted gaze, females with PTSD show a more widespread connectivity of the superior colliculis and locus coeruleus, with subcortical, limbic, and frontal brain regions	Steuwe et al., 2015
Autism	Direct gaze elicits atypical activation of the Theory-of-Mind network in autism (including medial prefrontal cortex, temporoparietal junction, posterior superior temporal sulcus region, and amygdala)	von dem Hagen et al., 2014
Autism	Moving eyes do not trigger reflexive exogenous orienting in persons with autism	Zalla et al., 2016
Autism	Persons with autism show activation in the subcortical face-processing system in response to direct eye contact, in contrast to neurotypical individuals	Zürcher et al., 2013
	**Influence of gaze direction of others on attribution**	
Borderline personality disorder	In persons with borderline personality disorder, eye contact of the other is not of influence on ratings for likeability, trustworthiness or cooperativeness	Hepp et al., 2019
Psychiatric disorders	Eye contact has an important role in providing diagnostic clues to psychiatric disorders	MacDonald, 2009
Psychiatric disorders	Eye contact has an important role in delivering impactful clinical messages	MacDonald, 2009
Autism	Persons with autism give higher warmth ratings to ingroup faces with averted gazes and outgroup faces with direct gazes	Uono et al., 2021

Abbreviations: EEG = electroencephalography.

Reference list

Ashwin, C., Hietanen, J. K., & Baron-Cohen, S. (2015). Atypical integration of social cues for orienting to gaze direction in adults with autism. *Molecular Autism*, *6*(1), 5. https://doi.org/10.1186/2040-2392-6-5

Ashwin, C., Ricciardelli, P., & Baron-Cohen, S. (2009). Positive and negative gaze perception in autism spectrum conditions. *Social Neuroscience*, *4*(2), 153–164. https://doi.org/10.1080/17470910802337902

Aydin, Ü., Cañigueral, R., Tye, C., & McLoughlin, G. (2023). Face processing in young adults with autism and ADHD: An event related potentials study. *Frontiers in Psychiatry*, *14*, 1080681. https://doi.org/10.3389/fpsyt.2023.1080681

Bailey, K. G., Tipton, R. M., & Taylor, P. F. (1977). The threatening stare: Differential response latencies in mild and profoundly retarded adults. *American Journal of Mental Deficiency*, *81*(6), 599–602.

Baron-Cohen, S., Ring, H. A., Wheelwright, S., Bullmore, E. T., Brammer, M. J., Simmons, A., & Williams, S. C. R. (1999). Social intelligence in the normal and autistic brain: An fMRI study. *European Journal of Neuroscience*, *11*(6), 1891–1898. https://doi.org/10.1046/j.1460-9568.1999.00621.x

Baron-Cohen, S., Wheelwright, S., Hill, J., Raste, Y., & Plumb, I. (2001). The "Reading the Mind in the Eyes" Test Revised Version: A Study with Normal Adults, and Adults with Asperger Syndrome or High-functioning Autism. *Journal of Child Psychology and Psychiatry*, *42*(2), 241–251. https://doi.org/10.1111/1469-7610.00715

Baron-Cohen, S., Wheelwright, S., & Jolliffe, A. T. (1997). Is There a "Language of the Eyes"? Evidence from Normal Adults, and Adults with Autism or Asperger Syndrome. *Visual Cognition*, *4*(3), 311–331. https://doi.org/10.1080/713756761

Berchio, C., Piguet, C., Gentsch, K., Küng, A.-L., Rihs, T. A., Hasler, R., Aubry, J.-M., Dayer, A., Michel, C. M., & Perroud, N. (2017a). Face and gaze perception in borderline personality disorder: An electrical neuroimaging study. *Psychiatry Research: Neuroimaging*, *269*, 62–72. https://doi.org/10.1016/j.pscychresns.2017.08.011

Berchio, C., Piguet, C., Michel, C. M., Cordera, P., Rihs, T. A., Dayer, A. G., & Aubry, J.-M. (2017b). Dysfunctional gaze processing in bipolar disorder. *NeuroImage: Clinical*, *16*, 545–556. https://doi.org/10.1016/j.nicl.2017.09.006

Caruana, N., Inkley, C., & El Zein, M. (2020). Gaze direction biases emotion categorisation in schizophrenia. *Schizophrenia Research: Cognition*, *21*, 100181. https://doi.org/10.1016/j.scog.2020.100181

Dalton, K. M., Holsen, L., Abbeduto, L., & Davidson, R. J. (2008). Brain function and gaze fixation during facial-emotion processing in fragile X and autism. *Autism Research*, *1*(4), 231–239. https://doi.org/10.1002/aur.32

Dinkler, L., Rydberg Dobrescu, S., Råstam, M., Gillberg, I. C., Gillberg, C., Wentz, E., & Hadjikhani, N. (2019). Visual scanning during emotion recognition in long-term recovered anorexia nervosa: An eye-tracking study. *International Journal of Eating Disorders*, *52*(6), 691–700. https://doi.org/10.1002/eat.23066

Domes, G., Kumbier, E., Heinrichs, M., & Herpertz, S. C. (2014). Oxytocin Promotes Facial Emotion Recognition and Amygdala Reactivity in Adults with Asperger Syndrome. *Neuropsychopharmacology*, *39*(3), 698–706. https://doi.org/10.1038/npp.2013.254

Eddy, C. M., Sira Mahalingappa, S., & Rickards, H. E. (2012). Is Huntington's disease associated with deficits in theory of mind? *Acta Neurologica Scandinavica*, *126*(6), 376–383. https://doi.org/10.1111/j.1600-0404.2012.01659.x

Fertuck, E. A., Jekal, A., Song, I., Wyman, B., Morris, M. C., Wilson, S. T., Brodsky, B. S., & Stanley, B. (2009). Enhanced 'Reading the Mind in the Eyes' in borderline personality disorder compared to healthy controls. *Psychological Medicine*, *39*(12), 1979–1988. https://doi.org/10.1017/S003329170900600X

Foley, G. N., & Gentile, J. P. (2010). Nonverbal communication in psychotherapy. *Psychiatry (Edgmont (Pa.: Township))*, *7*(6), 38–44.

Forgeot d'Arc, B., Delorme, R., Zalla, T., Lefebvre, A., Amsellem, F., Moukawane, S., Letellier, L., Leboyer, M., Mouren, M.-C., & Ramus, F. (2017). Gaze direction detection in autism spectrum disorder. *Autism*, *21*(1), 100–107. https://doi.org/10.1177/1362361316630880

Franck, N., Daprati, E., Michel, F., Saoud, M., Daléry, J., Marie-Cardine, M., & Georgieff, N. (1998). Gaze discrimination is unimpaired in schizophrenia. *Psychiatry Research*, *81*(1), 67–75. https://doi.org/10.1016/S0165-1781(98)00082-1

Fujiwara, E., Kube, V. L., Rochman, D., Macrae-Korobkov, A. K., Peynenburg, V., & The University of Alberta Hospital Eating Disorder Program. (2017). Visual Attention to Ambiguous Emotional Faces in Eating Disorders: Role of Alexithymia. *European Eating Disorders Review*, *25*(6), 451–460. https://doi.org/10.1002/erv.2535

Gamer, M., Hecht, H., Seipp, N., & Hiller, W. (2011). Who is looking at me? The cone of gaze widens in social phobia. *Cognition & Emotion*, *25*(4), 756–764. https://doi.org/10.1080/02699931.2010.503117

Gutiérrez-García, A., Calvo, M. G., & Eysenck, M. W. (2018). Social anxiety and detection of facial untrustworthiness: Spatio-temporal oculomotor profiles. *Psychiatry Research*, *262*, 55–62. https://doi.org/10.1016/j.psychres.2018.01.031

Harbort, J., Spiegel, J., Witthöft, M., & Hecht, H. (2017). The effects of social pressure and emotional expression on the cone of gaze in patients with social anxiety disorder. *Journal of Behavior Therapy and Experimental Psychiatry*, *55*, 16–24. https://doi.org/10.1016/j.jbtep.2016.11.004

Hepp, J., Gebhardt, S., Kieslich, P. J., Störkel, L. M., & Niedtfeld, I. (2019). Low positive affect display mediates the association between borderline personality disorder and negative evaluations at zero acquaintance. *Borderline Personality Disorder and Emotion Dysregulation*, *6*(1), 4. https://doi.org/10.1186/s40479-019-0103-6

Hooker, C., & Park, S. (2005). You must be looking at me: The nature of gaze perception in schizophrenia patients. *Cognitive Neuropsychiatry*, *10*(5), 327–345. https://doi.org/10.1080/13546800444000083

Hutchings, R., Palermo, R., Piguet, O., & Kumfor, F. (2017). Disrupted Face Processing in Frontotemporal Dementia: A Review of the Clinical and Neuroanatomical Evidence. *Neuropsychology Review*, *27*(1), 18–30. https://doi.org/10.1007/s11065-016-9340-2

Itier, R. J., & Batty, M. (2009). Neural bases of eye and gaze processing: The core of social cognition. *Neuroscience & Biobehavioral Reviews*, *33*(6), 843–863. https://doi.org/10.1016/j.neubiorev.2009.02.004

Jun, Y. Y., Mareschal, I., Clifford, C. W. G., & Dadds, M. R. (2013). Cone of direct gaze as a marker of social anxiety in males. *Psychiatry Research*, *210*(1), 193–198. https://doi.org/10.1016/j.psychres.2013.05.020

Kington, J. M., Jones, L. A., Watt, A. A., Hopkin, E. J., & Williams, J. (2000). Impaired eye expression recognition in schizophrenia. *Journal of Psychiatric Research*, *34*(4–5), 341–347. https://doi.org/10.1016/S0022-3956(00)00029-7

Kirchner, J. C., Hatri, A., Heekeren, H. R., & Dziobek, I. (2011). Autistic Symptomatology, Face Processing Abilities, and Eye Fixation Patterns. *Journal of Autism and Developmental Disorders*, *41*(2), 158–167. https://doi.org/10.1007/s10803-010-1032-9

Kordsachia, C. C., Labuschagne, I., & Stout, J. C. (2018). Visual scanning of the eye region of human faces predicts emotion recognition performance in Huntington's disease. *Neuropsychology*, *32*(3), 356–365. https://doi.org/10.1037/neu0000424

Lee, J., Gosselin, F., Wynn, J. K., & Green, M. F. (2011). How Do Schizophrenia Patients Use Visual Information to Decode Facial Emotion? *Schizophrenia Bulletin*, *37*(5), 1001–1008. https://doi.org/10.1093/schbul/sbq006

Leppänen, J. M., Niehaus, D. J. H., Koen, L., Schoeman, R., & Emsley, R. (2008). Allocation of attention to the eye and mouth region of faces in schizophrenia patients. *Cognitive Neuropsychiatry*, *13*(6), 505–519. https://doi.org/10.1080/13546800802608452

Lopis, D., Baltazar, M., Geronikola, N., Beaucousin, V., & Conty, L. (2019). Eye contact effects on social preference and face recognition in normal ageing and in Alzheimer's disease. *Psychological Research*, *83*(6), 1292–1303. https://doi.org/10.1007/s00426-017-0955-6

MacDonald, K. (2009). Patient-Clinician Eye Contact: Social Neuroscience and Art of Clinical Engagement. *Postgraduate Medicine*, *121*(4), 136–144. https://doi.org/10.3810/pgm.2009.07.2039

Marotta, A., Delle Chiaie, R., Bernabei, L., Grasso, R., Biondi, M., & Casagrande, M. (2018). Investigating gaze processing in euthymic bipolar disorder: Impaired ability to infer mental state and intention, but preservation of social attentional orienting. *Quarterly Journal of Experimental Psychology*, *71*(10), 2041–2051. https://doi.org/10.1177/1747021817737769

Mátyássy, A., Kelemen, O., Sárközi, Z., Janka, Z., & Kéri, S. (2006). Recognition of complex mental states in patients with alcoholism after long-term abstinence. *Alcohol and Alcoholism*, *41*(5), 512–514. https://doi.org/10.1093/alcalc/agl045

Pantelis, P. C., & Kennedy, D. P. (2017). Deconstructing atypical eye gaze perception in autism spectrum disorder. *Scientific Reports*, *7*(1), 14990. https://doi.org/10.1038/s41598-017-14919-3

Pelphrey, K. A., Morris, J. P., & McCarthy, G. (2005). Neural basis of eye gaze processing deficits in autism. *Brain*, *128*(5), 1038–1048. https://doi.org/10.1093/brain/awh404

Peñuelas-Calvo, I., Sareen, A., Sevilla-Llewellyn-Jones, J., & Fernández-Berrocal, P. (2019). The "Reading the Mind in the Eyes" Test in Autism-Spectrum Disorders Comparison with Healthy Controls: A Systematic Review and Meta-analysis. *Journal of Autism and Developmental Disorders*, *49*(3), 1048–1061. https://doi.org/10.1007/s10803-018-3814-4

Pinkham, A. E., Loughead, J., Ruparel, K., Overton, E., Gur, R. E., & Gur, R. C. (2011). Abnormal Modulation of Amygdala Activity in Schizophrenia in Response to Direct- and Averted-Gaze Threat-Related Facial Expressions. *American Journal of Psychiatry*, *168*(3), 293–301. https://doi.org/10.1176/appi.ajp.2010.10060832

Rosse, R., Kendrick, K., Wyatt, R., Isaac, A., & Deutsch, S. (1994). Gaze discrimination in patients with schizophrenia: Preliminary report. *American Journal of Psychiatry*, *151*(6), 919–921. https://doi.org/10.1176/ajp.151.6.919

Schmidt, J. Z., & Zachariae, R. (2009). PTSD and Impaired Eye Expression Recognition: A Preliminary Study. *Journal of Loss and Trauma*, *14*(1), 46–56. https://doi.org/10.1080/15325020802537096

Schneier, F. R., Pomplun, M., Sy, M., & Hirsch, J. (2011). Neural response to eye contact and paroxetine treatment in generalized social anxiety disorder. *Psychiatry Research: Neuroimaging*, *194*(3), 271–278. https://doi.org/10.1016/j.pscychresns.2011.08.006

Schulze, L., Renneberg, B., & Lobmaier, J. S. (2013). Gaze perception in social anxiety and social anxiety disorder. *Frontiers in Human Neuroscience*, *7*. https://doi.org/10.3389/fnhum.2013.00872

Seymour, K., Rhodes, G., McGuire, J., Williams, N., Jeffery, L., & Langdon, R. (2017). Assessing early processing of eye gaze in schizophrenia: Measuring the cone of direct gaze and reflexive orienting of attention. *Cognitive Neuropsychiatry*, *22*(2), 122–136. https://doi.org/10.1080/13546805.2017.1285755

Seymour, K., Rhodes, G., Stein, T., & Langdon, R. (2016). Intact unconscious processing of eye contact in schizophrenia. *Schizophrenia Research: Cognition*, *3*, 15–19. https://doi.org/10.1016/j.scog.2015.11.001

Shimizu, T., Shimizu, A., Yamashita, K., Iwase, M., Kajimoto, O., & Kawasaki, T. (2000). Comparison of Eye-Movement Patterns in Schizophrenic and Normal Adults during Examination of Facial Affect Displays. *Perceptual and Motor Skills*, *91*(3_suppl), 1045–1056. https://doi.org/10.2466/pms.2000.91.3f.1045

Spezio, M. L., Adolphs, R., Hurley, R. S. E., & Piven, J. (2007). Abnormal Use of Facial Information in High-Functioning Autism. *Journal of Autism and Developmental Disorders*, *37*(5), 929–939. https://doi.org/10.1007/s10803-006-0232-9

Spilka, M. J., Pittman, D. J., Bray, S. L., & Goghari, V. M. (2019). Manipulating visual scanpaths during facial emotion perception modulates functional brain activation in schizophrenia patients and controls. *Journal of Abnormal Psychology*, *128*(8), 855–866. https://doi.org/10.1037/abn0000468

Steuwe, C., Daniels, J. K., Frewen, P. A., Densmore, M., Theberge, J., & Lanius, R. A. (2015). Effect of direct eye contact in women with PTSD related to interpersonal trauma: Psychophysiological interaction analysis of connectivity of an innate alarm system. *Psychiatry Research: Neuroimaging*, *232*(2), 162–167. https://doi.org/10.1016/j.pscychresns.2015.02.010

Thome, J., Frewen, P., Daniels, J. K., Densmore, M., & Lanius, R. A. (2014). Altered connectivity within the salience network during direct eye gaze in PTSD. *Borderline Personality Disorder and Emotion Dysregulation*, *1*(1), 17. https://doi.org/10.1186/2051-6673-1-17

Tso, I. F., Carp, J., Taylor, S. F., & Deldin, P. J. (2014). Role of Visual Integration in Gaze Perception and Emotional Intelligence in Schizophrenia. *Schizophrenia Bulletin*, *40*(3), 617–625. https://doi.org/10.1093/schbul/sbt058

Tso, I. F., Mui, M. L., Taylor, S. F., & Deldin, P. J. (2012). Eye-contact perception in schizophrenia: Relationship with symptoms and socioemotional functioning. *Journal of Abnormal Psychology*, *121*(3), 616–627. https://doi.org/10.1037/a0026596

Uono, S., Yoshimura, S., & Toichi, M. (2021). Eye contact perception in high-functioning adults with autism spectrum disorder. *Autism*, *25*(1), 137–147. https://doi.org/10.1177/1362361320949721

Vida, M. D., Maurer, D., Calder, A. J., Rhodes, G., Walsh, J. A., Pachai, M. V., & Rutherford, M. D. (2013). The Influences of Face Inversion and Facial Expression on Sensitivity to Eye Contact in High-Functioning Adults with Autism Spectrum Disorders. *Journal of Autism and Developmental Disorders*, *43*(11), 2536–2548. https://doi.org/10.1007/s10803-013-1802-2

Von Dem Hagen, E. A. H., Stoyanova, R. S., Rowe, J. B., Baron-Cohen, S., & Calder, A. J. (2014). Direct Gaze Elicits Atypical Activation of the Theory-of-Mind Network in Autism Spectrum Conditions. *Cerebral Cortex*, *24*(6), 1485–1492. https://doi.org/10.1093/cercor/bht003

Waldthaler, J., Krüger-Zechlin, C., Stock, L., Deeb, Z., & Timmermann, L. (2019). New insights into facial emotion recognition in Parkinson's disease with and without mild cognitive impairment from visual scanning patterns. *Clinical Parkinsonism & Related Disorders*, *1*, 102–108. https://doi.org/10.1016/j.prdoa.2019.11.003

Wastler, H. M., & Lenzenweger, M. F. (2018). Cone of gaze in positive schizotypy: Relationship to referential thinking and social functioning. *Personality Disorders: Theory, Research, and Treatment*, *9*(4), 324–332. https://doi.org/10.1037/per0000258

Yao, B., Mueller, S. A., Grove, T. B., McLaughlin, M., Thakkar, K., Ellingrod, V., McInnis, M. G., Taylor, S. F., Deldin, P. J., & Tso, I. F. (2018). Eye gaze perception in bipolar disorder: Self-referential bias but intact perceptual sensitivity. *Bipolar Disorders*, *20*(1), 60–69. https://doi.org/10.1111/bdi.12564

Zalla, T., Fernandez, L. G., Pieron, M., Seassau, M., & Leboyer, M. (2016). Reduced saccadic inhibition of return to moving eyes in autism spectrum disorders. *Vision Research*, *127*, 115–121. https://doi.org/10.1016/j.visres.2016.07.008

Zhang, J., Chan, A. B., Lau, E. Y. Y., & Hsiao, J. H. (2019). Individuals with insomnia misrecognize angry faces as fearful faces while missing the eyes: An eye-tracking study. *Sleep*, *42*(2). https://doi.org/10.1093/sleep/zsy220

Zürcher, N. R., Rogier, O., Boshyan, J., Hippolyte, L., Russo, B., Gillberg, N., Helles, A., Ruest, T., Lemonnier, E., Gillberg, C., & Hadjikhani, N. (2013). Perception of Social Cues of Danger in Autism Spectrum Disorders. *PLoS ONE*, *8*(12), e81206. https://doi.org/10.1371/journal.pone.0081206
