# Supplementary material for: Variations in the Appearance and Interpretation of Interpersonal Eye Contact in Social Categorizations and Psychiatric Populations Worldwide: A Scoping Review with a Critical Appraisal of the Literature
Source: Int J Environ Res Public Health. 2024 Aug 18;21(8):1092. doi: 10.3390/ijerph21081092 (PMC11354482; doi:10.3390/ijerph21081092)
Supplement: Supplementary file 1 [file ijerph-21-01092-s001.zip › Table S9 Outcomes Q1 - Presentations.pdf]

Table S9: Outcomes Research subquestion 1 – Presentations of eye contact in social categorizations

Social categorization	Modality	Source	Substudy
	**Frequency of gaze**		
	Increased frequency		
Japan	Japanese show more frequent other-directed gazes than Australians	Elzinga, 1978	Study 1
Japan	Japanese show more frequent other-directed gazes than Australians	Elzinga, 1978	Study 2
White students, Canada	White students in Canada attend more to the eyes of White than of Black faces	Friesen et al., 2019	Study 5
Females, USA	Eye contact frequency increases significantly in females during approval seeking in comparison to approval-avoidance	Pellegrini et al., 1970	
USA	US Americans attend more to the faces and make more gaze transitions than Chinese	Stanley et al., 2013	
	Decreased frequency		
Male, Inmates with psychopathic traits, USA	Interpersonal-affective traits of psychopathy is significantly related to fewer fixations to the eyes of fear faces in male inmates	Dargis et al., 2018	
Australia	Australians show less frequent other-directed gazes than Japanese	Elzinga, 1978	Study 1
Australia	Australians show less frequent other-directed gazes than Japanese	Elzinga, 1978	Study 2
Alexithymia traits, Canada	Persons with alexithymia show reduced viewing preference of facial eye regions	Fujiwara, 2018	
USA	Elated individuals have less frequent eye contact	Natale, 1977	
China	Chinese attend less to the faces and make less gaze transitions than US Americans	Stanley et al., 2013	
	Dependencies of frequency		
USA	Frequency of eye contact with an accomplice is a consequent of both the attitude toward the accomplice and the accomplice's looking behavior	Breed & Porter, 2012	
Anxiety traits, UK	The frequency of gaze aversion in a dyadic encounter doesn't increase as the anxiety of one of both the persons increases	Hobson et al., 1973	Study 1
Anxiety traits, UK	The frequency of gaze aversion in a dyadic encounter doesn't increase as the anxiety of one of both the persons increases	Hobson et al., 1973	Study 2
	**Duration of gaze**		
	Longer duration		
Males, USA	Males gaze longer than females into a females eye while lying, and shorter into males eye while lying	Burns & Kintz, 1976	
Australia	Australians show longer lasting other-directed gazes than Japanese	Elzinga, 1978	Study 1
Australia	Australians show longer lasting other-directed gazes than Japanese	Elzinga, 1978	Study 2
Australia	When in a cooperative negotiation, the average length of gaze and mutual gaze is greater than in a competitive negotiation	Foddy, 1978	
UK	Longer gaze occurs under ingratiation	Lefebvre, 1975	Study 1
USA	Persons with more social experience tend to maintain more eye contact	Libby, 1970	
USA	Elated individuals have longer eye contact	Natale, 1977	
Females, Canada	Females tend to receive a greater duration of gaze than males and females maintain longer glances towards females than males do	Schneider et al., 1977	
Patients at emergency departments, Australia	Staring is one of the cues for violence of patients at emergency departments toward nurses	Varcoe, 2008	
Social anxiety traits, Germany	Persons with social anxiety traits tend to fixate to the eye region of a face longer	Wieser et al., 2009	
	Shorter duration		
Females, UK	Females show general decline in dwell time spent on congruent gaze faces relative to incongruent gaze faces	Bayliss et al., 2012	Study 3
Females, USA	Females gaze shorter than males into a females eye while lying, and longer into males eye while lying	Burns & Kintz, 1976	
Japan	Japanese show shorter lasting other-directed gazes than Australians	Elzinga, 1978	Study 1
Japan	Japanese show shorter lasting other-directed gazes than Australians	Elzinga, 1978	Study 2
Males, Canada	Males tend to receive a smaller duration of gaze than females and males maintain shorter glances towards females than females do	Schneider et al., 1977	
	Dependencies of duration		
Not applicable	Duration of eye contact is a change product of individual looking instead of a correlation between durations of dyads looking or the duration of the encounter	Rutter et al., 1977	
	**Amount of gaze**		
	Increased gaze		
UK	There is more eye-contact between members of a dyad, the further they are	Argyle & Dean, 1965	Study 2
UK	Mutual gaze increases with distance between interaction partners	Argyle & Ingham, 1972	Study 1
UK	Total looking and mutual gaze increases with distance between interaction partners and the effect is greatest for opposite sex pairs	Argyle & Ingham, 1972	Study 2
Autistic traits, Australia	Persons with autistic traits in the attentional area show increased looking at eyes	Davis et aL., 2017	
Females, USA	Females look significantly more to the other during interaction, in comparison with males	Exline et al., 1965	
Males and females, USA	When speaking, persons looking significantly more to the other, in comparison with listening	Exline et al., 1965	
Females, USA	Highly affiliative females look more at one another, in contrast to males	Exline, 1963	

Lovers, USA	Lovers spend more time in pure gazing during periods of silence	Goldstein et al., 1976	
Males, Wales	Oxytocin increases gaze specifically toward the eye region	Guastella et al., 2008	
East Asians, UK	During storytelling East Asians spend significantly more time engaging in mutual gaze and with longer instances of mutual gaze, compared to Western Caucasians	Haensel et al., 2022	
Females, UK	The female advantage in facial expression recognition is related to greater female attention to the eyes	Hall et al., 2010	
Female with amygdala damage, USA	Fixations heavily increase under gaze-contingent viewing to normal fixation in females with amygdala damage	Kennedy & Adolphs, 2011	Study 2
USA	Eye contact increases significantly when giving covert reinforcement contingently	Krop et al., 1973	
UK	A higher percentage of gaze occurs under ingratiation	Lefebvre, 1975	Study 1
Females, USA	Females tend to maintain more eye contact than males	Libby, 1970	
Worldwide	Liars display more deliberate eye contact than truth tellers, whereas the amount of gaze aversion does not differ between truth tellers and liars	Mann et al., 2012	
USA	Pairs seated opposite each other in shopping centers, have significantly more mutual eye contact than pairs seated adjacent to each other	Muirhead & Goldman, 1979	
USA	Young and older adults engage in significantly more mutual eye contact than middle-aged adults when being confronted with a person in a shopping center	Muirhead & Goldman, 1979	
USA	Elated individuals engage more in eye contact	Natale, 1977	
Marital adjusted spouses, Australia	Low marital adjusted spouses look more to each other on negative messages	Noller, 1980	
Lovers, USA	Couples who love each other spend more time gazing into one another's eyes than couples who love each other to a lesser degree	Rubin, 1970	
USA	Eye contact is increased when shared attention is high and may play a corrective role in disrupting shared attention (reducing synchrony)	Wohltjen & Wheatley, 2021	
	Decreased gaze		
Extraversion traits, UK	Extraversion is associated with looking less at the other	Campbell & Rushton, 1978	
Autistic traits, Australia	Persons with autistic traits in the social area show a tendency towards reduced looking at eyes	Davis et al., 2017	
Males, USA	Males look significantly less to the other during interaction, in comparison with females	Exline et al., 1965	
Males, USA	Highly affiliative males look less at one another, in contrast to females	Exline, 1963	
Males with psychopathic traits, UK	Males with psychopathic traits show reduced attention to the eyes of others	Gillespie et al., 2017	
Western Caucasians, UK	During storytelling Western Caucasians spend significantly less time engaging in mutual gaze and with shorter instances of mutual gaze, compared to East Asians	Haensel et al., 2022	
Males, UK	The male disadvantage in facial expression recognition is related to lesser male attention to the eyes	Hall et al., 2010	
Western Caucasian	During facial emotion recognition, Westerners fixate less to the eyes in comparison with Eastern	Jack et al., 2009	
Female with amygdala damage, USA	There is less fixation to the eyes in females with amygdala damage	Kennedy & Adolphs, 2011	Study 1
Females with FMR1 premutation, USA	Females with FMR1 premutation exhibit significantly reduced eye contact during both the beginning and the end of social interaction	Klusek et al., 2018	
Males, USA	Males tend to maintain less eye contact than females	Libby, 1970	
USA	Reduced eye contact with strangers in the city is a short-term adaptation to interpersonal overload	McCauley et al., 1978	
USA	Embarrassed persons show reduced eye contact	Modigliani, 1971	
Older adult Caucasians, USA	In comparison to younger Caucasian adults, older Caucasian adults attend less towards the eye regions during facial emotion recognition task	Murphy & Isaacowitz, 2010	
USA	Persons with depressive traits make less eye contact	Natale, 1977	
Marital adjusted spouses, Australia	High marital adjusted spouses look less to each other on all types of messages	Noller, 1980	
Doctors with burn out symptoms, The Netherlands	Doctors with burn out symptoms show reduced eye contact with patients	Zantinge et al., 2009	
	Dependencies of amount		
UK	The amount of gaze of others is related to favorableness of impression	Cook & Smith, 1975	
USA	Liking and perceived communication are determinants of looking at another	Goldberg & Mettee, 1969	
UK	During dyadic conversation, the amount of eye contact displayed by both interaction partners are independent of each other and variates between individuals	Lazzerini et al., 1978	
Agressiveness traits, USA	Aggressive persons show far more control over their eye contact, in comparison to unaggressive persons	Moore & Gilliland, 1921	Study 1
Social anxiety traits, Portugal	Persons with social anxiety traits show no association between empathy and fixation time on the other person's eyes, in contrast to persons with low social anxiety traits	Moutinho et al., 2021	
White persons, USA	White persons show biased attention toward Black faces relative to White faces except when faces display an averted gaze	Trawalter et al., 2008	Study 2
	**Gaze avoidance**		
	Gaze avoidance and anxiety		
Social anxiety traits, USA	Persons with social anxiety traits show more avoidance of eye contact	Judah et al., 2019	
Anxiety traits, USA	Avoiding eye contact in an effort to regulate state anxiety is an ineffective strategy over time for individuals with higher social anxiety	Langer & Rodebaugh, 2013	
Social anxiety traits, China	Gaze avoidance in persons with social anxiety traits only occurs at the first fixation duration on the eyes during interaction	Ni et al., 2023	
Social anxiety traits, The Netherlands	Persons with social anxiety traits avoid angry faces only when it has a direct gaze, and avoid happy faces irrespectively of gaze direction	Roelofs et al., 2010	
Social anxiety traits, Germany	Socially anxious individuals have a tendency to avoid direct gaze	Schulze et al., 2013	
Australia	When anxiety increases, people show more eye contact avoidance in contrast to when anxiety decreases	Stanley & Martin, 1968	
Social anxiety traits, The Netherlands	When their moral integrity is threatened, the avoidance of angry eyes is enhanced in persons with social anxiety traits	Van Dillen et al., 2016	
	Gaze avoidance and personality		
Neuroticism traits, UK	Neuroticism is associated with gaze aversion	Campbell & Rushton, 1978	
Neuroticism traits, Finland	Higher levels of neuroticism is associated with avoidance-related, relative right-sided functional brain asymmetry	Uusberg et al., 2015	
Neuroticism traits, Finland	Neuroticism is related to behavioral direct gaze avoidance and subjective averted gaze preference	Uusberg et al., 2015	
	Other dependencies of avoidance		
USA	High galvanic skin response responsivity shows eye contact avoidance tendencies	Luborsky et al., 1963	

Autistic traits, China	Gaze avoidance in persons with autistic traits occurs at several time points in the later stage during interaction	Ni et al., 2023	
Power receivers, USA	A staring look elicits avoidance in low power perceivers and approach in high power perceivers	Weick et al., 2017	
Autistic traits, China	Persons with high autistic traits show more gaze avoidance than with low autistic traits which difference is increased in the middle and late stages of face presentation	Xue et al., 2023	
	**Gaze direction in response to expressed emotion**		
	Direct gaze in response to emotion		
Germany	Persons fixate more to the eyes of sad and angry faces, to the mouth of happy faces, and equally to eyes and mouth of fearful and neutral faces	Eisenbarth & Alpers, 2011	
USA	Performing social judgments enhances saliency of gaze motion toward the observer, even those that do not result in gaze contact	Latinus et al., 2015	
Neuroticm traits, USA	Persons with neuroticism spend longer time looking at the eyes of fearful faces	Perlman et al., 2009	
Hypomanic traits, The Netherlands	Persons with hypomanic traits displays reduced eye gaze in response to fearful gaze cues and increased eye gaze in response to happy gaze cues	Putman et al., 2007	
Postnatal mothers with affective traits, UK	Mothers with affective traits first look on neutral faces of babies with direct gaze when the choice is between negative and neutral	Webb & Ayers, 2019	
	Averted gaze in response to emotion		
Social anxiety traits, South Africa	Persons with social anxiety traits more rapidly avert gaze from subliminal angry eye contact	Terburg et al., 2016	Study 1
Social anxiety traits, South Africa	Testosterone abolishes the rapid averted gaze of persons with social anxiety traits being confronted with subliminal angry eye contact	Terburg et al., 2016	Study 2
	Shifting of attention		
Anxiety traits, UK	Eye gaze in fearful, but not neutral or happy, faces produces an enhanced shifting of attention in high-state anxious persons	Holmes et al., 2006	Study 1
Anxiety traits, UK	Eye gaze in angry faces produces a contralateral shifting of attention across persons with both high and low anxiety traits, whereas eye gaze in happy and neutral faces do not	Holmes et al., 2006	Study 2
UK	Eye gaze in threat-related facial expressions induces disproportionate shifts of attention, particularly in high state anxious persons	Holmes et al., 2006	Study 3
UK	Orienting to eye gaze is not potentiated in the context of a happy or neutral expression but is in the context of fearful expression	Tipples, 2006	
	**Gaze direction preferences to facial regions**		
	Eye region		
France	Eye contact has precedence over contextual information such as head orientation and visual field	Coelho et al., 2006	
UK	During interaction, eyes are scanned more by happy and neutral persons	Hills & Hill, 2018	
UK	Happy persons are more likely to attend to eyes during face perception than sad persons	Hills & Lewis, 2011	
East Asia	During facial emotion recognition, East Asians fixate more to the eyes in comparison with Westerners	Jack et al., 2009	
East Asia	When looking at faces, East Asians fixate more to the eyes than Western Caucasians and less to eyebrows and the mouth	Jack et al., 2012	Study 1
Males, USA	When males looking at faces, 43.4% of time is spent looking in the region of the eyes	Janik et al., 1978	
China	In Chinese, eyes of emotional expressions are viewed longer in Asian faces compared to White faces	Ma et al., 2022	
Young adult Caucasians, USA	In comparison to older Caucasian adults, younger Caucasian adults attend more towards the eye regions during facial emotion recognition task	Murphy & Isaacowitz, 2010	
Japan	Japanese fixate mainly on the eyes, in contrast to British, and eye fixations of Japanese people is more affected by gaze shift of a face than British	Senju et al., 2012	
Females, USA	Females gaze more often than males at the eyes of a speaker and often exhibit 'distracted' saccades directed away from the speaker and towards a background scene element	Shen & Itti, 2012	
Congenitally deaf, Japan	Deaf persons look at the eyes more frequently and for longer duration than the nose whereas hearing persons focuses on the central region of face more than the eyes	Watanabe et al., 2011	
	Central face region		
East Asia	East Asians focus more on the central region of the face when looking at faces, unlike Western Caucasian	Blais et al., 2008	
India	Indians preferred first fixation points when gazing towards someone's faces lie between the eyes and the nose tip	Chakravarthula et al., 2021	
	Local and flanking region		
Taiwan	Taiwanese have more and a more systematic shifting gaze between focal and flanking faces in comparison to US Americans	Lee et al., 2016	
USA	US Americans have less and a less systematic shifting gaze between focal and flanking faces in comparison to Taiwanese	Lee et al., 2016	
	Nose and mouth region		
Autistic traits, The Netherlands	Persons with autism traits show less dwell time to the eyes of others during interaction, and more to the lower part of the face	Hessels et al., 2018	
Social anxiety traits, The Netherlands	Persons with social anxiety traits show less dwell time to the eyes of others during interaction, and more to the lower part of the face	Hessels et al., 2018	
UK	During interaction, nose and mouth are scanned more by sad persons	Hills & Hill, 2018	
Western Caucasian	When looking at faces, Western Caucasians fixate less to the eyes than East Asians and more to eyebrows and the mouth	Jack et al., 2012	Study 1
Males, USA	When males looking at faces, 12.6% of the time is spent looking at the mouth region	Janik et al., 1978	
China	In Chinese, noses of sad emotional expressions are viewed longer in Asian faces compared to White faces, especially during the late phase of presentation	Ma et al., 2022	
UK	British fixate more on the mouth of a face, in contrast to Japanese and eye fixations of British are less affected by the gaze shift of a face than Japanese	Senju et al., 2012	
Males, USA	Males gaze more often than females at the mouth of a speaker	Shen & Itti, 2012	
Males, Australia	During facial scanning males spent significantly more time viewing the nose and mouth than the eyes in comparison to females	Vassallo et al., 2009	
Females, Australia	During facial scanning females spent significantly less time viewing the nose and mouth than the eyes in comparison to males	Vassallo et al., 2009	
	Region exploration		
Western Caucasian	Western Caucasians use a scattered triangular pattern of fixations when looking at faces, unlike East Asians	Blais et al., 2008	

Psychopathy traits, Germany	Persons with psychopathic traits show reduced face exploration and lowered emotional reactivity	Boll & Gamer, 2016	Study 1
Japan	No developmental differences are seen in gaze patterns to faces, of students, in comparison to children	Ikeda, 2023	
British born Chinese	The eye scan strategies of faces, in British born Chinese, are more closely resembled to Eastern than Western Caucasian	Kelly et al., 2011	
	**Eye behavior and conversation**		
	Eye behavior and speaking-listening		
UK	Contexts in which hesitant phases of speech, reflecting a high level of cognitive planning and associated with a lower overall level of speaker gaze, immediately precedes the speaker-switch	Beattie, 1979	
UK	The presence of gaze does not lead to a significant increase in the incidence of short switching pauses during conversations	Beattie, 1978	
UK	Significantly more immediate speaker-switches are found when gaze does not occur	Beattie, 1978	
Females, USA	When receiving high quality arguments, females give more direct gaze	Burroughs et al., 1973	
USA	Persons make more eye contact while speaking than while listening	Kendon, 1967	
USA	Speakers characteristically ends utterances by looking at the auditor with a sustained gaze and the auditor characteristically looks away as he begins to speak	Kendon, 1967	
USA	During long utterances the speaker looks at the auditor during passages of fluent speech and at the end of phrases but looks away during passages of influent speech or during hesitations	Kendon, 1967	
UK	Where a person is looking during dyadic interaction may function as a signal regulating the exchange and maintenance of speaker role	Kendon, 1967	
White adults, USA	White adults gazing towards the other while listening and gazing away from the other while speaking, which is the other way around for Black adults	LaFrance & Mayo, 1976	Study 1
Black adults, USA	Black adults gazing away from the other while listening and gazing towards the other while speaking, which is the other way around for White adults	LaFrance & Mayo, 1976	Study 1
White adults, USA	White adults gazing towards the other while listening, which is the other way around for Black adults	LaFrance & Mayo, 1976	Study 2
Black adults, USA	Black adults gazing away from the other while listening, which is the other way around for White adults	LaFrance & Mayo, 1976	Study 2
	Eye behavior during questioning		
UK	Significantly more immediate and short latency responses occur during questions terminating without gaze than with gaze	Beattie, 1978	
USA	A mutual look between unacquainted persons greatly increases the chance of continuous conversation, while making no talk unlikely	Cary, 1978	
Native English speakers, The Netherlands	Gaze direction in responses to polar questions during interaction functions as a signal of response preference in native English speakers	Kendrick & Holler, 2017	
Canada	In comparison to Trinidadians and Japanese, Canadians maintain average eye contact when answering questions and when thinking about the answers to questions, Canadians look up	McCarthy et al., 2006	
Trinidad	In comparison to Japanese and Canadians, Trinidadians maintain the most eye contact when answering questions and when thinking about the answers to questions, Trinidadians look up	McCarthy et al., 2006	
Japan	In comparison to Trinidadians and Canadians, Japanese maintain the least eye contact when answering questions and when thinking about the answers to questions, Japanese look down	McCarthy et al., 2006	
USA	Persons show less gaze toward and more avoidance of handicapped than nonhandicapped people when conversation is required	Thompson, 1982	
USA	Persons show more staring toward the handicapped than the nonhandicapped when conversation is not likely	Thompson, 1982	
USA	When observing stuttering speech, listeners tend to reduce gaze on the speaker’s eyes and increase their gaze time on the mouth	Zhang & Kalinowski, 2012	
USA	When observing stuttering speech, listeners show a similar focus on the regions of interest of eyes and mouth	Zhang & Kalinowski, 2012	
China	When observing stuttering speech, listeners gaze behaviors is more focused on the region of interests of outside	Zhang & Kalinowski, 2012	
China	When observing stuttering speech, listeners tend to reduce their gaze time on the mouth of the speaker	Zhang & Kalinowski, 2012	
	Other dependencies of eye behavior and conversation		
USA	During conversations in dyads, eye contact increases in level from low to medium intimacy, and decreases in level from medium to high intimacy	Anderson, 1976	
Canada	In conversations, Canadians look up when thinking but only when they are aware of being observed	McCarthy et al., 2008	Study 1
Canada	In conversations, Canadians look down when they know they cannot be seen	McCarthy et al., 2008	Study 2
Japanese students, Canada	In conversations, Japanese look down when thinking, both when they are aware of being observed or not	McCarthy et al., 2008	Study 3
Bilateral amygdala damage, USA	Complete amygdala lesions result in a severe reduction in direct eye contact during conversations, together with an abnormal increase in gaze to the mouth	Spezio et al., 2007	
	**Gaze cueing**		
	Gaze cueing and emotional expression		
Females, Australia	Gaze cueing stimuli produce equivalent gaze cueing effects with happy and disgusted facial expressions in the context of complex emotionally neutral stimuli	Bayliss et al., 2010	Study 1
Females, Australia	Gaze cueing in females can be modulated by emotional expression as a function of emotion context	Bayliss et al., 2010	Study 2
Caucasians with anxiety traits, Australia	No modulations of the gaze cueing effect by emotional expression is shown by anxiety traits; gaze following remains intact across levels of anxiety traits	Talipski et al., 2020	
Caucasian with depression traits, Australia	No modulations of the gaze cueing effect by emotional expression is shown in depressive traits	Talipski et al., 2020	
Caucasian with autistic traits, Australia	No modulations of the gaze cueing effect by emotional expression is shown in autistic traits	Talipski et al., 2020	
Autistic traits, Japan	Individual differences in autistic traits and emotional characteristics do not influence the magnitude of the gaze-cueing effect	Uono et al., 2022	
	Gaze cueing and other dependencies		
UK	Persons return their eyes to faces more quickly when the faces’ eyes are looking at the same object as these persons	Bayliss et al., 2012	Study 2
UK	Fixation dwell time and saccade latency as well as explicit preference judgments are influenced by exposure to episodes in which simulated joint attention incidentally emerges	Bayliss et al., 2012	Study 1
Italy	There is a strong predisposition of persons to imitate somebody else’s oculomotor behavior, even when detrimental to task performance, which is likely linked to joint attention	Ricciardelli et al., 2002	
Young adults, UK	Younger adults are faster and more congruent in gaze cueing than older adults	Slessor et al., 2008	Study 2
Older adults, UK	Older adults are slower and less congruent in gaze cueing than younger adults	Slessor et al., 2008	Study 2
	**Pupil dilation and constriction**		
Males, USA	Dilation and constriction of pupils reflects mental activity and is a measure of interest, emotion, thought processes and attitudes in males	Hess, 1965	Study 1

The Netherlands	The association between large (small) pupils and a positive (negative) impression develops over age and is absent in children	Kret, 2018
China	During emotion processing, pupil sizes, indicative of cognitive load and arousal, are smaller in Chinese	Ma et al., 2022
Males, UK	Males show smaller pupillary response to direct gaze than deviated gaze, in comparison to females	Porter et al., 2006
Females, UK	Females show greater pupillary response to direct gaze than deviated gaze, in comparison to males	Porter et al., 2006

Reference list

Anderson, D. R. (1976). Eye Contact, Topic Intimacy, and Equilibrium Theory. *The Journal of Social Psychology*, *100*(2), 313–314. https://doi.org/10.1080/00224545.1976.9711946

Argyle, M., & Dean, J. (1965). Eye-Contact, Distance and Affiliation. *Sociometry*, *28*(3), 289. https://doi.org/10.2307/2786027

Argyle, M., & Ingham, R. (1972). Gaze, Mutual Gaze, and Proximity. *Semiotica*, *6*(1). https://doi.org/10.1515/semi.1972.6.1.32

Bayliss, A. P., Murphy, E., Naughtin, C. K., Kritikos, A., Schilbach, L., & Becker, S. I. (2013). "Gaze leading": Initiating simulated joint attention influences eye movements and choice behavior. *Journal of Experimental Psychology: General*, *142*(1), 76–92. https://doi.org/10.1037/a0029286

Bayliss, A. P., Schuch, S., & Tipper, S. P. (2010). Gaze cueing elicited by emotional faces is influenced by affective context. *Visual Cognition*, *18*(8), 1214–1232. https://doi.org/10.1080/13506285.2010.484657

Beattie, G. W. (1978). Floor apportionment and gaze in conversational dyads. *British Journal of Social and Clinical Psychology*, *17*(1), 7–15. https://doi.org/10.1111/j.2044-8260.1978.tb00889.x

Beattie, G. W. (1979). Contextual constraints on the floor-apportionment function of speaker-gaze in dyadic conversations. *British Journal of Social and Clinical Psychology*, *18*(4), 391–392. https://doi.org/10.1111/j.2044-8260.1979.tb00909.x

Blais, C., Jack, R. E., Scheepers, C., Fiset, D., & Caldara, R. (2008). Culture Shapes How We Look at Faces. *PLoS ONE*, *3*(8), e3022. https://doi.org/10.1371/journal.pone.0003022

Boll, S., & Gamer, M. (2016). Psychopathic traits affect the visual exploration of facial expressions. *Biological Psychology*, *117*, 194–201. https://doi.org/10.1016/j.biopsycho.2016.03.010

Breed, G., & Porter, M. (1972). Eye Contact, Attitudes, and Attitude Change among Males. *The Journal of Genetic Psychology*, *120*(2), 211–217. https://doi.org/10.1080/00221325.1972.10532234

Burns, J. A., & Kintz, B. L. (1976). Eye contact while lying during an interview. *Bulletin of the Psychonomic Society*, *7*(1), 87–89. https://doi.org/10.3758/BF03337131

Burroughs, W., Schultz, W., & Autrey, S. (1973). Quality of Argument, Leadership Votes, and Eye Contact in Three-Person Leaderless Groups. *The Journal of Social Psychology*, *90*(1), 89–93. https://doi.org/10.1080/00224545.1973.9922624

Campbell, A., & Rushton, J. P. (1978). Bodily communication and personality. *British Journal of Social and Clinical Psychology*, *17*(1), 31–36. https://doi.org/10.1111/j.2044-8260.1978.tb00893.x

Cary, M. S. (1978). The Role of Gaze in the Initiation of Conversation. *Social Psychology*, *41*(3), 269. https://doi.org/10.2307/3033565

Chakravarthula, P. N., Tsank, Y., & Eckstein, M. P. (2021). Eye movement strategies in face ethnicity categorization vs. Face identification tasks. *Vision Research*, *186*, 59–70. https://doi.org/10.1016/j.visres.2021.05.007

Coelho, E., George, N., Conty, L., Hugueville, L., & Tijus, C. (2006). Searching for asymmetries in the detection of gaze contact versus averted gaze under different head views: A behavioral study. *Spatial Vision*, *19*(6), 529–545. https://doi.org/10.1163/156856806779194026

Cook, M., & Smith, J. M. C. (1975). The Role of Gaze in Impression Formation. *British Journal of Social and Clinical Psychology*, *14*(1), 19–25. https://doi.org/10.1111/j.2044-8260.1975.tb00144.x

Dargis, M., Wolf, R. C., & Koenigs, M. (2018). Psychopathic traits are associated with reduced fixations to the eye region of fearful faces. *Journal of Abnormal Psychology*, *127*(1), 43–50. https://doi.org/10.1037/abn0000322

Davis, J., McKone, E., Zirnsak, M., Moore, T., O'Kearney, R., Apthorp, D., & Palermo, R. (2017). Social and attention-to-detail subclusters of autistic traits differentially predict looking at eyes and face identity recognition ability. *British Journal of Psychology*, *108*(1), 191–219. https://doi.org/10.1111/bjop.12188

Eisenbarth, H., & Alpers, G. W. (2011). Happy mouth and sad eyes: Scanning emotional facial expressions. *Emotion*, *11*(4), 860–865. https://doi.org/10.1037/a0022758

Elzinga, R. (1978). Temporal Organization of Conversation. *Sociolinguistics Newsletter*, *9*(2), 29–31.

Exline, R., Gray, D., & Schuette, D. (1965). Visual behavior in a dyad as affected by interview content and sex of respondent. *Journal of Personality and Social Psychology*, *1*(3), 201–209. https://doi.org/10.1037/h0021865

Exline, R. V. (1963). Explorations in the process of person perception: Visual interaction in relation to competition, sex, and need for affiliation1. *Journal of Personality*, *31*(1), 1–20. https://doi.org/10.1111/j.1467-6494.1963.tb01836.x

Foddy, M. (1978). Patterns of Gaze in Cooperative and Competitive Negotiation. *Human Relations*, *31*(11), 925–938. https://doi.org/10.1177/001872677803101101

Friesen, J. P., Kawakami, K., Vingilis-Jaremko, L., Caprara, R., Sidhu, D. M., Williams, A., Hugenberg, K., Rodríguez-Bailón, R., Cañadas, E., & Niedenthal, P. (2019). Perceiving happiness in an intergroup context: The role of race and attention to the eyes in differentiating between true and false smiles. *Journal of Personality and Social Psychology*, *116*(3), 375–395. https://doi.org/10.1037/pspa0000139

Fujiwara, E. (2018). Looking at the eyes interferes with facial emotion recognition in alexithymia. *Journal of Abnormal Psychology*, *127*(6), 571–577. https://doi.org/10.1037/abn0000361

Gillespie, S. M., Rotshtein, P., Beech, A. R., & Mitchell, I. J. (2017). Boldness psychopathic traits predict reduced gaze toward fearful eyes in men with a history of violence. *Biological Psychology*, *128*, 29–38. https://doi.org/10.1016/j.biopsycho.2017.07.003

Goldberg, G., & Mettee, D. (1969). Liking and perceived communication potential as determinants of looking at another. *Psychonomic Science*, *16*(6), 277–278.

Goldstein, M. A., Kilroy, M. C., & Van De Voort, D. (1976). Gaze as a Function of Conversation and Degree of Love. *The Journal of Psychology*, *92*(2), 227–234. https://doi.org/10.1080/00223980.1976.9921360

Guastella, A. J., Mitchell, P. B., & Dadds, M. R. (2008). Oxytocin Increases Gaze to the Eye Region of Human Faces. *Biological Psychiatry*, *63*(1), 3–5. https://doi.org/10.1016/j.biopsych.2007.06.026

Haensel, J. X., Smith, T. J., & Senju, A. (2022). Cultural differences in mutual gaze during face-to-face interactions: A dual head-mounted eye-tracking study. *Visual Cognition*, *30*(1–2), 100–115. https://doi.org/10.1080/13506285.2021.1928354

Hall, J. K., Hutton, S. B., & Morgan, M. J. (2010). Sex differences in scanning faces: Does attention to the eyes explain female superiority in facial expression recognition? *Cognition & Emotion*, *24*(4), 629–637. https://doi.org/10.1080/02699930902906882

Hess, E. H. (1965). Attitude and Pupil Size. *Scientific American*, *212*(4), 46–54. https://doi.org/10.1038/scientificamerican0465-46

Hessels, R. S., Holleman, G. A., Cornelissen, T. H. W., Hooge, I. T. C., & Kemner, C. (2018). Eye contact takes two – autistic and social anxiety traits predict gaze behavior in dyadic interaction. *Journal of Experimental Psychopathology*, *9*(2), jep.062917. https://doi.org/10.5127/jep.062917

Hills, P. J., & Hill, D. M. (2018). Sad people are more accurate at expression identification with a smaller own-ethnicity bias than happy people. *Quarterly Journal of Experimental Psychology*, *71*(8), 1797–1806. https://doi.org/10.1080/17470218.2017.1350869

Hills, P. J., & Lewis, M. B. (2011). Sad people avoid the eyes or happy people focus on the eyes? Mood induction affects facial feature discrimination: Sad people avoid the eyes. *British Journal of Psychology*, *102*(2), 260–274. https://doi.org/10.1348/000712610X519314

Hobson, G. N., Strongman, K. T., Bull, D., & Craig, G. (1973). Anxiety and Gaze Aversion in Dyadic Encounters. *British Journal of Social and Clinical Psychology*, *12*(2), 122–129. https://doi.org/10.1111/j.2044-8260.1973.tb00857.x

Holmes, A., Richards, A., & Green, S. (2006). Anxiety and sensitivity to eye gaze in emotional faces. *Brain and Cognition*, *60*(3), 282–294. https://doi.org/10.1016/j.bandc.2005.05.002

Ikeda, S. (2023). Development of Emotion Recognition from Facial Expressions with Different Eye and Mouth Cues in Japanese People. *The Journal of Genetic Psychology*, *184*(3), 187–197. https://doi.org/10.1080/00221325.2023.2168174

Jack, R. E., Blais, C., Scheepers, C., Schyns, P. G., & Caldara, R. (2009). Cultural Confusions Show that Facial Expressions Are Not Universal. *Current Biology*, *19*(18), 1543–1548. https://doi.org/10.1016/j.cub.2009.07.051

Jack, R. E., Caldara, R., & Schyns, P. G. (2012). Internal representations reveal cultural diversity in expectations of facial expressions of emotion. *Journal of Experimental Psychology: General*, *141*(1), 19–25. https://doi.org/10.1037/a0023463

Janik, S. W., Wellens, A. R., Goldberg, M. L., & Dell'Osso, L. F. (1978). Eyes as the Center of Focus in the Visual Examination of Human Faces. *Perceptual and Motor Skills*, *47*(3), 857–858. https://doi.org/10.2466/pms.1978.47.3.857

Judah, M. R., Hager, N. M., Nako, K., & Blanchette, D. (2019). Gaze Avoidance Explains the Association Between Anxiety Sensitivity Social Concerns and Social Anxiety. *International Journal of Cognitive Therapy*, *12*(3), 205–216. https://doi.org/10.1007/s41811-019-00050-w

Kelly, D. J., Jack, R. E., Miellet, S., De Luca, E., Foreman, K., & Caldara, R. (2011). Social Experience Does Not Abolish Cultural Diversity in Eye Movements. *Frontiers in Psychology*, *2*. https://doi.org/10.3389/fpsyg.2011.00095

Kendon, A. (1967). Some functions of gaze-direction in social interaction. *Acta Psychologica*, *26*, 22–63. https://doi.org/10.1016/0001-6918(67)90005-4

Kendrick, K. H., & Holler, J. (2017). Gaze Direction Signals Response Preference in Conversation. *Research on Language and Social Interaction*, *50*(1), 12–32. https://doi.org/10.1080/08351813.2017.1262120

Kennedy, D. P., & Adolphs, R. (2011). Reprint of: Impaired fixation to eyes following amygdala damage arises from abnormal bottom-up attention. *Neuropsychologia*, *49*(4), 589–595. https://doi.org/10.1016/j.neuropsychologia.2011.02.026

Klusek, J., Ruber, A., & Roberts, J. E. (2018). Impaired eye contact in the *FMR1* premutation is not associated with social anxiety or the broad autism phenotype. *The Clinical Neuropsychologist*, *32*(7), 1337–1352. https://doi.org/10.1080/13854046.2017.1384063

Kret, M. E. (2018). The role of pupil size in communication. Is there room for learning? *Cognition and Emotion*, *32*(5), 1139–1145. https://doi.org/10.1080/02699931.2017.1370417

Krop, H., Messinger, J., & Reiner, C. (1973). Increasing eye contact by covert reinforcement. *Interpersonal Development*, *4*, 51–57.

LaFrance, M., & Mayo, C. (1976). Racial differences in gaze behavior during conversations: Two systematic observational studies. *Journal of Personality and Social Psychology*, *33*(5), 547–552. https://doi.org/10.1037/0022-3514.33.5.547

Langer, J. K., & Rodebaugh, T. L. (2013). Social Anxiety and Gaze Avoidance: Averting Gaze but not Anxiety. *Cognitive Therapy and Research*, *37*(6), 1110–1120. https://doi.org/10.1007/s10608-013-9546-z

Latinus, M., Love, S. A., Rossi, A., Parada, F. J., Huang, L., Conty, L., George, N., James, K., & Puce, A. (2015). Social decisions affect neural activity to perceived dynamic gaze. *Social Cognitive and Affective Neuroscience*, *10*(11), 1557–1567. https://doi.org/10.1093/scan/nsv049

Lazzerini, A. J., Stephenson, G. M., & Neave, H. (1978). Eye-contact in dyads: A test of the Independence Hypothesis. *British Journal of Social and Clinical Psychology*, *17*(3), 227–229. https://doi.org/10.1111/j.2044-8260.1978.tb00270.x

Lee, Y.-J., Greene, H. H., Tsai, C. W., & Chou, Y. J. (2016). Differences in Sequential Eye Movement Behavior between Taiwanese and American Viewers. *Frontiers in Psychology*, *7*. https://doi.org/10.3389/fpsyg.2016.00697

Lefebvre, L. M. (1975). Encoding and Decoding of Ingratiation in Modes of Smiling and Gaze. *British Journal of Social and Clinical Psychology*, *14*(1), 33–42. https://doi.org/10.1111/j.2044-8260.1975.tb00146.x

Libby, W. (1970). Eye contact and direction of looking as stable individual differences. *Journal of Experimental Research in Personality*, 303–312.

Luborsky, L., Blinder, B., & Mackworth, N. (1963). Eye Fixation and Recall of Pictures as a Function of GSR Responsivity. *Perceptual and Motor Skills*, *16*(2), 469–483. https://doi.org/10.2466/pms.1963.16.2.469

Ma, X., Fu, M., Zhang, X., Song, X., Becker, B., Wu, R., Xu, X., Gao, Z., Kendrick, K., & Zhao, W. (2022). Own Race Eye-Gaze Bias for All Emotional Faces but Accuracy Bias Only for Sad Expressions. *Frontiers in Neuroscience*, *16*, 852484. https://doi.org/10.3389/fnins.2022.852484

Mann, S., Vrij, A., Leal, S., Granhag, P. A., Warmelink, L., & Forrester, D. (2012). Windows to the Soul? Deliberate Eye Contact as a Cue to Deceit. *Journal of Nonverbal Behavior*, *36*(3), 205–215. https://doi.org/10.1007/s10919-012-0132-y

McCarthy, A., Lee, K., Itakura, S., & Muir, D. W. (2006). Cultural Display Rules Drive Eye Gaze During Thinking. *Journal of Cross-Cultural Psychology*, *37*(6), 717–722. https://doi.org/10.1177/0022022106292079

McCarthy, A., Lee, K., Itakura, S., & Muir, D. W. (2008). Gaze Display When Thinking Depends on Culture and Context. *Journal of Cross-Cultural Psychology*, *39*(6), 716–729. https://doi.org/10.1177/0022022108323807

McCauley, C., Coleman, G., & Fusco, P. (1978). Commuters' eye contact with strangers in city and suburban train stations: Evidence of short-term adaptation to interpersonal overload in the city. *Environmental Psychology and Nonverbal Behavior*, *2*(4), 215–225. https://doi.org/10.1007/BF01173770

Modigliani, A. (1971). Embarrassment, facework, and eye contact: Testing a theory of embarrassment. *Journal of Personality and Social Psychology*, *17*(1), 15–24. https://doi.org/10.1037/h0030460

Moore, H. T., & Gilliland, A. R. (1921). The Measurement of Aggressiveness. *Journal of Applied Psychology*, *5*(2), 97–118. https://doi.org/10.1037/h0073691

Moutinho, R., Castro, S. L., & Silva, S. (2021). Look at me: The relation between empathy and fixation on the emotional eye-region in low vs. high social anxiety. *Journal of Behavior Therapy and Experimental Psychiatry*, *70*, 101610. https://doi.org/10.1016/j.jbtep.2020.101610

Muirhead, R. D., & Goldman, M. (1979). Mutual Eye Contact as Affected by Seating Position, Sex, and Age. *The Journal of Social Psychology*, *109*(2), 201–206. https://doi.org/10.1080/00224545.1979.9924195

Murphy, N. A., & Isaacowitz, D. M. (2010). Age effects and gaze patterns in recognising emotional expressions: An in-depth look at gaze measures and covariates. *Cognition & Emotion*, *24*(3), 436–452. https://doi.org/10.1080/02699930802664623

Natale, M. (1977). Induction of mood states and their effect on gaze behaviors. *Journal of Consulting and Clinical Psychology*, *45*(5), 960–960. https://doi.org/10.1037/0022-006X.45.5.960

Ni, W., Lu, H., Wang, Q., Song, C., & Yi, L. (2023). Vigilance or avoidance: How do autistic traits and social anxiety modulate attention to the eyes? *Frontiers in Neuroscience*, *16*, 1081769. https://doi.org/10.3389/fnins.2022.1081769

Noller, P. (1980). Gaze in married couples. *Journal of Nonverbal Behavior*, *5*(2), 115–129. https://doi.org/10.1007/BF00986514

Pellegrini, R. J., Hicks, R. A., & Gordon, L. (1970). The Effect of an Approval-Seeking Induction on Eye-Contact in Dyads. *British Journal of Social and Clinical Psychology*, *9*(4), 373–374. https://doi.org/10.1111/j.2044-8260.1970.tb00987.x

Perlman, S. B., Morris, J. P., Vander Wyk, B. C., Green, S. R., Doyle, J. L., & Pelphrey, K. A. (2009). Individual Differences in Personality Predict How People Look at Faces. *PLoS ONE*, *4*(6), e5952. https://doi.org/10.1371/journal.pone.0005952

Porter, G., Hood, B. M., Troscianko, T., & Macrae, C. N. (2006). Females, but Not Males, Show Greater Pupillary Response to Direct-Than Deviated-Gaze Faces. *Perception*, *35*(8), 1129–1136. https://doi.org/10.1068/p5438

Putman, P., Saevarsson, S., & Van Honk, J. (2007). Hypomanic trait is associated with a hypovigilant automatic attentional response to social cues of danger. *Bipolar Disorders*, *9*(7), 779–783. https://doi.org/10.1111/j.1399-5618.2007.00425.x

Ricciardelli, P., Bricolo, E., Aglioti, S., & Chelazzi, L. (2002). My eyes want to look where your eyes are looking: Exploring the tendency to imitate another individual's gaze. *Neuroreport*, *13*(17), 2259–2264.

Roelofs, K., Putman, P., Schouten, S., Lange, W.-G., Volman, I., & Rinck, M. (2010). Gaze direction differentially affects avoidance tendencies to happy and angry faces in socially anxious individuals. *Behavior Research and Therapy*, *48*(4), 290–294. https://doi.org/10.1016/j.brat.2009.11.008

Rubin, Z. (1970). Measurement of romantic love. *Journal of Personality and Social Psychology*, *16*(2), 265–273. https://doi.org/10.1037/h0029841

Rutter, D. R., Stephenson, G. M., Lazzerini, A. J., Ayling, K., & White, P. A. (1977). Eye-contact: A chance product of individual Looking? *British Journal of Social and Clinical Psychology*, *16*(2), 191–192. https://doi.org/10.1111/j.2044-8260.1977.tb00216.x

Schneider, F. W., Coutts, L. M., & Garrett, W. A. (1977). Interpersonal Gaze in a Triad as a Function of Sex. *Perceptual and Motor Skills*, *44*(1), 184–184. https://doi.org/10.2466/pms.1977.44.1.184

Schulze, L., Lobmaier, J. S., Arnold, M., & Renneberg, B. (2013). All eyes on me?! Social anxiety and self-directed perception of eye gaze. *Cognition & Emotion*, *27*(7), 1305–1313. https://doi.org/10.1080/02699931.2013.773881

Senju, A., Vernetti, A., Kikuchi, Y., Akechi, H., Hasegawa, T., & Johnson, M. H. (2013). Cultural background modulates how we look at other persons' gaze. *International Journal of Behavioral Development*, *37*(2), 131–136. https://doi.org/10.1177/0165025412465360

Shen, J., & Itti, L. (2012). Top-down influences on visual attention during listening are modulated by observer sex. *Vision Research*, *65*, 62–76. https://doi.org/10.1016/j.visres.2012.06.001

Slessor, G., Phillips, L. H., & Bull, R. (2008). Age-related declines in basic social perception: Evidence from tasks assessing eye-gaze processing. *Psychology and Aging*, *23*(4), 812–822. https://doi.org/10.1037/a0014348

Spezio, M. L., Huang, P.-Y. S., Castelli, F., & Adolphs, R. (2007). Amygdala Damage Impairs Eye Contact During Conversations with Real People. *The Journal of Neuroscience*, *27*(15), 3994–3997. https://doi.org/10.1523/JNEUROSCI.3789-06.2007

Stanley, G., & Martin, D. S. (1968). Eye-contact and the recall of material involving competitive and noncompetitive associations. *Psychonomic Science*, *13*(6), 337–338. https://doi.org/10.3758/BF03342614

Stanley, J. T., Zhang, X., Fung, H. H., & Isaacowitz, D. M. (2013). Cultural differences in gaze and emotion recognition: Americans contrast more than Chinese. *Emotion*, *13*(1), 36–46. https://doi.org/10.1037/a0029209

Talipski, L. A., Bell, E., Goodhew, S. C., Dawel, A., & Edwards, M. (2021). Examining the effects of social anxiety and other individual differences on gaze-directed attentional shifts. *Quarterly Journal of Experimental Psychology*, *74*(4), 771–785. https://doi.org/10.1177/1747021820973954

Terburg, D., Syal, S., Rosenberger, L. A., Heany, S. J., Stein, D. J., & Honk, J. V. (2016). Testosterone abolishes implicit subordination in social anxiety. *Psychoneuroendocrinology*, *72*, 205–211. https://doi.org/10.1016/j.psyneuen.2016.07.203

Thompson, T. (1982). Gaze toward and avoidance of the handicapped: A field experiment. *Journal of Nonverbal Behavior*, *6*, 188–195.

Tipples, J. (2006). Fear and fearfulness potentiate automatic orienting to eye gaze. *Cognition & Emotion*, *20*(2), 309–320. https://doi.org/10.1080/02699930500405550

Trawalter, S., Todd, A. R., Baird, A. A., & Richeson, J. A. (2008). Attending to threat: Race-based patterns of selective attention. *Journal of Experimental Social Psychology*, *44*(5), 1322–1327. https://doi.org/10.1016/j.jesp.2008.03.006

Uono, S., Egashira, Y., Hayashi, S., Takada, M., Ukezono, M., & Okada, T. (2022). No Influence of Emotional Faces or Autistic Traits on Gaze-Cueing in General Population. *Frontiers in Psychology*, *13*, 864116. https://doi.org/10.3389/fpsyg.2022.864116

Uusberg, H., Allik, J., & Hietanen, J. K. (2015). Eye contact reveals a relationship between Neuroticism and anterior EEG asymmetry. *Neuropsychologia*, *73*, 161–168. https://doi.org/10.1016/j.neuropsychologia.2015.05.008

Van Dillen, L. F., Enter, D., Peters, L. P. M., Van Dijk, W. W., & Rotteveel, M. (2017). Moral fixations: The role of moral integrity and social anxiety in the selective avoidance of social threat. *Biological Psychology*, *122*, 51–58. https://doi.org/10.1016/j.biopsycho.2016.01.016

Varcoe, C. (2008). Staring, tone of voice, anxiety, mumbling, and pacing in the ED were cues for violence toward nurses. *Evidence-Based Nursing*, *11*(1), 29–29. https://doi.org/10.1136/ebn.11.1.29

Vassallo, S., Cooper, S. L., & Douglas, J. M. (2009). Visual scanning in the recognition of facial affect: Is there an observer sex difference? *Journal of Vision*, *9*(3), 11–11. https://doi.org/10.1167/9.3.11

Watanabe, K., Matsuda, T., Nishioka, T., & Namatame, M. (2011). Eye Gaze during Observation of Static Faces in Deaf People. *PLoS ONE*, *6*(2), e16919. https://doi.org/10.1371/journal.pone.0016919

Webb, R., & Ayers, S. (2019). Postnatal mental health and mothers' processing of infant emotion: An eye-tracking study. *Anxiety, Stress, & Coping*, *32*(5), 484–497. https://doi.org/10.1080/10615806.2019.1620215

Weick, M., McCall, C., & Blascovich, J. (2017). Power Moves Beyond Complementarity: A Staring Look Elicits Avoidance in Low Power Perceivers and Approach in High Power Perceivers. *Personality and Social Psychology Bulletin*, *43*(8), 1188–1201.

Wieser, M. J., Pauli, P., Alpers, G. W., & Mühlberger, A. (2009). Is eye to eye contact really threatening and avoided in social anxiety?—An eye-tracking and psychophysiology study. *Journal of Anxiety Disorders*, *23*(1), 93–103. https://doi.org/10.1016/j.janxdis.2008.04.004

Wohltjen, S., & Wheatley, T. (2021). Eye contact marks the rise and fall of shared attention in conversation. *Proceedings of the National Academy of Sciences*, *118*(37), e2106645118. https://doi.org/10.1073/pnas.2106645118

Xue, H., Zhang, L., Wang, J., Liu, W., Liu, S., & Ming, D. (2023). Dynamic eye avoidance patterns in the high autistic traits group: An eye-tracking study. *Frontiers in Psychiatry*, *14*, 1086282. https://doi.org/10.3389/fpsyt.2023.1086282

Zantinge, E. M., Verhaak, P. F., De Bakker, D. H., Van Der Meer, K., & Bensing, J. M. (2009). Does burnout among doctors affect their involvement in patients' mental health problems? A study of videotaped consultations. *BMC Family Practice*, *10*(1), 60. https://doi.org/10.1186/1471-2296-10-60

Zhang, J., & Kalinowski, J. (2012). Culture and listeners' gaze responses to stuttering. *International Journal of Language & Communication Disorders*, *47*(4), 388–397. https://doi.org/10.1111/j.1460-6984.2012.00152.x
